# Diagnostic Approach to Macrocephaly in Children

**DOI:** 10.3389/fped.2021.794069

**Published:** 2022-01-14

**Authors:** Andrea Accogli, Ana Filipa Geraldo, Gianluca Piccolo, Antonella Riva, Marcello Scala, Ganna Balagura, Vincenzo Salpietro, Francesca Madia, Mohamad Maghnie, Federico Zara, Pasquale Striano, Domenico Tortora, Mariasavina Severino, Valeria Capra

**Affiliations:** ^1^Division of Medical Genetics, Department of Medicine, McGill University Health Centre, Montreal, QC, Canada; ^2^Diagnostic Neuroradiology Unit, Imaging Department, Centro Hospitalar Vila Nova de Gaia/Espinho, Vila Nova de Gaia, Portugal; ^3^Pediatric Neurology and Neuromuscular Diseases Unit, IRCCS Giannina Gaslini Institute, Genoa, Italy; ^4^Department of Neurosciences, Rehabilitation, Ophthalmology, Genetics, Maternal and Child Health (DINOGMI), University of Genoa, Genoa, Italy; ^5^Pediatric Clinic and Endocrinology, IRCCS Istituto Giannina Gaslini, Genoa, Italy; ^6^Medical Genetics Unit, IRCCS Giannina Gaslini Institute, Genoa, Italy; ^7^Neuroradiology Unit, IRCCS Istituto Giannina Gaslini, Genoa, Italy

**Keywords:** macrocephaly, brain MRI, megalencephaly, head circumference, genetic diagnosis

## Abstract

Macrocephaly affects up to 5% of the pediatric population and is defined as an abnormally large head with an occipitofrontal circumference (OFC) >2 standard deviations (SD) above the mean for a given age and sex. Taking into account that about 2–3% of the healthy population has an OFC between 2 and 3 SD, macrocephaly is considered as “clinically relevant” when OFC is above 3 SD. This implies the urgent need for a diagnostic workflow to use in the clinical setting to dissect the several causes of increased OFC, from the benign form of familial macrocephaly and the Benign enlargement of subarachnoid spaces (BESS) to many pathological conditions, including genetic disorders. Moreover, macrocephaly should be differentiated by megalencephaly (MEG), which refers exclusively to brain overgrowth, exceeding twice the SD (3SD—“clinically relevant” megalencephaly). While macrocephaly can be isolated and benign or may be the first indication of an underlying congenital, genetic, or acquired disorder, megalencephaly is most likely due to a genetic cause. Apart from the head size evaluation, a detailed family and personal history, neuroimaging, and a careful clinical evaluation are crucial to reach the correct diagnosis. In this review, we seek to underline the clinical aspects of macrocephaly and megalencephaly, emphasizing the main differential diagnosis with a major focus on common genetic disorders. We thus provide a clinico-radiological algorithm to guide pediatricians in the assessment of children with macrocephaly.

## Introduction

Macrocephaly is a relatively common clinical condition affecting up to 5% of the pediatric population (1). It encompasses a broad range of clinical entities ranging from benign familial macrocephaly and Benign External Hydrocephalus (BEH) to more than 200 genetic disorders. Macrocephaly can also be a sign of serious acquired conditions such as progressive hydrocephalus, vascular anomalies, or intracranial masses that may necessitate urgent intervention. Therefore, it is fundamental for clinicians to recognize the clinical hallmarks of these conditions to reach the correct diagnosis. The differential diagnosis is indeed very broad requiring a systematic approach including clinical history, physical examination, and neuroradiological evaluation. Neuroimaging has classically played a major role in the evaluation of macrocephaly helping to distinguish acquired causes from congenital abnormalities (2). From a clinical point of view, some features including cutaneous and vascular anomalies and several craniofacial dysmorphisms have helped physicians to recognize specific neurogenetic disorders presenting with macrocephaly. However, the increasing use of Next Generation Sequencing (NGS) in the last decade has allowed the identification of several novel genetic disorders associated with macrocephaly, challenging their genetic diagnosis in a clinical setting.

In this review, we conducted a search of the PubMed database from January 1990 to March 2021, using the terms “macrocephaly,” “macrocrania,” “megalencephaly,” “hemimegalencephaly,” “relative macrocephaly,” “congenital/primary macrocephaly.” Additional references that were cited in relevant articles were also used.

Therefore, we gathered information in the above articles with the aim to (a) overview the definitions of macrocephaly, highlighting its significance and classification; (b) overview the most common causes of macrocephaly with a major focus on common genetic disorders; (c) provide a general diagnostic workflow to guide physicians in the differential diagnosis of macrocephaly in the clinical practice.

## Terminology and Classifications

Macrocephaly (or macrocrania) is clinically defined as an abnormally large head with an occipitofrontal circumference (OFC) >2 standard deviations (SD) above the mean or greater than the 97^th^ centile for a given age and sex (3). Of note, many children with mild macrocephaly, i.e., with an OFC between +2 and +3 SD (corresponding to the 99.7^th^ centile) (4, 5) have normal development and up to 60% of them have a familial recurrence of benign macrocephaly (6). On the other hand, children with an OFC exceeding 3 SD typically present with neurogenetic disorders characterized by intellectual disability (ID), autism spectrum disorders (ASD), and frequent comorbidities (7).

Macrocephaly identified *in utero* or at birth is called “congenital or primary macrocephaly” to distinguish from cases in which a large head only develops postnatally (i.e., secondary macrocephaly). Of note, the term “relative macrocephaly” is used to describe a head circumference <2 SD but yet disproportionately large compared to the height and weight parameters of the individual (usually <2 SD) (8).

Importantly, macrocephaly should be distinguished from megalencephaly (MEG), which refers to an oversized and overweight brain (4, 9). Indeed, in contrast to the close relationship between microcephaly and micrencephaly, MEG is only one of the possible causes of macrocephaly. Other etiologies include subdural fluid collections, hydrocephalus, intracranial masses, and skeletal dysplasias (10–12).

According to the subjacent etiology and pathophysiology, MEG has been classically divided into two groups, namely anatomical/developmental and metabolic MEG (4, 11). Anatomic/developmental MEG includes those enlarged brains due to increased size or number of neuronal cells caused by disruption of signaling pathways that regulate brain cellular proliferation, differentiation, cell cycle regulation, and survival, whereas metabolic megalencephaly results from an abnormal accumulation of metabolic substances within the cells of the brain (10).

On the other side, it is important to note that anatomical/developmental MEG does not necessarily imply macrocrania, as brain overgrowth may be unilateral or even focal, thus not impacting significantly on head circumference (13). Indeed, although anatomical/developmental MEG has been classically considered a single brain malformation, it is now recognized as a spectrum of brain overgrowth disorders, as explained in detail subsequently (10, 13, 14).

## Occipital Circumference: From Fetal to Postnatal Brain Development

The fetal human brain begins to develop during the third week of gestation and continues into early adulthood. Neural progenitor cells begin to divide and differentiate into neurons and glia, the two cell types that form the basis of the nervous system (15). By the end of the embryonic period (gestational week 10), the basics of the neural system are established. The brain structures continue to develop during pregnancy, changing their size and conformation in response to tightly regulated developmental processes controlled by multiple genetic signaling pathways and environmental factors.

Head circumference normally enlarges by approximately 1 mm per day between 26 and 32 weeks of gestation, and about 0.7 mm per day between 32 and 40 weeks (16).

The OFC of full-term babies ranges from 32 to 37 cm, corresponding to a brain weight of about 370 g. Initial postnatal brain growth follows a general rule according to which OFC increases by 2, 1, and 0.5 cm per month, during the first, second, and third trimesters, respectively, for an overall expansion of about 12 cm during the first year of life. Although a child's head growth slows during the second year, the attainment of almost 90% of adult head size is completed by the end of infancy (17). Brain is fully developed by the age of 25 with an estimated weight of about 1,500 g that corresponds to an OFC of 52–58 and 52.5–58.5 cm in females and males, respectively (3).

## Macrocephaly and Autism Spectrum Disorder

A growing body of literature has provided evidence that up to 15% of individuals with ASD have macrocephaly (18, 19). Disruption of the tight regulated processes of neurogenesis, neuronal migration, and synaptogenesis leading to impaired brain development and function, have been investigated as common pathomechanisms for both ASD and macrocephaly (20). Interestingly, an increase in neuronal numbers in prefrontal cortex of subjects with ASD and macrocephaly has been reported, suggesting excess neurogenesis/neuronal proliferation as an underlying pathomechanism for the increased cerebral size in ASD (21). In addition, abnormal laminar positioning of cortical projection neurons has been found at the brain MRI and post-mortem analysis in children diagnosed with ASD (22), underscoring the importance of a proper neuronal migration to ensure the formation of the six-layers cerebral cortex and the establishment of functional neuronal connectivity in the developing brain. Once neurons have migrated, they undergo structural changes to ultimately form synaptic connections and incorporate into functional neuronal networks for proper brain function. Inappropriate synaptic pruning or arborization, resulting in increased dendrite number and size is a further proposed mechanism linking ASD and macrocephaly (23). Therefore, dysregulation of these developmental processes due to mutations in genes involved in cell proliferation (e.g., PTEN), chromatin remodeling (e.g., CHD and KMT2 gene families), transcription and protein translation (e.g., FMR1) leads to increase head size and a spectrum of developmental features including ASD.

## Measuring the Occipital Circumference: How and When?

Measuring the head circumference is an essential component of the physical examination in pediatric practice. It could be a challenging task to carry out, especially in restless young children and in the presence of thick hair, or if the tape is not placed properly. A precise measure of the head circumference should be performed by putting the tape measure along the most prominent diameter of the occiput and the mid-forehead (OFC). Then, head circumference values should be plotted in appropriate head circumference charts and normalized for age and gender (24). Occipitofrontal circumference at birth should also be normalized for week gestation of delivery and interpreted in the relation to other birth and fetal growth parameters. Specific neonatal growth charts are available for certain populations (25–27). Currently, the most widely used growth charts until the age of 36 months are those of Centers for Disease Control and Prevention (CDC). Similarly, the WHO provides head circumference and growth charts up to 5 years of age (28). New age- and sex-appropriate US CDC charts were published in 2010 (http://www.cdc.gov/growthcharts), also providing head circumference growth charts until the age of 20. According to the American Academy of Pediatrics, OFC should be measured periodically at the well-child visits until the age of 2. In the absence of practice guidelines, the OFC measurement is often overlooked in older children (29). However, OFC measurement should be warranted in all children, regardless their age, especially in presence of clinical signs of increased intracranial pressure (ICP) and neurodevelopmental delay or neurological impairment.

## History Taking and Physical Exam

Clues to causative factors are usually found on history and examination. First, family history should be taken to identify any possible genetic syndrome associated with macrocephaly that may segregate in the family. Parental head circumference should be always measured taking into account that isolated benign megalencephaly is a familial trait in over 50% of cases (30).

Furthermore, an accurate developmental history should be taken to address if the child met all developmental milestones and there is any evidence of developmental regression and behavioral change.

Perinatal history should be reviewed since prematurity could be a risk factor for hydrocephalus.

Medical history should focus to detect possible causes of hydrocephalus (e.g., intraventricular hemorrhage, meningitis, intracranial neoplasm), presence of congenital anomalies, neurological abnormalities, skin, and vascular anomalies.

The growth rate of OFC should be carefully compared with previous values to seek any remarkable change (e.g., a sharp increase of head size may suggest an acquired cause such as hydrocephalus). It is also extremely important to compare OFC with length/height and weight to figure out whether it is an isolated macrocephaly, relative macrocephaly, or it is part of a generalized overgrowth disorder.

The first and most important step in the clinical evaluation of macrocephaly is the exclusion of raised ICP as it is a neurosurgical emergency. Symptoms and signs of increased ICP are summarized in Box 1. A bruit across the fontanelle or systemic signs of congestive heart failure may indicate an intracranial vascular malformation.

Box 1 **Symptoms and signs of increased intracranial pressure in the pediatric age.**

Progressive increase of occipital circumference

Tense or bulging fontanelle

Change in the child's behavior such as extreme irritability or sleepiness

Eye changes (crossed eyes, droopy eyelids, blurred or double vision, unequal size of eye pupils, or continuous downward gaze)

Nausea or vomiting

Gait abnormalities

Loss of consciousness

Seizure

A careful physical exam should look at the presence of organomegaly (observed in overgrowth and metabolic disorders), skin and vascular anomalies (e.g., cafè-au-lait spots, hypopigmented macules, penile flecking, cutaneous naevi, hemangiomas, and other vascular anomalies seen in neurocutanous syndromes and mTORopathies), segmental overgrowth, craniofacial dysmorphisms, skeletal anomalies, and other congenital malformations.

## Brain Imaging: When and How?

Neuroimaging has traditionally played a major role in the diagnostic work-up of macrocephaly, namely in distinguishing MEG from secondary causes of macrocephaly. However, there are currently no recommendations from the American Academy of Pediatrics or the American College of Radiology providing imaging guidelines for macrocephaly (31). Therefore, requiring brain Magnetic Resonance Imaging (MRI) in a child with macrocephaly usually relies on the individual experience of the referring physician.

Recently, Sampson et al. proposed an evaluation algorithm to decide which children with macrocephaly or OFC rapidly increasing crossing two major percentiles should benefit of a neuroimaging examination. In particular, imaging is recommended in presence of risk factors such as developmental delay (DD), neurological signs and/or symptoms, unexplained irritability, and change in feeding, or concerns of abusive head trauma. Complementary neuroimaging should be also considered in cases with facial dysmorphism, associated body overweight, and/or cutaneous or vascular hallmarks. Conversely, brain MRI may not be initially necessary in children with no risk factors or other abnormal physical features and a positive family history of macrocephaly (31). Nevertheless, clinical vigilance should be maintained even in those cases.

Regarding the choice of imaging modality, head US might be considered as the initial approach in the case of open fontanels since it does not require sedation and does not expose the patient to ionizing radiation. However, brain MRI should be considered the best modality to study these patients, as it has the highest diagnostic accuracy. “Feed and wrap” techniques should be attempted first in newborns and young infants in order to try to avoid sedation. Head CT must be considered in the setting of emergency or to evaluate bony structures in selected cases, namely the skull base, cranial vault, and craniofacial bones, due to the exposure to ionizing radiation. Finally, very low-dose head CT and/or “quick brain” MRI protocols might be used in the follow-up of secondary macrocephaly (for instance due to hydrocephalus).

## Imaging Approach to Macrocephaly

As previously mentioned, neuroimaging studies are essential to differentiate MEG from macrocephaly due to increased volume of other intracranial components or abnormal masses and this division represents the first step of the imaging assessment. Therefore, careful image inspection should be made to exclude secondary causes of macrocrania, namely hydrocephalus, enlargement of the extracranial spaces, presence of intra or extra-axial tumors or cysts or abnormal skull base/cranium configuration due to a bone dysplasia (Supplementary Figure 1).

Concerning the patients with macrocrania due to CSF expansion, it is important to separate the common Benign enlargement of subarachnoid spaces (BESS) from the concerning cases of true hydrocephalus. Indeed, the former is characterized by a rather characteristic imaging pattern with predominant enlargement of the anterior pericerebral CSF spaces, sometimes accompanied by mild to moderate ventricular enlargement. Conversely, if hydrocephalus is noted, the next step is to assess which ventricular cavities are expanded to divide obstructive from communicating hydrocephalus. It is also important to try identifying the level and cause of obstruction as well as any sign of acute decompensation and in the latter case immediately alert the referring physician.

Whenever an intracranial mass/cyst with mass effect with or without associated hydrocephalus is identified, the main goals of neuroimaging are: (i) to assess its etiology or at least its more probable benign or malignant nature, (ii) to identify any additional intracranial and/or spinal lesions or other signs of disease spread, and (iii) to guide surgical planning, if appropriate. Finally, macrocrania can be present in the setting of a bone dysplasia. Therefore, a careful evaluation of bone thickness, signal intensity and/or density, and overall morphology of the skull base, cranial vault, and facial bones, as well as delayed patency or premature fusion of cranial sutures should be systematically performed.

Once these secondary causes have been excluded, the next step consists of an attempt to distinguish between developmental/congenital MEG and metabolic MEG (Supplementary Figure 2). Of note, diffuse, bilateral, and homogeneous brain enlargement can be caused by both developmental/congenital and metabolic MEG, while only developmental/congenital MEG can present with unilateral or bilateral asymmetric brain overgrowth. Imaging abnormalities in metabolic MEG are usually dynamic, varying over time, while in developmental/congenital MEG they are mostly stable, except for myelination-related changes and possible overlapping neurodegenerative features in mTORopathies (32). Additional findings pointing toward a metabolic MEG include bilateral, symmetric areas of T1WI or T2WI/FLAIR signal abnormalities in the white matter or gray matter, as well as the presence of areas of restricted diffusion or parenchymal cysts. Moreover, midline or malformations of cortical development (MCD) are usually absent in metabolic MEG. Identification of specific metabolic causes of macrocrania often relies on a pattern recognition approach (see specific conditions below) in the setting of an appropriated clinical picture. Proton magnetic resonance spectroscopy (H1-MRS) can also have a role in the diagnosis of some of these conditions. Once metabolic MEG is excluded, anatomical/developmental brain overgrowth can be assumed and further subclassified according to the extent and location of brain enlargement, including: (i) symmetric or asymmetric bilateral involvement; (ii) complete or near complete unilateral involvement (aka hemimegalencephaly, HMEG) (33), and (iii) partial unilateral involvement of up to three cerebral lobes (aka quadrantic dysplasia, lobar HMEG or hemi-HME), affecting either predominantly the frontal lobe or the parietal-occipital lobes (34). In the latter case, the brain parenchyma typically extends to the contralateral side, a finding called the “occipital sign” (33).

“Total HMEG” has been used to describe the specific association of HMEG with overgrowth of the ipsilateral brainstem and/or cerebellum (35). Although it is rather uncommon, posterior cranial fossa structures may also be involved in other conditions of the MEG spectrum, with overgrowth of the cerebellum (with or without cerebellar cortical dysplasia) often progressing more rapidly than the cerebrum during the first 2 years of life, sometimes leading to acquired cerebellar tonsillar ectopia (36, 37).

Once brain overgrowth has been confirmed and its extension and location precisely described, associated intracranial malformations should be carefully sought. Indeed, all forms of anatomical/developmental MEG may either have a normal-appearing cortex or be associated with cortical malformations (aka dysplastic MEG) and/or white matter signal abnormalities age (37). Importantly, it has recently been discovered that dysplastic HMEG correspond indeed to large areas of focal cortical dysplasia Type II, with the size of the area dependent upon both the timing and the extent of the causative mutation (38). Midline abnormalities, involving the corpus callosum, septum pellucidum, and fornix are also common in this group of disorders (36, 39, 40) and aberrant midsagittal fibers can be also depicted on DTI running either intra or inter-hemispherically, especially on HMEG (41–43).

Finally, the basal ganglia and thalami may be also involved, either showing increased volume or abnormal morphology/definition (39, 44) and the lateral ventricle in the affected hemisphere(s) may also exhibit straightened frontal horn, colpocephalic dilatation, and global dilatation (33, 44–47). Less frequently, partial lateral ventricular collapse might also be present (39, 44).

## Overview of the Main Differential Diagnosis of Macrocephaly

### Macrocephaly Related to CSF Expansion

#### Benign Enlargement of Subarachnoid Spaces

Benign enlargement of subarachnoid spaces is the most common cause of macrocephaly in infancy with an estimated incidence of 0.5 per 1,000 live births (48). There is a male-to-female predominance and intra-familiar recurrence of “benign macrocephaly” has been reported in about 40% of cases (49).

Macrocephaly in BESS is due to an increase in subarachnoid space volume, especially along the frontal convexities, and a normal or only slight increase in the volume of the lateral ventricles. BESS is considered a self-limiting condition, clinically characterized by a rapid increase in head circumference around the age of 6 months that stabilizes at around 18 months and then resolves spontaneously by the age of 3 years (49).

Among the several pathogenic hypotheses that have been proposed, the most accredited theory is the one related to the arachnoid villi immaturity that would be unable to absorb cerebrospinal fluid (CSF) (50, 51). Indeed, maturation of the arachnoid villi is often complete by 18 months, corresponding to the time when the increase in head circumference stabilizes. The majority of infants do not show any clinical signs or symptoms of increased ICP and are physically, neurologically, and developmentally normal at follow-up. However, variable gross motor and speech delays have been reported in several studies (52).

**Figure 1:**
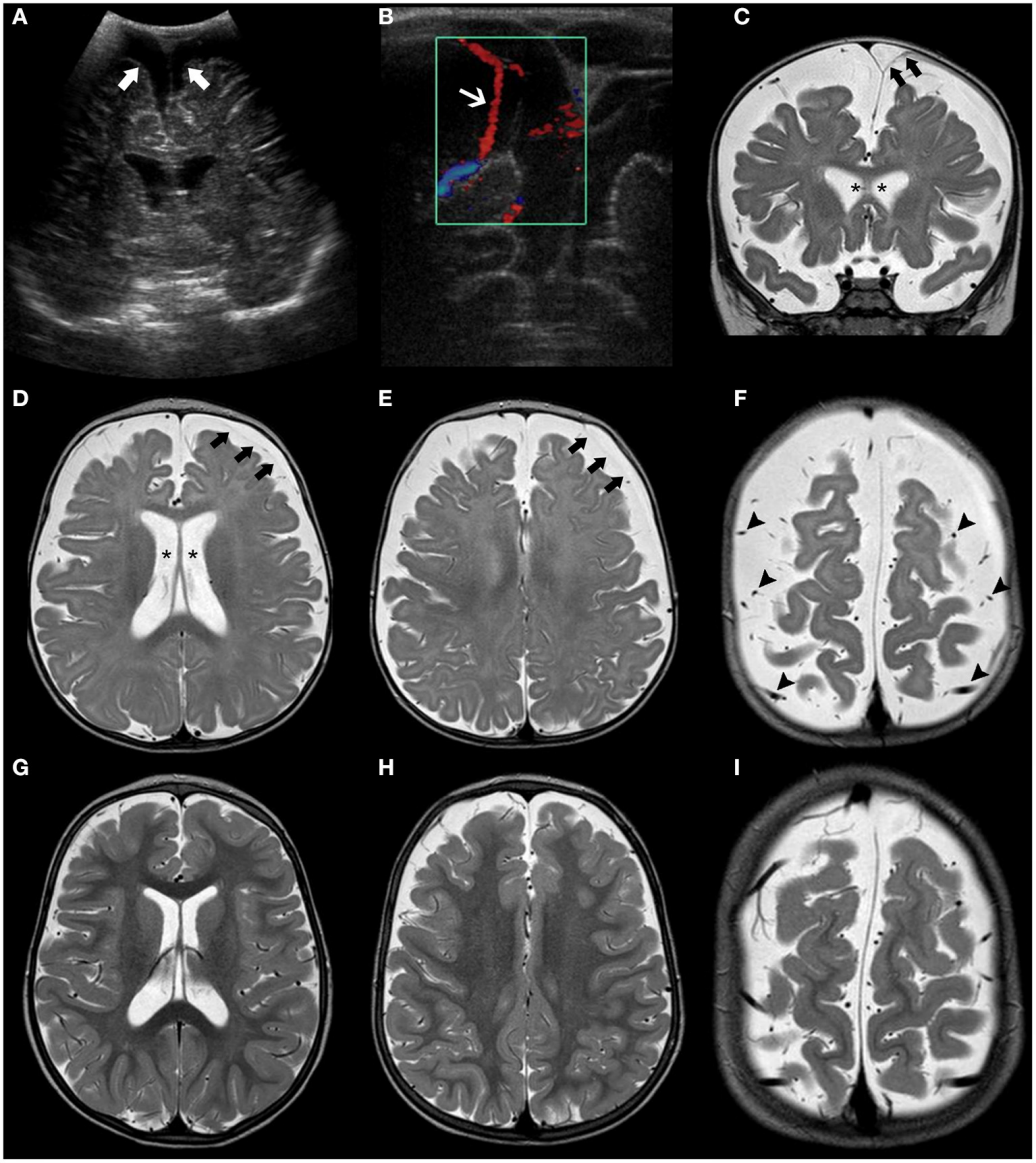
Brain MRI findings in Benign enlargement of subarachnoid spaces (BESS) of infancy. Coronal Head US image **(A)** of a 7-months-old girl with macrocephaly shows bilateral enlargement of the frontal subarachnoid spaces and anterior interhemispheric fissure (thick white arrows). Note the presence of a few vessels (white arrow) on US Doppler **(B)** crossing the enlarged subarachnoid space on the right side, corresponding to the “cortical vein sign.” Coronal **(C)** and axial **(D–F)** T2WI performed a few days after better depict these findings, including presence of multiple small vessels bilaterally within these enlarged subarachnoid spaces (black arrowheads). In addition, there is a small left frontal T2 hyperintense subdural collection (black thick arrows) as well as mild enlargement of the ventricular system (black asterisks). Follow-up brain MRI of the same patient performed at 2.3 years of age including axial T2WI **(G–I)** depict expected spontaneous interval reduction of the aforementioned enlarged subarachnoid spaces and resolution of the thin left subdural collection.

For neuroradiologists, it is challenging to define the limit between normal and enlarged subarachnoid spaces since it changes with age. However, a craniocortical width above 10 mm appears to be an overt sign of pathology (2). The earliest imaging sign is often the enlargement of the frontal interhemispheric fissure, followed by enlargement of the frontal subarachnoid spaces. Concurrent findings may include enlargement of lateral ventricles, the third ventricle, and the basal cisterns. Typically, the “cortical vein sign” (i.e., visualization of cortical veins within fluid collections at the cerebral convexities) is used to distinguish BESS from a subdural fluid collection (e.g., chronic subdural hematoma) (Figure 1). The most serious complication is subdural hematoma, likely related to stretching of the bridging veins traversing the enlarged subarachnoid spaces. As the condition is self-limiting, treatment of BESS is mostly conservative although shunting and temporary acetazolamide treatment have been adopted in some cases (51).

Follow-up brain MRI can help to establish temporal resolution and periodic clinical evaluations are warranted to ascertain there are no interval neurodevelopmental delay/regression or new neurological deficits.

#### Hydrocephalus

Despite the lack of a precise definition, hydrocephalus refers to a disorder of CSF physiology resulting in abnormal expansion of the cerebral ventricles, typically associated with increased ICP.

First, hydrocephalus should be distinguished by BESS, which presents with the aforementioned neuroradiological pattern, in absence of increased ICP signs. Several acquired and genetic causes have been associated with hydrocephalus and are listed in Supplementary Table 1 (53, 54). Based on pathophysiology, it may be due to processes that affect ventricular outflow, subarachnoid space function, cerebral venous compliance, or CSF production/reabsorption.

Traditionally, hydrocephalus has been classified into two major groups: obstructive or non-communicating hydrocephalus, which arises from an obstruction in the ventricular system, basilar cisterns, or foramen magnum, and communicating hydrocephalus that occurs when full communication exists between the ventricles and sub-arachnoid space.

Among the many causes of obstructive hydrocephalus in children, brain tumors (especially infratentorial), idiopathic aqueductal stenosis, X-linked hydrocephalus, Dandy–Walker, and Chiari malformations are the most common. Communicating hydrocephalus is rarer in children and is usually caused by deficient resorption of CSF or by an altered blood circulation within the brain, skull or chest. Further rare causes of communicating hydrocephalus include increased CSF production as seen in choroid plexus papilloma or hypertrophy (55). Of note, infectious (e.g., meningitis) and intracranial haemorrhagic lesions may cause both obstructive and communicating hydrocephalus, due to adhesions/obstructions in the subarachnoid spaces and basal cisterns or due to a reduction of CSF reabsorption, respectively. Of note, adhesions/obstructions may cause also communicating hydrocephalus when the obstruction is not at the basal cistern area.

It is crucial to distinguish on neuroimaging between acute and chronic hydrocephalus, as the former needs urgent neurosurgical evaluation and derivation procedures. Presence of diffuse effacement of the cortical sulci and increased periventricular interstitial fluid point toward an acute decompensated condition.

A thorough clinical history and physical exam could help physicians to recognize a genetic form of hydrocephalus (56, 57). For instance, familial recurrence from the maternal side may suggest X-linked hydrocephalus (MIM# 307000), the most common heritable form of hydrocephalus, due to mutations in *L1CAM*, encoding the L1 cell adhesion molecule (58). Recently, two autosomal recessive forms of hydrocephalus have been linked to mutations in *MPDZ* and *CCDC88C* that encode a tight junction protein (MUPP-1) and a regulator of cell migration (DAPLE), respectively. While the former disorders are not typically associated with significant dysmorphisms, hydrocephalus may be part of the phenotypic spectrum of many other clinically recognizable syndromes, such as Pettigrew syndrome (MIM# 304340), VACTERL-H (MIM# 314390) due to ZIC3 mutations or RASopathies (i.e., Noonan syndrome, cardio-facio-cutaneous syndrome, Costello syndrome) (59).

### Metabolic Macrocephaly

Metabolic disorders presenting with increased head size have been classically divided into three major groups: organic acid disorders, lysosomal storage disorder, and leukoencephalopathies (2, 11). As previously mentioned, when MEG is associated with these entities, it is due to the accumulation of metabolic substances within the brain, astrocyte swelling, or myelin vacuolization, while the neuronal cytoarchitecture remains relatively preserved (60). Moreover, even though some of these entities present initially megalencephaly, brain atrophy may occur later on, secondary to cell death or degeneration.

The clinical presentation of these disorders often provides hints to suspect a diagnosis and promptly pursue a proper metabolic screening. Developmental regression, decompensation during high-energy demand periods (i.e., fever, infection, prolonged fasting) should orient toward a metabolic disorder. As well, the presence of hepatosplenomegaly, spasticity, ataxia, dystonia, and a multisystemic involvement (e.g., eye, hearing, and skeletal systems) may indicate an underlying metabolic disorder. In addition, specific signs like the macular cherry-red spots in the retina in GM2 Gangliosidosis and typical neuroradiological patterns of white matter involvement may suggest a specific disorder.

We only briefly outline the main clinical and neuroradiological features of these disorders, leaving further reading of this topic to other extensive reviews (10).

#### Organic Acid Disorders

##### Glutaric Aciduria Type 1

**Figure 2:**
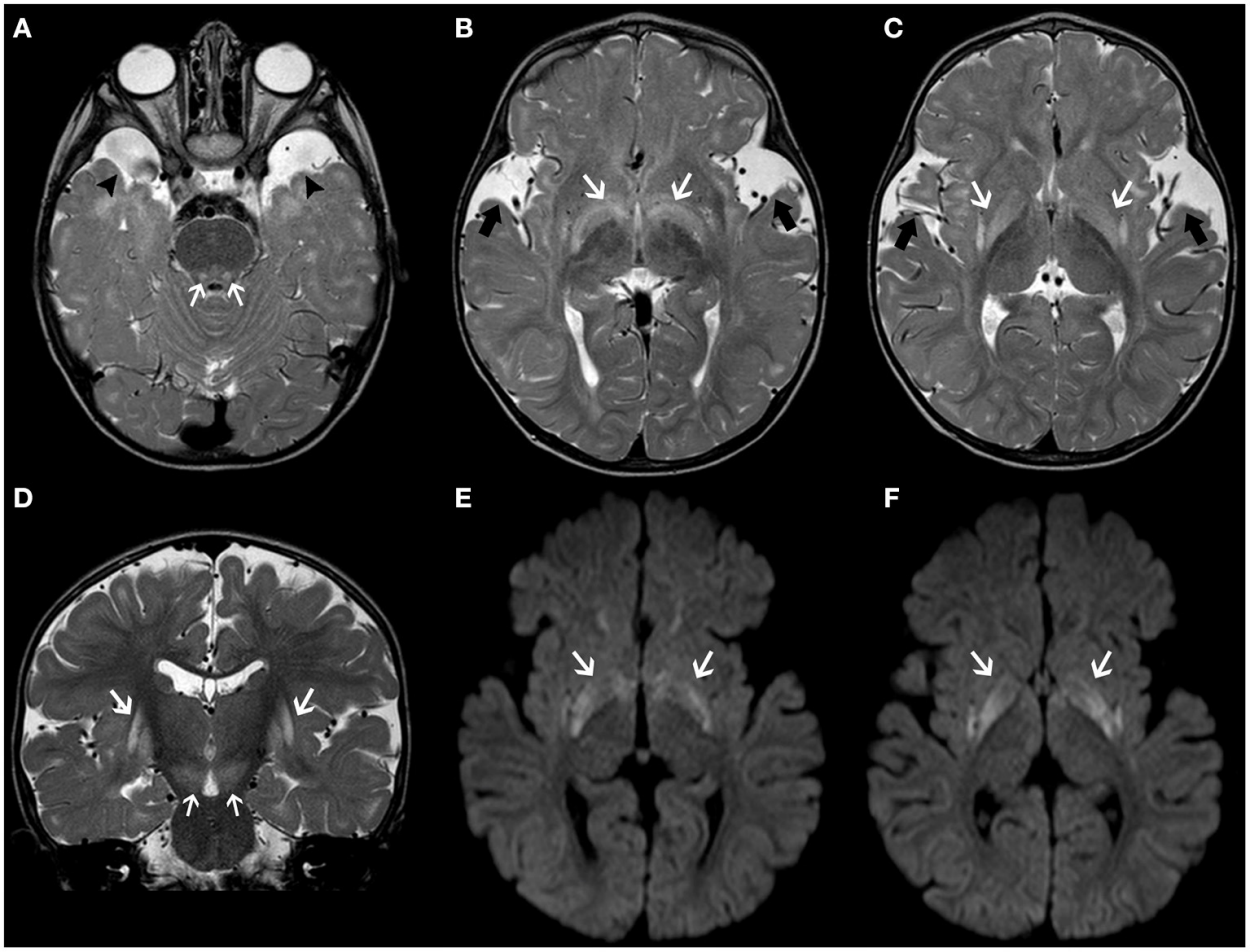
Longitudinal brain MRI findings in Glutaric Aciduria type I. Axial **(A–C)** and coronal **(D)** T2TWI of an affected 1.3-years-old boy demonstrates enlargement of the anterior temporal subarachnoid spaces (black arrowheads) and widening of the Sylvian fissures (thick black arrows) due to incomplete opercularization. There are also bilateral, symmetric hyperintensities in the globus pallidus/posterior putamen, substantia nigra and central tegmental tracts (white arrows). Axial b1000 images **(E,F)** also depict hyperintensity in the globus pallidus and posterior putamen bilaterally (white arrows). There was corresponding mild hypointensity in the ADC maps (not shown), in keeping with restricted diffusion.

Glutaric aciduria type 1 (GA1) is an autosomal recessive disorder due to a deficiency of glutaryl-CoA dehydrogenase (GCDH), a mitochondrial matrix protein involved in the catabolism of tryptophan, lysine, and hydroxylysine (61). Lack of GCDH activity mainly results in the accumulation of glutaric acid and 3-hydroxyglutaric acid which is thought to explain the neuronal vulnerability (62). Macrocephaly is often present at birth. Initial symptoms may be subtle (e.g., poor feeding and irritability), but then untreated infants experience acute encephalopathic crises triggered by catabolic events like infections (63). These crises often lead to basal ganglia damage, resulting in an irreversible dystonic–dyskinetic movement disorder (64). Typical neuroradiological features include widening of the Sylvian fissure, incomplete insular opercularization, mild ventriculomegaly, prominent pretemporal subarachnoid spaces, periventricular white matter changes, and subependymal nodules (65). Striatal abnormalities initially involve the putamen and then spread to the caudate nuclei and globus pallidus with areas of restricted diffusion during acute phases (Figure 2) (66). Subdural hygromas and hematomas may occur in a third of cases without a history of trauma and should be differentiated from abusive head trauma (67). Biochemical diagnosis of GA1 is suggested by elevated glutaric acid and 3-hydroxyglutaric acid in the urine organic acids.

Recommendations for the diagnosis and management of GA1 have recently been issued (68).

##### D2-Hydroxyglutaric Aciduria, L2-Hydroxyglutaric Aciduria

D2-hydroxyglutaric aciduria (D-2-HGA) and L2-hydroxyglutaric aciduria (L-2-HGA) are rare autosomal recessive disorders caused by deficiency of the mitochondrial enzyme D-2-hydroxyglutarate dehydrogenase and L-2-hydroxyglutarate dehydrogenase, which convert D-2-hydroxyglutarate (D-2-HG) to 2-ketoglutarate and L-2 hydroxyglutarate (L-2-HG) to alpha ketoglutarate, respectively (69). L2-hydroxyglutaric aciduria is caused by mutations in *L2HGDH*. There are two types of D-2-HGA: type I due to D2HGDH mutations (MIM# 600721) and type II linked to gain-of-function mutations in IDH2 (MIM# 613657). A combined D2-/L2-hydroxygyltaric aciduria has also been described (70).

**Figure 3:**
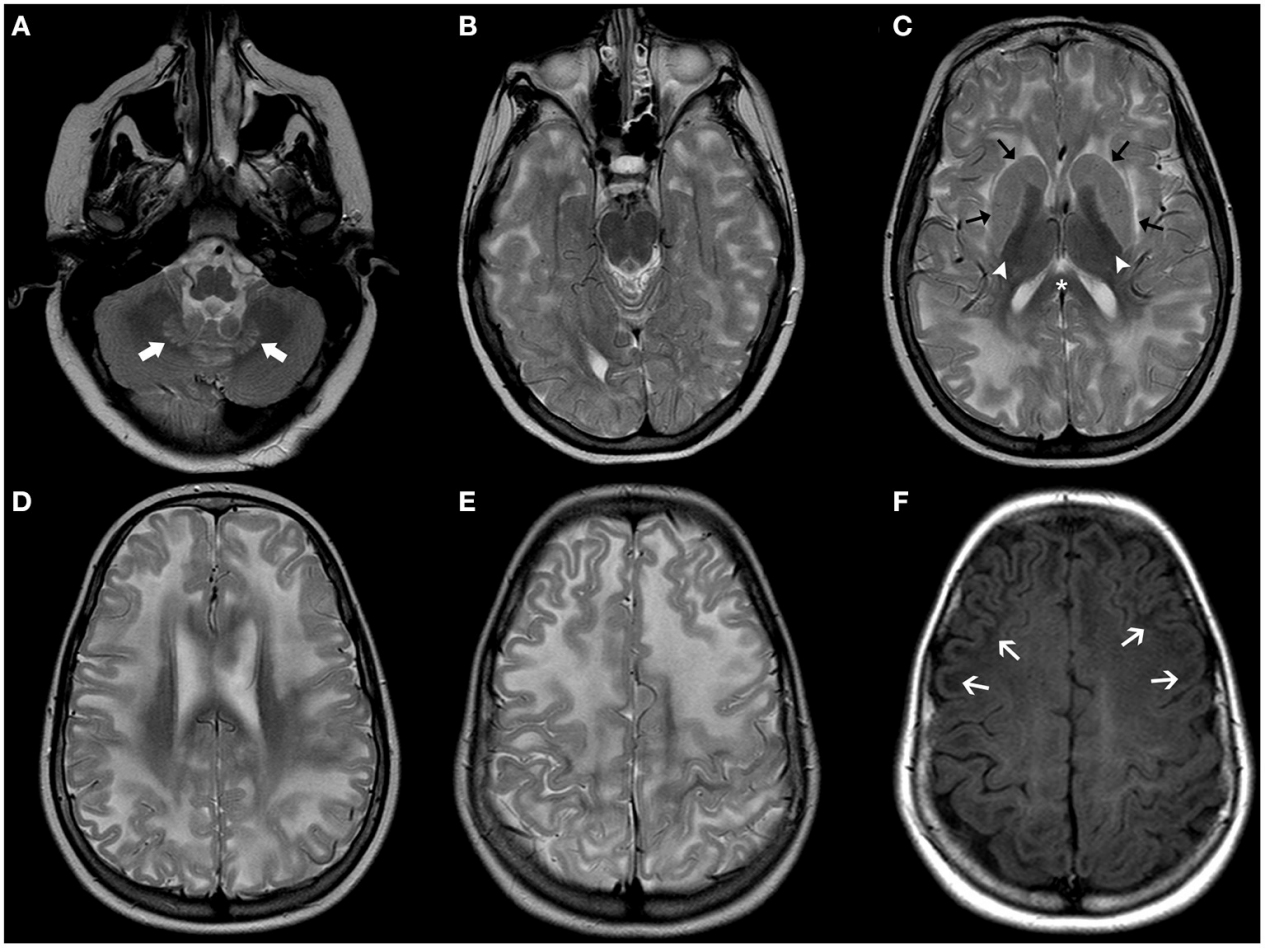
Brain MRI findings in L2-Hydroxyglutaric Aciduria. Axial T2WI **(A–E)** of an affected 11-year-old boy show diffuse, bilateral, and symmetric hyperintensity involving mainly the subcortical and deep cerebral white-matter, while the periventricular white-matter, corpus callosum (asterisks), and internal capsules (white arrowheads) are relatively spared. There is also mild, symmetric hyperintensity of the caudate nuclei and putamina (black arrows) and dentate nuclei (thick white arrows). Axial T1WI **(F)** demonstrate bilateral, symmetric hypointensity involving mainly the subcortical cerebral white matter/U fibers (white arrows).

Patients with L-2-HGA present with variable degrees of psychomotor and speech delay followed by a slowly progressive psychomotor decline. Macrocephaly is reported in half of the affected children. Slowly progressive ataxia, spasticity, dystonia, and seizures often occur (71). Common neuroradiological features include diffuse, confluent cerebral white-matter abnormalities mainly involving the subcortical region/U-fibers with frontal predominance and centripetal progression (71). The posterior fossa white matter, corpus callosum, and internal capsules are usually spared. Bilateral involvement of the basal ganglia, thalami, and dentate nucleus are also frequently reported, as well as progressive cerebral white matter atrophy over time (Figure 3) (71). There is a good correlation between clinical severity and white matter abnormalities seen on MRI (72, 73). Accumulation of L2-hydroxyglutarate is associated with an increased lifetime risk for cerebral tumors.

Cardinal clinical manifestations of D-2-HGA type I and II include DD, hypotonia, seizures, and macrocephaly. Seizure and cardiomyopathy and an overall more severe phenotype are commonly observed in type 2 D-2-HGA (69). Neuroradiological features include enlargement of the lateral ventricles (predominantly the occipital horns) and frontal subarachnoid spaces as well as subdural effusions and subependymal pseudocysts and later multifocal white-matter abnormalities (74). The diagnosis of D-2-HGA is further supported by increased levels of D-2-HG in the urine, plasma, and CSF.

#### Lysosomal Storage Disorder

##### Mucopolysaccharidosis

**Figure 4:**
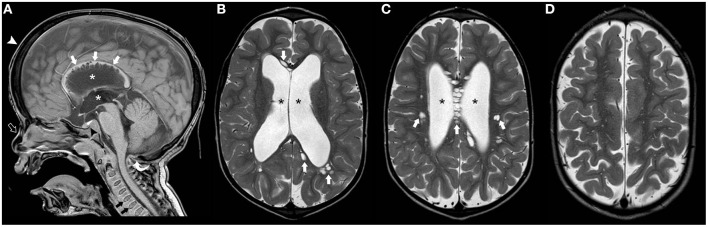
Imaging characteristics of Mucopolyssacaridosis. Sagittal T1WI **(A)** and axial T2WI **(B–D)** of a boy with Hurler syndrome performed at 4 years of age depict frontal bossing (white arrowhead), mild diffuse enlargement of the ventricular system (asterisks) as well as multiple dilated perivascular spaces (white thick arrows). Also note platybasia of the cervical vertebrae (black thick arrows), J-shaped sella (black arrowhead), flat nasal bridge (open arrow), and mild hypertrophy of the occipital squama (curved arrow), at this time point without significant stenosis of the cranio-cervical junction.

The term Mucopolysaccharidosis (MPS) refers to a group of lysosomal disorders characterized by the absence of enzymes involved in glycosaminoglycan (GAG) metabolism, resulting in the accumulation of mucopolysaccharide deposits. Seven types of MPS caused by 11 different enzymatic defects have been described (75). Apart from Hunter syndrome that follows an X-linked inheritance, all other MPS types are autosomal recessive disorders. Their clinical presentation broadly varies, depending on the type of enzyme defect and the glycoprotein accumulated. The phenotypic spectrum includes short stature, skeletal anomalies, a “coarse facial appearance,” neurological abnormalities, cardiac anomalies, breathing irregularities, and hepatosplenomegaly. Individuals with MPS often display macrocephaly that is at least in part related to ventriculomegaly and prominent subarachnoid spaces. Other neuroradiological features include focal periventricular white matter signal abnormalities, J-shaped sella and cranio-cervical junction stenosis, and vertebral anomalies (Figure 4) (76). The measurement of total GAGs excretion in urine is widely used as a biomarker for MPS (77). Guidelines for diagnosis and management of MPS have been recently reviewed (78).

#### Leukoencephalopathies

##### Alexander Disease

Alexander disease (AD) is an autosomal dominant disorder caused by mutations in *GFAP*, encoding the glial fibrillary acidic protein (OMIM# 137780). Alexander disease is characterized by diffuse demyelination with frontal lobe predominance and the presence of Rosenthal fibers, that are eosinophilic (in light microscopy) and osmophilic (in electron microscopy) inclusions consisting of intermediate filaments and irregular deposition of dense material (79).

The onset of symptoms for the majority of AD cases is before the age of 2 years (infantile form). Signs and symptoms of the infantile form typically include macrocephaly, severe DD and regression, seizures, spasticity, and ataxia. Some subjects, especially those with a neonatal presentation (neonatal form) may develop hydrocephalus with disease progression. Prognosis is poor for the neonatal and infantile forms with survival ranging from weeks to several years (80, 81). Less frequently, onset occurs later in childhood (juvenile form) or adulthood. The juvenile form usually presents between age 4 and 10 years, and it is characterized by neurodegeneration with death occurring in the 20–30 s. The adult presentation has a more variable and milder phenotype, usually in absence of macrocephaly (82).

**Figure 5:**
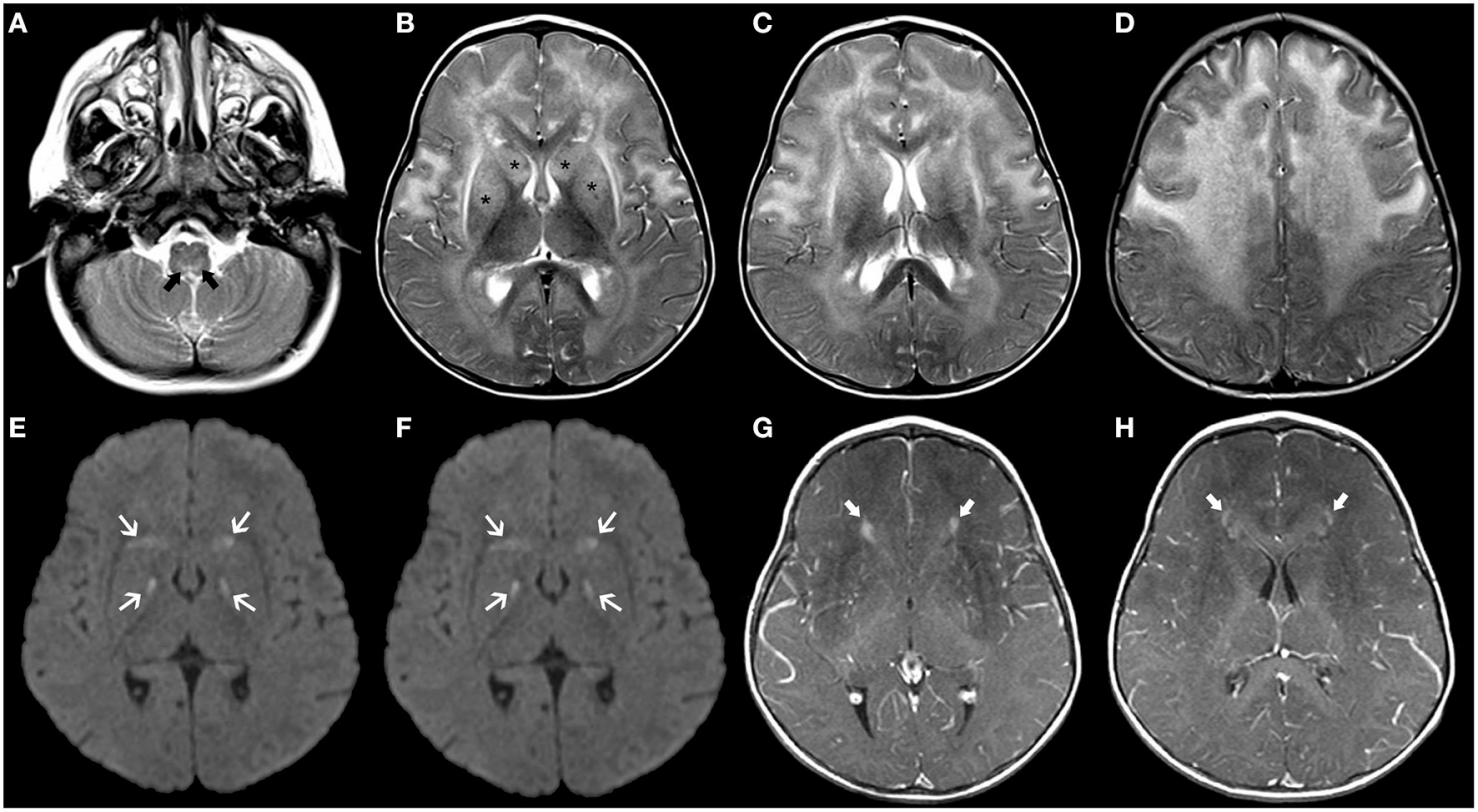
Brain MRI findings of Alexander Disease (infantile form). Axial T2WI **(A–D)** of a 1.2-years-old girl exhibit diffuse, symmetric, and confluent areas of white-matter hyperintensity with a postero-anterior gradient. Also, note areas of hyperintensity in the medulla oblongata (thick black arrows) as well as in the caudate nuclei and putamina (asterisks). Axial b1000 images **(E,F)** show focal areas of mild symmetric hyperintensity involving the globus pallidus and the head of the caudate bilaterally (white arrows). There was corresponding hypointensity in the ADC maps (not shown) in keeping with restricted diffusion. Axial post-gadolinium T1WI **(G,H)** depict small “caps” of contrast enhancement around the frontal horns bilaterally (thick white arrows).

On brain MRI, the infantile AD form is characterized by the following diagnostic criteria: (i) extensive cerebral white matter changes with frontal predominance, (ii) periventricular rim with high T1 and low T2 signal intensities, (iii) abnormal signal of basal ganglia and thalami, (iv) brainstem abnormalities, particularly involving the medulla and midbrain, and (v) contrast enhancement in multiple regions that is thought to be related to accumulation of Rosenthal fibers secondary to dysfunction of astrocytic endfeet (Figure 5) (83). Juvenile or adult forms have a remarkably different MR pattern, with predominant involvement of the lower brainstem (84).

##### Canavan Disease

Canavan Disease (CD) is an autosomal recessive disorder (MIM# 608034) caused by mutations in the aspartoacylase (*ASPA*) gene, encoding the ASPA enzyme that hydrolyzes N-acetyl-L-aspartic acid (NAA) to aspartate and acetate in oligodendrocytes. Consequently, defective N-acetylaspartate catabolism reduces levels of brain acetate that is crucial for myelin lipid synthesis and leads to accumulation of NAA, resulting in spongiform degeneration of cerebral white matter (85). After an initial normal development within the first months of life, children with CD experience developmental regression with progressive spasticity, seizures, vision impairment, and pseudobulbar signs (86). Macrocephaly is almost always present within the first year of life. Prognosis is poor with death usually occurring before age of 10.

**Figure 6:**
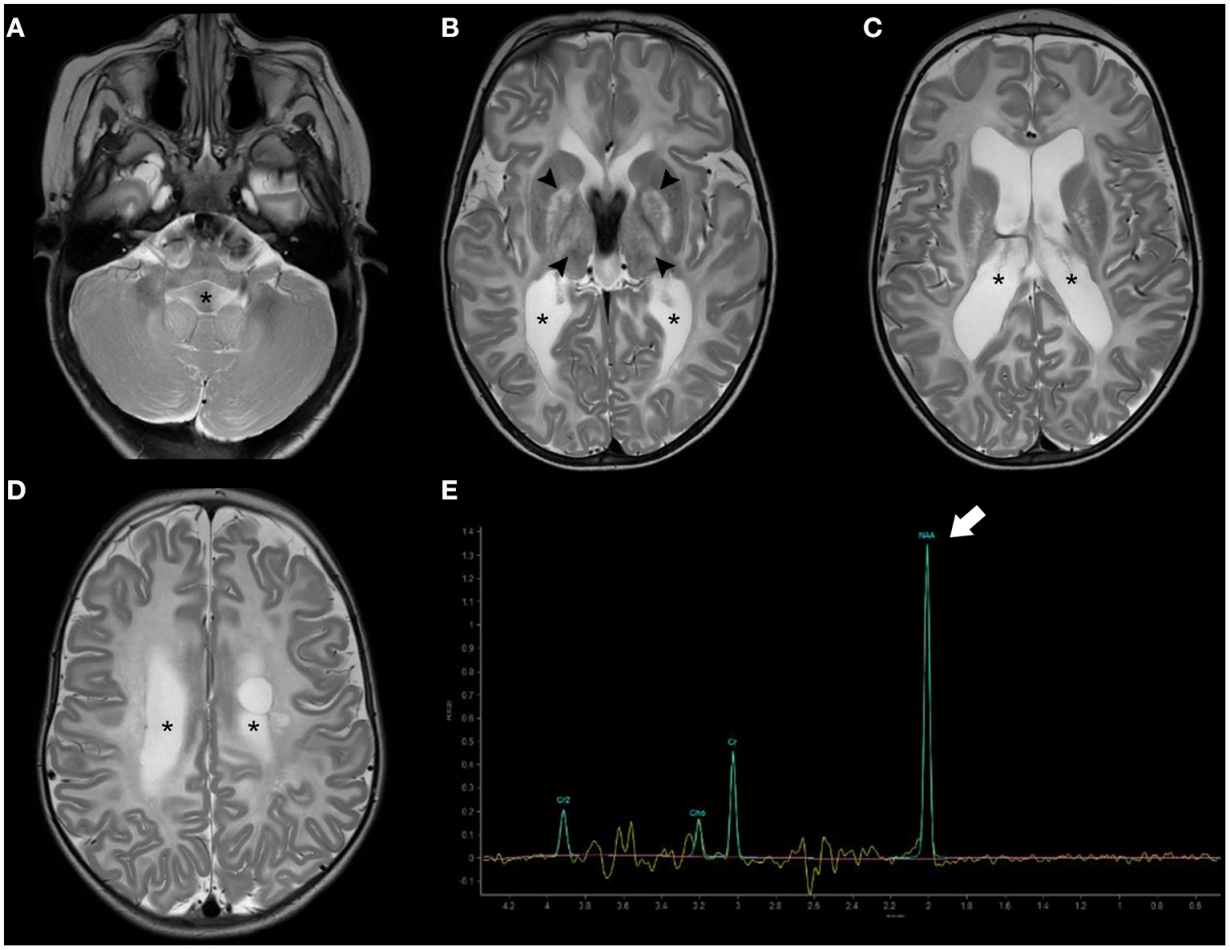
Brain MRI findings of Canavan disease. Axial T2WI **(A–D)** of a 2.3-years-old female infant with Canavan disease demonstrate diffuse, bilateral, and symmetric supratentorial and infratentorial white-matter hyperintensity associated with enlargement of the ventricular system (asterisks). There is also hyperintensity of the central gray-matter, mainly the globus pallidus and thalami bilaterally (black arrowheads). MR proton spectroscopy (intermediate TE) **(E)** depict increased NAA peak (thick white arrow) relatively to the other peaks, a feature near pathognomonic of this disease.

Brain MRI shows bilateral symmetric T2 white matter hyperintensity involving predominantly the subcortical white matter including subcortical U-fibers/arcuate fibers, as well as variable involvement of the basal ganglia/thalami and cerebellar white matter (87). Importantly, MR spectroscopy is almost pathognomonic of this disease, showing an elevated NAA peak (88). As the condition progresses, the white matter abnormalities progress centripetally and brain atrophy ensues, with progressive enlargement of the ventricular system (Figure 6).

##### Megalencephalic Leukoencephalopathy With Subcortical Cysts

**Figure 7:**
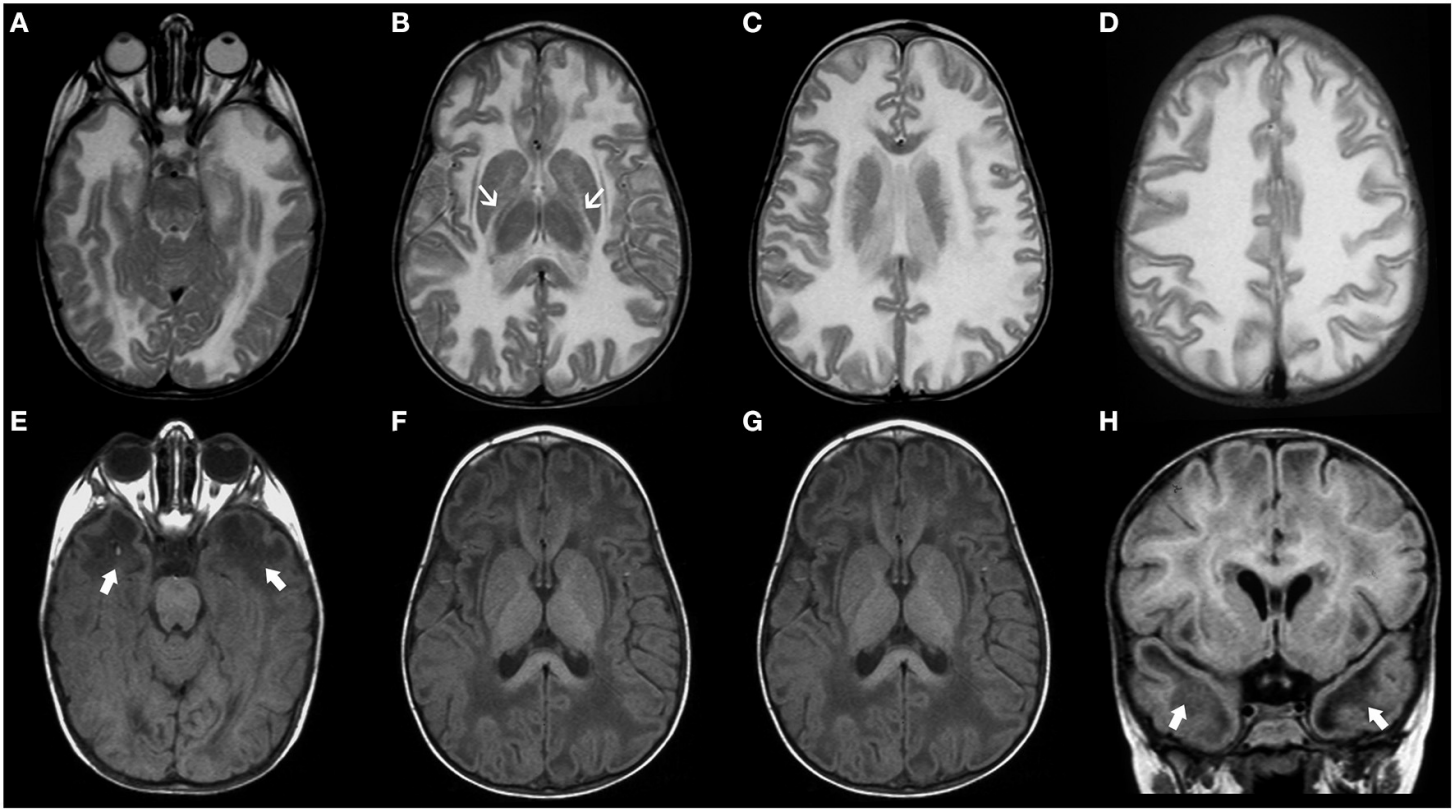
Imaging characteristics of Megalencephalic Leukoencephalopathy With Subcortical Cysts (classic form). Axial T2WI **(A–D)**, axial **(E,F)**, and coronal **(G)** T1WI, and coronal FLAIR **(H)** performed in an affected boy at 1.8 years of age show diffuse, bilateral, and symmetric cerebral white matter hyperintensity including the subcortical white-matter/U-fibers, leading to brain swelling and effacement of the cortical sulci. There is a characteristic double-line signal abnormality in the posterior limb of the internal capsule (white arrows), i.e., a residual central dark line surrounded by two strands of hyperintensity in this location. Also note the typical areas of cystic-like white-matter rarefaction in the temporal-polar regions on T1WI and FLAIR (thick white arrows).

Megalencephalic leukoencephalopathy with subcortical cysts (MLC) is a neurodevelopmental disorder caused by biallelic mutations in the *MLC1* gene in two-third of cases (MLC1) (89). It may also be due to biallelic (MCL2A) or more rarely monoallelic variants (MCL2B) in *HEPACAM*, encoding GlialCAM which is an MLC1-interacting protein in junctions between astrocytes (90). The typical presentation of MLC includes macrocephaly (between +4 and 6 SD) during the first year of life in a child with mild DD who develops gradual motor deterioration with ataxia and spasticity (60). However, the clinical course is not as severe as the one observed in Alexander and Canavan disease. Seizures are common whereas cognition is usually preserved. The neuroradiological hallmarks include diffuse, confluent areas of white matter signal abnormality with relative sparing of the corpus callosum and associated brain swelling as well as subcortical cysts and/or areas of near-cyst white matter rarefaction predominantly located in the anterior temporal and frontopolar regions (Figure 7). White matter edema decreases with time leading to atrophic changes. Clinical presentation and neuroradiological findings in MLC1 and MCL2A (classic MLC) overlap, with a common progressive course. In addition, affected patients usually demonstrate a double-line signal abnormality in the posterior limb of the internal capsule as well as cerebellar involvement. On the other hand, MCL2B shows a milder phenotype with preservation of the motor function as well as sparing of the cerebellar white-matter and posterior limb of the internal capsule. Moreover, it usually presents neuroradiological signs of stability or improvement over years (91).

##### Childhood Ataxia With Central Hypomyelination/Vanishing White Matter Disease (CACH/VWMD)

Vanishing white matter disease (VWMD; MIM# 603896) is an autosomal recessive leukoencephalopathy belonging to the group of astrocytopathies that is caused by mutations in any of the five genes (*EIF2B1, EIF2B2, EIF2B3, EIF2B4*, and *EIF2B5*) encoding the subunits of eukaryotic translation initiation factor 2B (eIF2B) that is essential for protein synthesis (92, 93). Genotype–phenotype correlation has been outlined with a phenotypic spectrum ranging from the antenatal or early infantile-onset with the poor outcome to adult-onset with slow progression (91). Affected individuals typically have normal early development, followed by neurological deterioration triggered by stress-provoked episodes (minor head trauma or febrile infections) of rapid decline. Head circumference is usually normal; however, severe progressive macrocephaly occurring after the age of 2 years has been reported (91, 93). Neurological symptoms may include ataxia, spasticity, seizures, cognitive or psychiatric problems, the latter being especially common in adolescence and adult-onset. Clinical severity is usually inversely related to the age of onset. In addition, irrespective of their age, females with VWMD are also often affected by primary or premature ovarian failure (ovarioleukodistrophy) (94).

**Figure 8:**
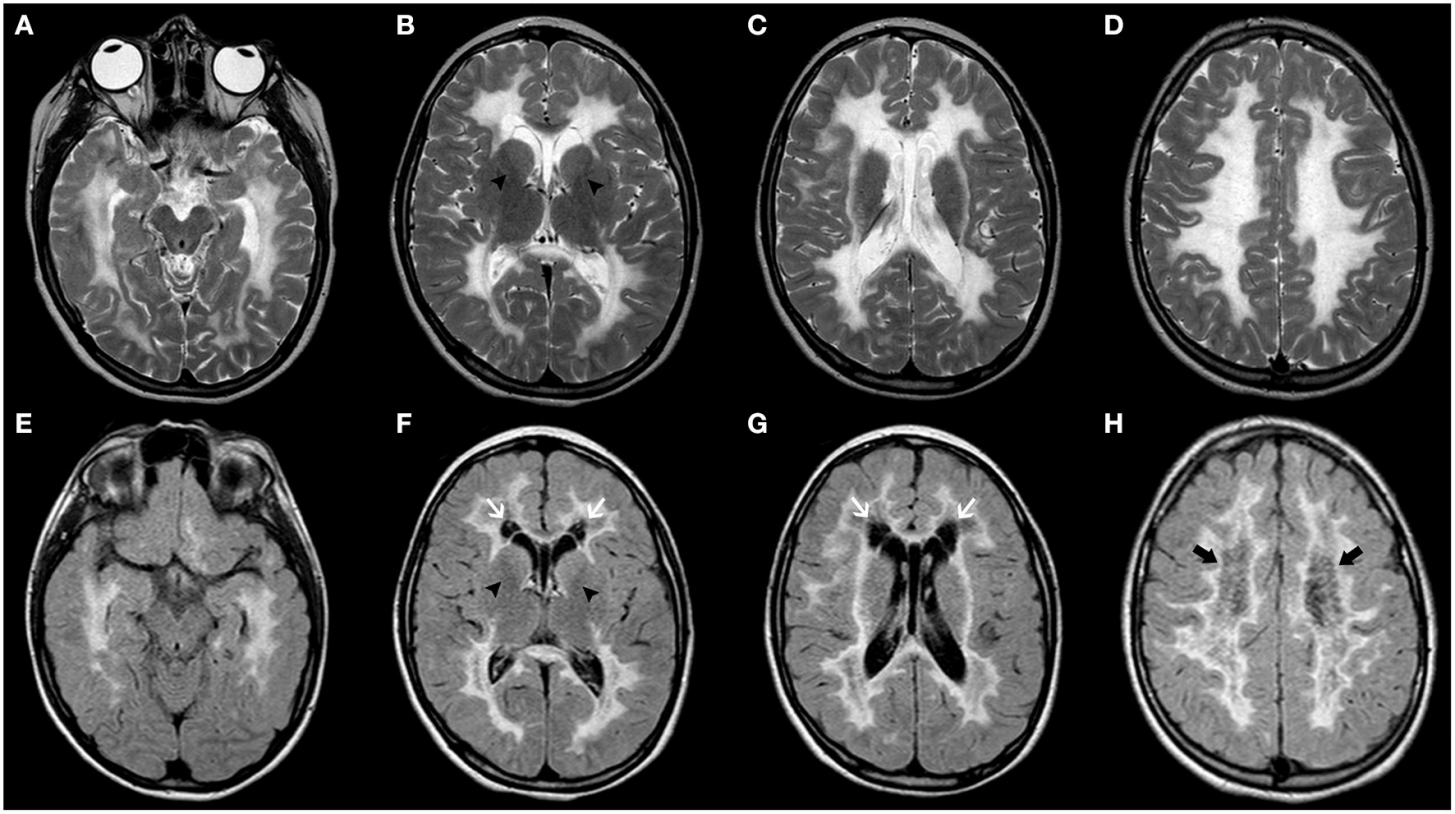
Imaging characteristics of Childhood Ataxia with Central Hypomyelination/Vanishing White Matter Disease (CACH/VWMD). Axial T2WI **(A–D)** and FLAIR **(E–H)** of an 11-year-old boy with CACH/VWMD demonstrates bilateral, confluent, symmetric periventricular, and deep white matter hyperintensity, with relative sparing of the anterior limb of the internal capsule (black arrowheads) and the subcortical white-matter/U-fibers. There are areas of cystic degeneration around the frontal horns (white arrows) and in the centrum semi-oval bilaterally (thick black arrows), with some radiation stripes depicted within the latter location.

Vanishing white matter disease, especially the classic early-infantile form, is usually a well-recognizable disorder, characterized by diffuse and symmetric T2WI/FLAIR hyperintensities involving mainly the deep and sub-cortical white matter with progressive rarefaction and cystic degeneration, with corresponding increased diffusion and eventually a parenchymal CSF-like signal (Figure 8). Radiating stripes may be detected inside the cystic areas on T1WI, FLAIR, and PD. The cerebellar white matter and the central tegmental tracts may also be involved, while the outer rim of the corpus callosum, the anterior branch of the internal capsule, and the anterior commissure are usually spared. Proton spectroscopy reveals progressive disappearance of the major metabolites, replaced by lactate and glucose (92, 95, 96).

### Fragile X Syndrome

Fragile X syndrome (OMIM# 300624, FXS) has been traditionally considered the most common genetic cause of ID and ASD in males with a prevalence ranging from 1/3,717 to 1/8,918 (97).

It is most often caused by a trinucleotide expansion (CGG >200, also called a full mutation) in the 5'-untranslated region of the *FMR1* gene, resulting in transcriptional silencing of the FMR1 promoter with a consequent loss of its product, the Fragile X mental retardation 1 protein (FMRP). Other genetic mechanisms including intragenic deletions/duplications and single-nucleotide variants are responsible for <1% of the molecular diagnoses of FXS. Allelic disorders with a premutation-sized repeat (55–200 CGG repeats) are Fragile X-associated tremor/ataxia syndrome (FXTAS) and Fragile X-associated primary ovarian insufficiency (FXPOI) (98).

Fragile X mental retardation 1 protein has a central role in gene expression and regulates the translation of several mRNAs (99), many of which are involved in the development and maintenance of synaptic connectivity (100, 101). These observations are in line with the synaptic dysfunction found in mouse models of FXS that recapitulate the behavioral features, including ASD, observed in the majority of FXS cases (102).

Clinically, macrocephaly and other craniofacial features (long face, prominent forehead and chin, high-arched palate, and large protruding ears) are found in about 80% of cases (103). Fragile X syndrome is indeed a clinically recognized syndrome in over 90% of subjects with positive FXS testing (104). However, these features may be absent during early childhood and become more evident over age. Similarly, macroorchidism and connective tissue involvement, including mitral valve prolapse, aortic root dilatation, joint hypermobility, and soft velvety skin, become more evident in post-pubertal males. Epilepsy has been recorded in 15–20% of cases and no specific brain anomalies have been reported in up to 50% of cases with neurological comorbidities (103).

Notably, FXS testing has been considered a first diagnostic-tier in patients with ID/ASD, according to the recommendations by the American College of Medical Genetics and Genomics and the American Academy of Pediatrics (105) before the advent of the recent genetic technologies. Yet, the identification of several novel ID genes and the large use of chromosomal microarray and NGS technologies in recent years have moved FXS testing to a second diagnostic-tier investigation (106, 107). Contrarily to FXTAS, Fragile X syndrome does not present suggestive features on neuroimaging.

### Macrocephaly in Skeletal Dysplasias

Macrocephaly is observed in a wide range of skeletal dysplasias, reflecting variation in the density, size, and shape of the skull. First, it is important to recognize treatable conditions, like vitamin D deficiency rickets (108). Softening of the skull (craniotabes), frontal bossing, and delayed closure of fontanelles are typical skull defects in an infant with failure to thrive, delayed walking, restlessness, lack of sleep, and other clinical signs including enlargement of the ends of the ribs (“rachitic rosary”), bowed legs, knock knees, thickened wrist, and ankles (109, 110). Another treatable condition is Beta-thalassemia (111) in which macrocephaly is attributable to extracranial hematopoiesis in the skull bones.

Among the several types of skeletal dysplasias, Achondroplasia is the most common cause of disproportionate short stature, and it is constantly associated with macrocephaly. Affected individuals have rhizomelic shortening of the limbs, macrocephaly, and characteristic facial features with frontal bossing and midface retrusion (112). In addition to a disproportionate neurocranium, macrocephaly could be also related to a small foramen magnum that impedes CSF drainage, resulting in ventriculomegaly and possible injury of the bulbomedullary junction. Further common neuroimaging findings are bilateral deep transverse temporal sulci, incomplete hippocampal rotation, oversulcation of the mesial temporal lobe, loss of gray-white matter differentiation of the mesial temporal lobe, and a triangular shape of the temporal horn (113). Typical skeletal abnormalities include wide ribs, square iliac bones, a “champagne glass”—shaped pelvic inlet, short and robust tubular bones and proximal femoral radiolucency, short pedicles, narrowing of the lumbar interpediculate distances, and spinal stenosis. It is a clinically recognizable disorder due to a gain of function mutation (c.1138G>A, p.Gly380Arg) in *FGFR3 (*MIM# 100800). Other mutations in the same gene, with the recurrence of c.1620C>A and c.1620C>G, both resulting in the p.Asn540Lys substitution, result in a milder phenotype, namely Hypocondroplasia (MIM# 146000) (114).

Macrocephaly may be also found in osteopetrosis (i.e., an increase in calvarial density), a clinically and genetically heterogenous condition characterized by increased bone mass owing a defect in osteoclast function or formation. Several causative genes have been identified to date, being half of the cases explained by biallelic variants in *TCIRG1* (115, 116).

Progressive thickening of the craniofacial bones is also observed in craniometaphyseal dysplasia, a genetic craniotubular bone disorder characterized by early progressive hyperostosis and sclerosis of the craniofacial bones, and abnormal modeling of the metaphyses of the tubular bones (117). Craniometaphyseal dysplasia has been linked to heterozygous mutations in *ANKH* (MIM# 123000) (118) and biallelic mutations in *GJA1 (*MIM# 218400) (119). Another sclerosing bone dysplasia is the Osteopathia striata with cranial sclerosis, an X-linked dominant disorder due to mutation in *AMER1* (MIM# 300373). It is a recognizable condition for the presence of longitudinal striations visible on radiographs of the long bones, pelvis, and scapulae (120). In males, the disorder is usually associated with fetal or neonatal lethality. Craniodiaphyseal dysplasia is a further skeletal dysplasia characterized by generalized hyperostosis and sclerosis, especially involving the skull and facial bones, due to heterogenous mutations in *SOST* gene (MIM# 122860) (121). The association of macrocephaly with constricted thoracic cage and short ribs may suggest two types of Short-rib thoracic dysplasia, due to biallelic variants in *IFT81* (MIM# 617895) (122) or *WDR60* gene (MIM# 615503) (123).

Absent vertebral body ossification and macrocephaly are found in Spondylo-megaepiphyseal-metaphyseal dysplasia, associated with biallelic variants in *NKX3-2* (MIM# 613330) (124). Bone fragility, craniosynostosis, ocular proptosis, hydrocephalus, and distinctive facial features including frontal bossing, midface hypoplasia, and micrognathia are a typical hallmark of Cole-Carpenter syndrome-2 (CLCRP2) (MIM# 616294), due to biallelic variants in *SEC24D* (125). Another clinical recognizable disorder is Robinow syndrome, characterized by distinctive facial features (hypertelorism, midface hypoplasia, large nasal bridge, short upturned nose, and anteverted nares), genital anomalies, mesomelic limb shortening, and brachydactyly (126). Vertebral segmentation defects and ribs fusion occur in the recessive form linked to ROR2 mutations (MIM# 268310) (127) whereas umbilical hernia and supernumerary teeth are more common in the autosomal dominant forms due to WNT5A (MIM# 180700), DVL1 (MIM# 616331), DVL3 (MIM# 616894) mutations (128).

### Macrocephaly Associated With Neurocutaneous Syndromes

#### Neurofibromatosis Type 1

Neurofibromatosis type I (NF1), also called von Recklinghausen disease, belongs to the group of neuro-oculo-cutaneous disorders, collectively known as Phakomatoses. It is one of the most common inherited genetic conditions with an incidence approximately of 1 in 3,000 (129). It is caused by mutations in *NF1*, encoding neurofibromin which functions as a tumor suppressor and negative growth regulator by inhibiting the Ras/MAPK signaling pathway. Traditionally, a clinical diagnosis is established in the presence of any two of the following criteria: (1) cafe-au-lait spots; (2) axillary or inguinal freckling; (3) Lisch nodules; (4) neurofibromas/plexiform neurofibroma; 5) optic pathway gliomas (OPG); (6) distinctive osseous lesion such as sphenoid dysplasia and pseudoarthrosis; (7) first degree relative with NF1 (130). Since not all these features are clinically present during the first years of life, molecular genetic testing is recommended in children even though they fulfill only pigmentary features of NF1 (131). Moreover, subtle clinical presentations and significant intrafamilial variability have been largely reported (132), raising concerns regarding the need to revisit the diagnostic criteria (133). Although they remain unchanged, the diagnosis and management of NF1 have been recently updated (129).

**Figure 9:**
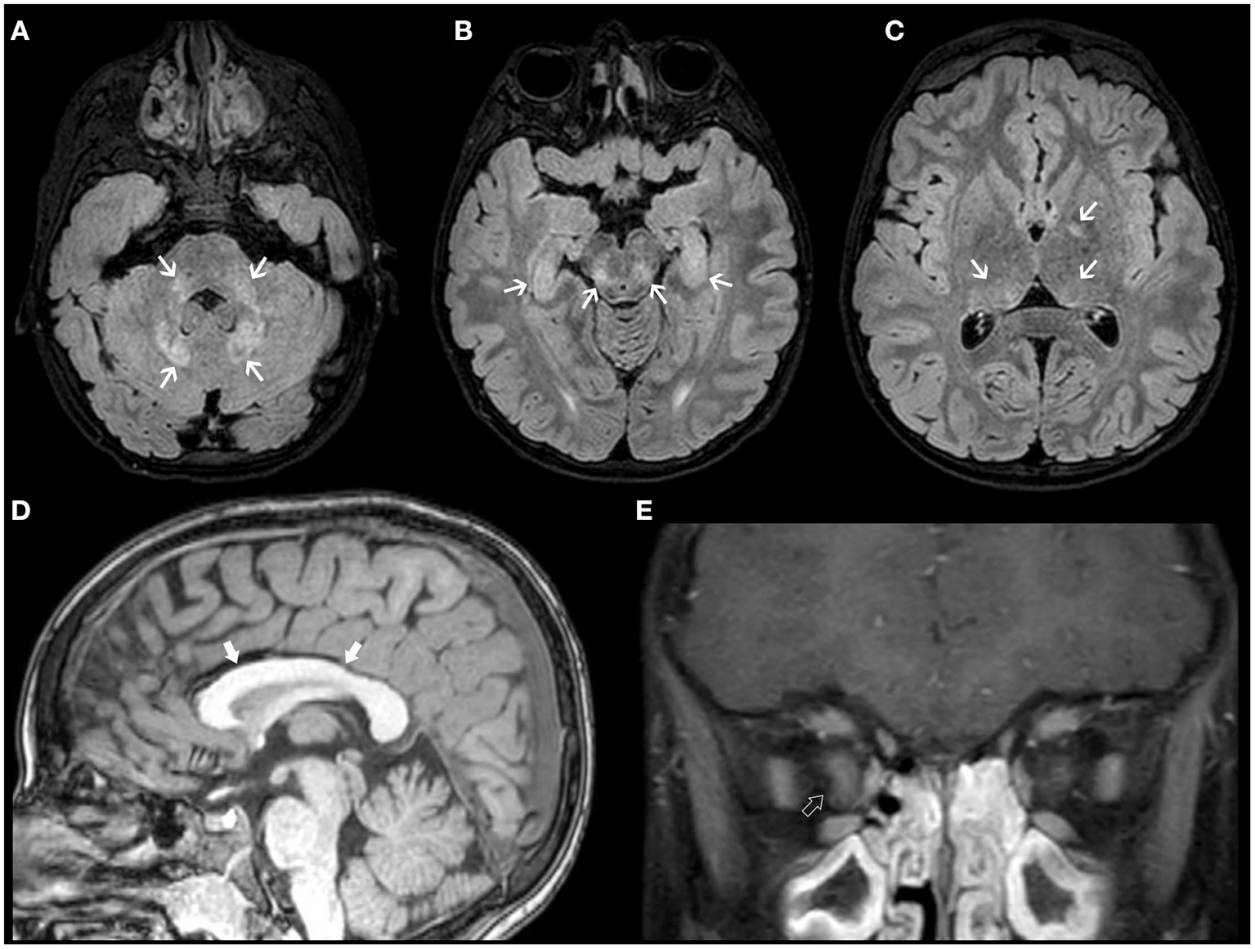
Imaging characteristics of Neurofibromatosis Type 1 (NF1). Axial FLAIR **(A–C)** and sagittal T1 WI **(D)** of an 18-year-old boy with NF1 reveals multiple focal areas of abnormal hyperintensity (FASI) (white arrows) distributed in the dentate nuclei, middle cerebellar peduncles, mesencephalic tegment, hippocampi, and pulvinar bilaterally as well as left globus pallidus. There is also diffuse thickening of the corpus callosum (thick white arrows). Coronal contrast-enhanced T1WI FAT SAT of the orbits **(E)** of the same patient show thickening, tortuosity, and enhancement of the intra-orbital segment of the right optic nerve (open arrow), in keeping with an ipsilateral optic nerve glioma.

Recently, an association between NF1 and neurodevelopmental abnormalities has been increasingly acknowledged (134). Macrocephaly is observed in about 35–45% of NF1 cases and is thought to be related to a dysregulation of growth process driven by abnormal Ras/MAPK signaling pathway leading more commonly to diffuse, symmetric megalencephaly with a thick corpus callosum (135). In the vast majority of cases, the cortex remains unremarkable at visual inspection. Rarely, MCD have been reported in NF1, including unilateral MEG/HMEG and also polymicrogyria (PMG) (136–138). Additional neuroimaging *stigmata* of NF1 include the typical unidentified bright objects (UBOs) in the basal ganglia, internal capsule, brainstem, and cerebellum that usually appear by 3 years of age and regress spontaneously in adolescence (Figure 9) (135). Subjects with NF1 have an increased oncogenic risk, including juvenile myelomonocytic leukemia, rhabdomyosarcoma, malignant peripheral nerve sheath tumor and non-invasive pilocytic astrocytoma, particularly OPG (131). The latter represents the major management challenge since clinical assessment for OPG is advised every 6–12 months until 8 years, but routine MRI assessment is not recommended in asymptomatic NF1 individuals and no signs of clinical visual pathway disturbance.

Neurofibromatosis type 1 has an overlap clinical phenotype with Legius syndrome (MIM# 611431), originally termed “neurofibromatosis type 1-like syndrome” and caused by SPRED1 mutations. Affected subjects frequently fulfill the diagnostic criteria for NF1 based on pigmentary manifestations of café-au-lait spots and distinctive freckling patterns and may also present with macrocephaly (139).

#### Tuberous Sclerosis Complex

Tuberous sclerosis complex (TSC), formerly known as Bourneville Disease, is an autosomal dominant neurocutaneous disorder due to mutations in *TSC1* or *TSC2*, two tumor suppressor genes belonging to the GATOR1 complex that negatively regulates mTOR-PIK3CA pathway (140). Affected patients usually present abnormalities of the skin, brain, kidney, heart, and lungs (141, 142).

**Figure 10:**
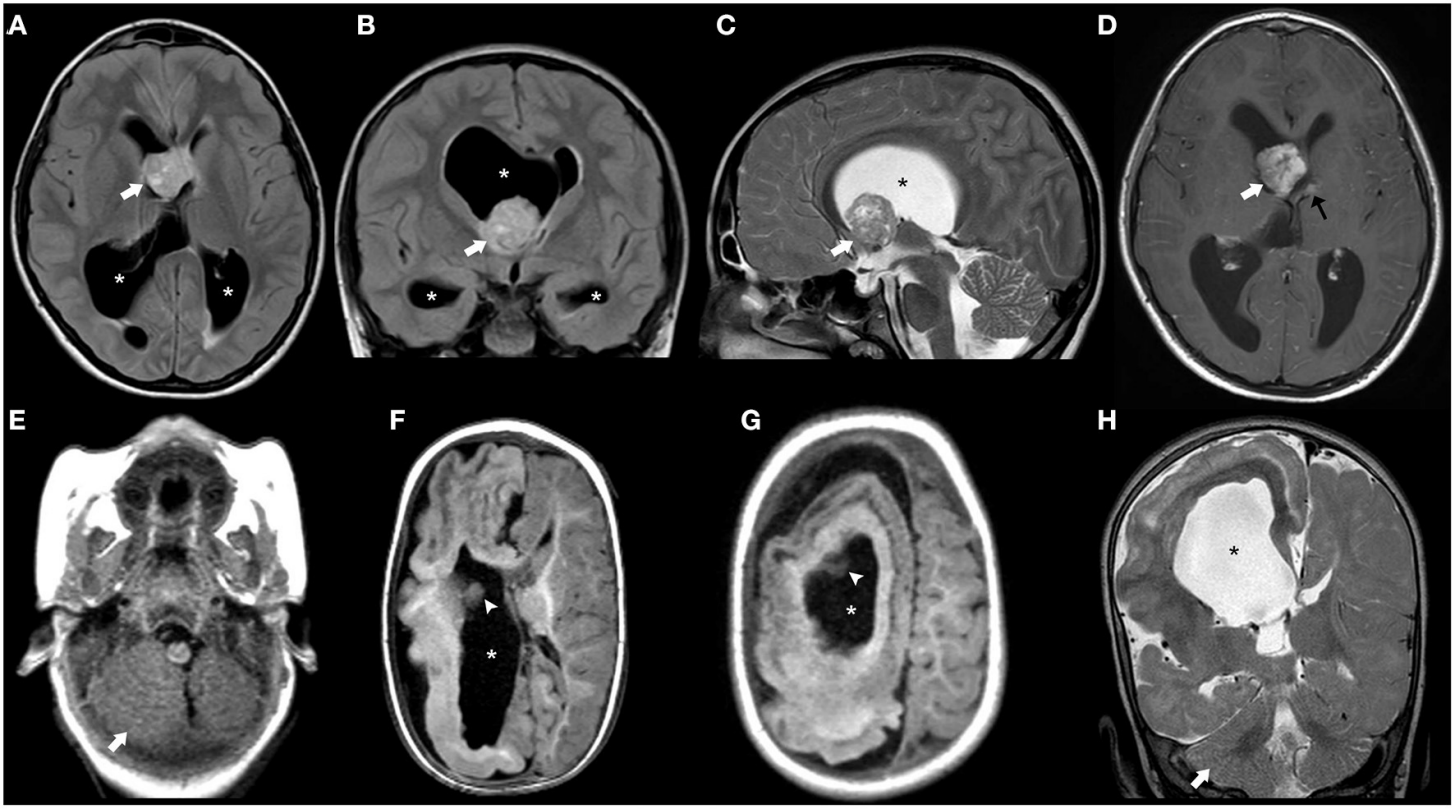
Imaging characteristics of Tuberous Sclerosis (TS). Axial **(A)** and coronal **(B)** FLAIR images, sagittal T2WI **(C)** and axial post-gadolinium TWI **(D)** of a 14-years-old boy with TS demonstrate a contrast-enhanced mass larger than 10 mm in the region of the right foramen of Monro (thick white arrow) and with progressive growth in comparison with previous studies (not shown), compatible with a giant cell astrocytoma. There is the contralateral deviation of the septum pellucidum and asymmetric dilatation of the ventricular system (asterisks) including the temporal horns with incipient signs of periventricular interstitial edema and effacement of the cortical sulci in keeping with decompensated hydrocephalus. Also note a small contralateral, contrast-enhancing subependymal nodule (black arrow). Axial T1WI **(E–G)** and coronal T2WI **(H)** in another patient with TS at the age of 11 years reveals right hemimegalencephaly associated with marked abnormal cortex and white matter as well as enlargement of the ipsilateral ventricular system (asterisks). Also, note small subependymal nodules (white arrowheads) as well as enlargement of the right cerebellar hemisphere (thick white arrows).

CNS involvement is seen in most patients with TSC; cardinal neurologic manifestations include supratentorial cortical tubers, corresponding on histology to FCD type IIb, radial migration lines, subependymal nodules, subependymal giant cell astrocytomas as well as white-matter cysts and cerebellar tubers (143, 144). Macrocephaly may be due to both MEG/HMEG (145–149) and obstructive hydrocephalus related to subependymal giant cell astrocytoma. Imaging findings of cortical tubers are similar to those of FCD type IIb (see related paragraph), although with possible associated calcifications. Similar to FCD, the imaging characteristics of cortical tubers change over time, being more visible in the neonatal period (when they are hypointense on T2 and hyperintense on T1-weighed images), likely reflecting rapid changes of myelination in epileptogenic areas (Figure 10) (150). Of note, cyst-like cortical tubers have been strongly associated with TSC2 gene mutations and a more aggressive seizure phenotype (151). Cerebellar tubers are also more commonly seen in the context of TSC2 and typically present peculiar imaging characteristics due to the frequent association with calcification, contrast enhancement and slight atrophy of the involved cerebellar region with associated folial retraction (152–155). Specific guidelines for diagnosis, surveillance, and management have been outlined by the International Tuberous Sclerosis Complex Consensus Group (143).

#### Gorlin Syndrome (Nevoid Basal Cell Carcinoma Syndrome)

Gorlin syndrome (GS), also known as nevoid basal cell carcinoma syndrome, is a neurocutaneous disorder linked to heterozygous mutations in *PTCH1* (60–85%) and *SUFU* (<10%), two important genes of the Sonic Hedgehog signaling pathway (156). To date, the association of *PTCH2* variants with GS remains controversial (157, 158).

It is a clinically recognizable disorder characterized by skin abnormalities, jaw keratocysts and skeletal anomalies that should be suspected in the presence of specific diagnostic criteria (159). Macrocephaly is commonly found during the first months of life, secondary to diffuse symmetric megalencephaly and associated with frontal bossing and hypertelorism. The ventricles are also slightly larger while the corpus callosum may be hypoplastic (160). Jaw keratocysts and ectopic calcifications, particularly in the falx (sheet-like), and bridging of the sella turcica are typical hallmarks that become evident during the second decade of life. Other typical features include facial milia, palmar/plantar pits, cardiac, and ovarian fibromas and predisposition to various tumors including basal cell carcinoma and medulloblastoma. Medulloblastomas associated with GS occur at a very young age (up to 3 years), belong to the Sonic Hedgehog group, and usually present a desmoplastic or classic phenotype on histology (161). The risk of developing medulloblastoma is substantially higher in individuals with SUFU mutations (33%) than in those with PTCH1/2 pathogenic variants (<2%). Conversely, PTCH1 mutations present a stronger association with odontogenic keratocysts (162, 163). Spinal vertebral anomalies such as hemivertebrae, fusion or elongation of the vertebral bodies, and cleft lip/palate can also be detected in some cases (164–166).

### Macrocephaly in Overgrowth Syndromes

Overgrowth syndromes comprise a heterogeneous group of diseases that are characterized by excessive tissue development, which may be generalized or segmental. Despite the lack of a formal diagnosis, the term overall overgrowth usually refers to a disorder displaying height and head circumference >+2 SD, target height (calculated based on mid-parental height) as well above +2 SD, with or without associated dysmorphic features (167). Another term often used as synonymous is macrosomia, defined as weight and length/height >97 centile. On the other hand, regional segmental overgrowth is excessive growth compared to an equivalent body part or age-related peer group (168).

Some important considerations should be kept in mind when facing overgrowth syndromes. First, they should be distinguished by several non-genetic causes of overgrowth that include (i) familial trait, (ii) endocrine conditions (e.g., precocious puberty, hyperthyroidism, congenital adrenal hyperplasia, growth hormone-secreting adenoma, familial glucocorticoid deficiency, and aromatase deficiency), and (iii) over-nutrition conditions including newborn/infants of diabetic mothers (169). Conversely, the presence of DD/ID, congenital malformations, abdominal wall defects, organomegaly, and dysmorphic features should alert pediatricians for a possible genetic etiology. Of note, neonatal hypoglycemia could be found in both acquired causes (e.g., newborn of a mother with gestational diabetes) and genetic syndromes [e.g., Beckwith-Wiedemann syndrome (BWS) and some mTOR-related disorders]. Depicting the natural history of growth in charts is extremely important to recognize a specific instance of growth acceleration (mostly from acquired causes such as precocious puberty) and it may also help to recognize specific growth patterns of syndromes, such as Cantu and Sotos syndromes. Finally, disorders with associated oncogenic risk should be promptly recognized to adopt accurate surveillance (170). Among overgrowth syndromes, overall overgrowth related to germline mutations has to be distinguished from segmental overgrowth, mostly due to somatic mutations in genes of the PI3K-AKT-mTOR pathway.

#### (Segmental) Overgrowth Syndromes With Vascular/Skin Features

##### Syndromes Caused by Abnormalities in the PI3K-AKT-mTOR Pathway

Mutations in several core components of the PI3K-AKT-mTOR pathway have been recently recognized to cause a broad disease spectrum. The PI3K-AKT-mTOR pathway is well known to play a crucial role in cell growth, maturation, proliferation, and energy metabolism (171). Both gain-of-function variants in activator genes (such as *MTOR, AKT3*, and *PIK3CA*) (172, 173) and loss-of-function variants in negative regulator genes (such as *TSC1* and *TSC2*) (174) lead to upregulation of mTOR signaling, resulting in cellular overgrowth and abnormal migration. Collectively, these disorders not only overlap molecularly, but also share clinical, neuroimaging, and neuropathologic features that are clinically recognizable in most affected individuals.

Mutations in activator genes may be germline (i.e., constitutional) or, more commonly, somatic (i.e., post-zygotic), depending on the developmental stage of brain development at which the mutation occurs. This leads to a phenotypic spectrum including focal cortical dysplasia (175), HME, MEG (172, 173), and segmental body overgrowth (12, 176–178). In addition, there are commonly vascular and skin features that should orient clinicians toward this diagnosis.

**Figure 11:**
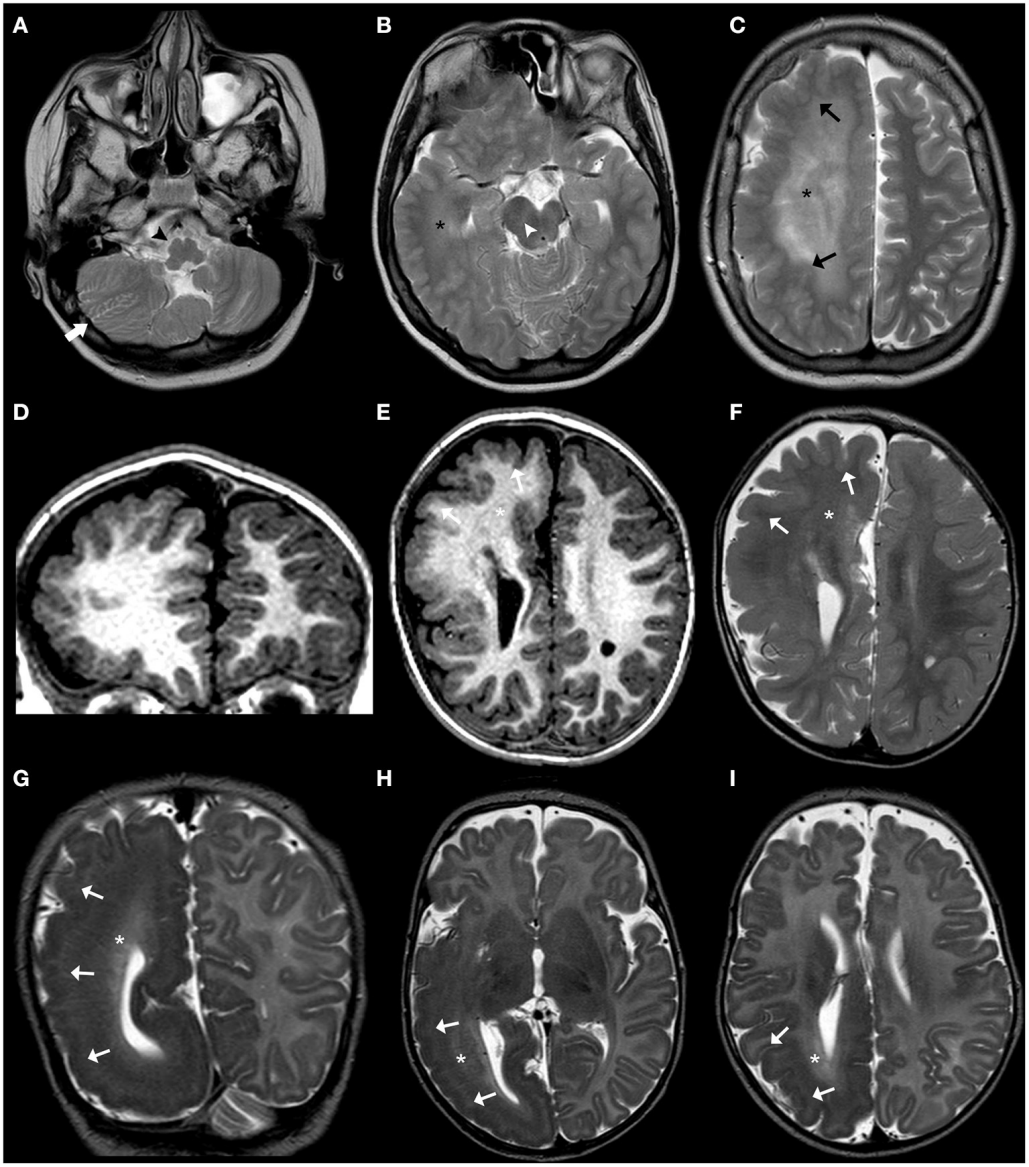
Imaging characteristics of brain overgrowth disorders (continuation). Axial T2WI **(A–C)** of a 16-years-old boy demonstrate right unilateral megalencephaly/hemimegalencephaly with abnormal cortex (black arrows) and abnormal white-matter signal intensity (asterisks). There is also enlargement of the right mesencephalon (white arrowhead) and medulla oblongata (black arrowhead) as well as enlarged and dysplastic right cerebellar hemisphere (thick white arrow). These findings were previously designated as “total hemimegalencephaly.” Coronal **(D)** and axial **(E)** T1WE and axial T2WI **(F)** of another child performed at 1.8 years of age show a right anterior form of unilateral megalencephaly/hemimegalencephaly with abnormal cortex (white arrowheads) and abnormal white-matter signal intensity (asterisks) within the affected region. Coronal **(G)** and axial **(H,I)** T2WI acquired in a third child at 2 months of age depict instead a right posterior form of unilateral megalencephaly/hemimegalencephaly, also with abnormal cortex (white arrowheads) and white-matter (asterisks) within the affected region, with enlargement of the ipsilateral occipital horn of the lateral ventricle.

**Figure 12:**
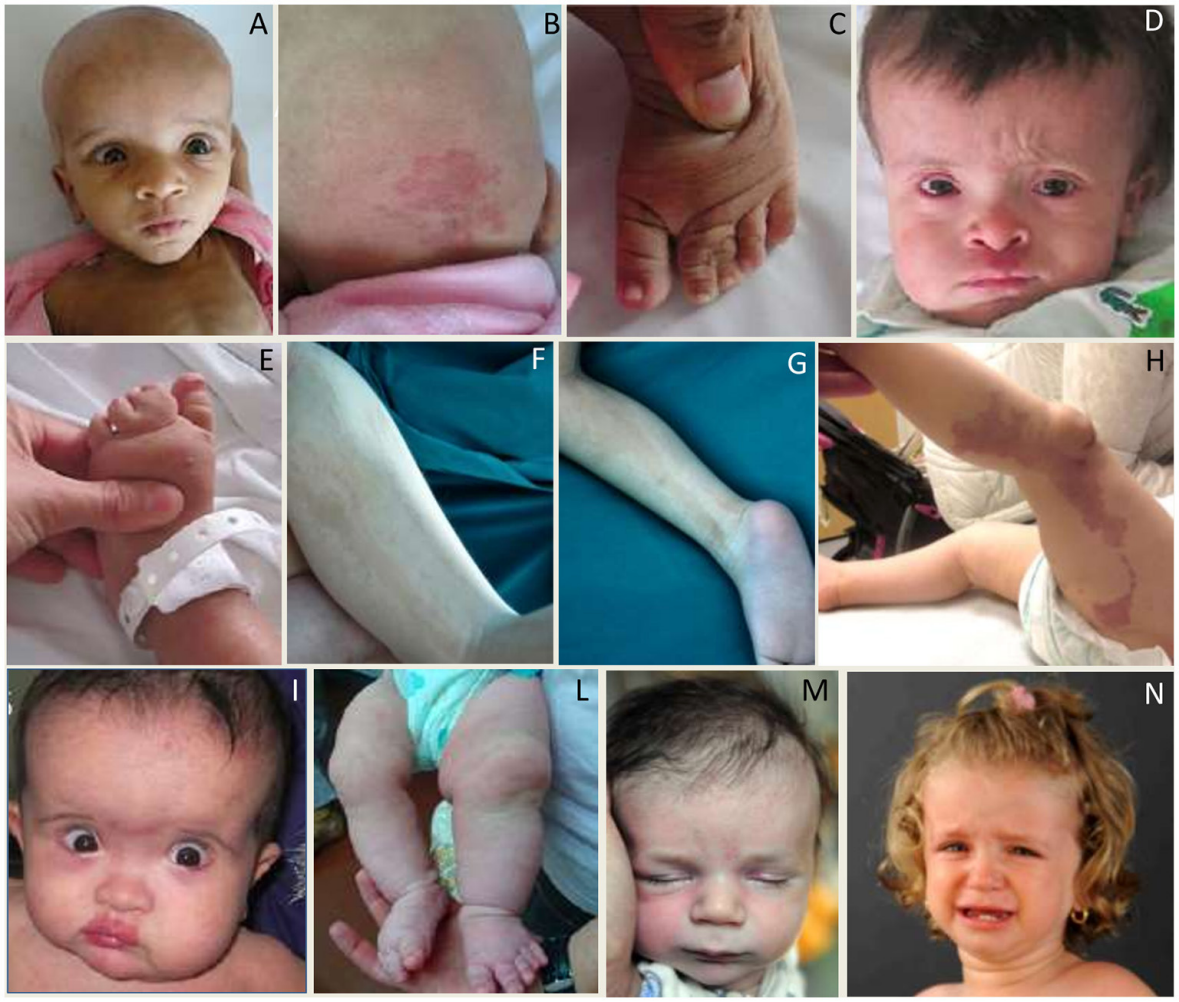
Photos of affected patients with either somatic or germline mutations in genes of the PI3K-AKT-mTOR pathway. **(A–C)** Photos of the face **(A)**, occipital region **(B)**, and left foot **(C)** of a subject with MCAP (somatic PIK3CA mutation p.Pro104Leu) showing MEG, occipital capillary malformation, and syndactyly of the second, third, and fourth toes. **(D,E)** Photos of the face **(D)** and left foot **(E)** of a subject with MCAP (somatic PIK3CA mutation p.Glu545Asp) showing MEG, capillary malformation of the philtrum, skin laxity of the forehead, and syndactyly of the second, third, and fourth toes. **(F,G)** Photos of lower limbs with pigmentary defects in a patient with Megalencephaly-Polymicrogyria-Pigmentary Mosaicism Syndrome (somatic MTOR mutation p.Thr1977Ile). **(H)** Diffuse vascular anomaly in the left lower leg in a subject with somatic mutation in PIK3CA (p.Gly914Arg). **(I–L)** Photos of the face **(I)** and lower extremities **(L)** of a subject with MCAP (somatic PIK3CA p.Met1043Ile) showing facial and body asymmetry, MEG with a prominent forehead, and capillary malformations on the face and body. **(M)** Photo of a subject with MEG, frontal bossing, nevus flammenus and retrognathia, harboring a germline variant in MTOR (p.Glu1799Lys). **(N)** Photos of a female with MEG and broad forehead harboring a germline variant in PIK3CA (p.Pro104Leu). Part of these photos are re-printed with permission from ref (182).

**Figure 13:**
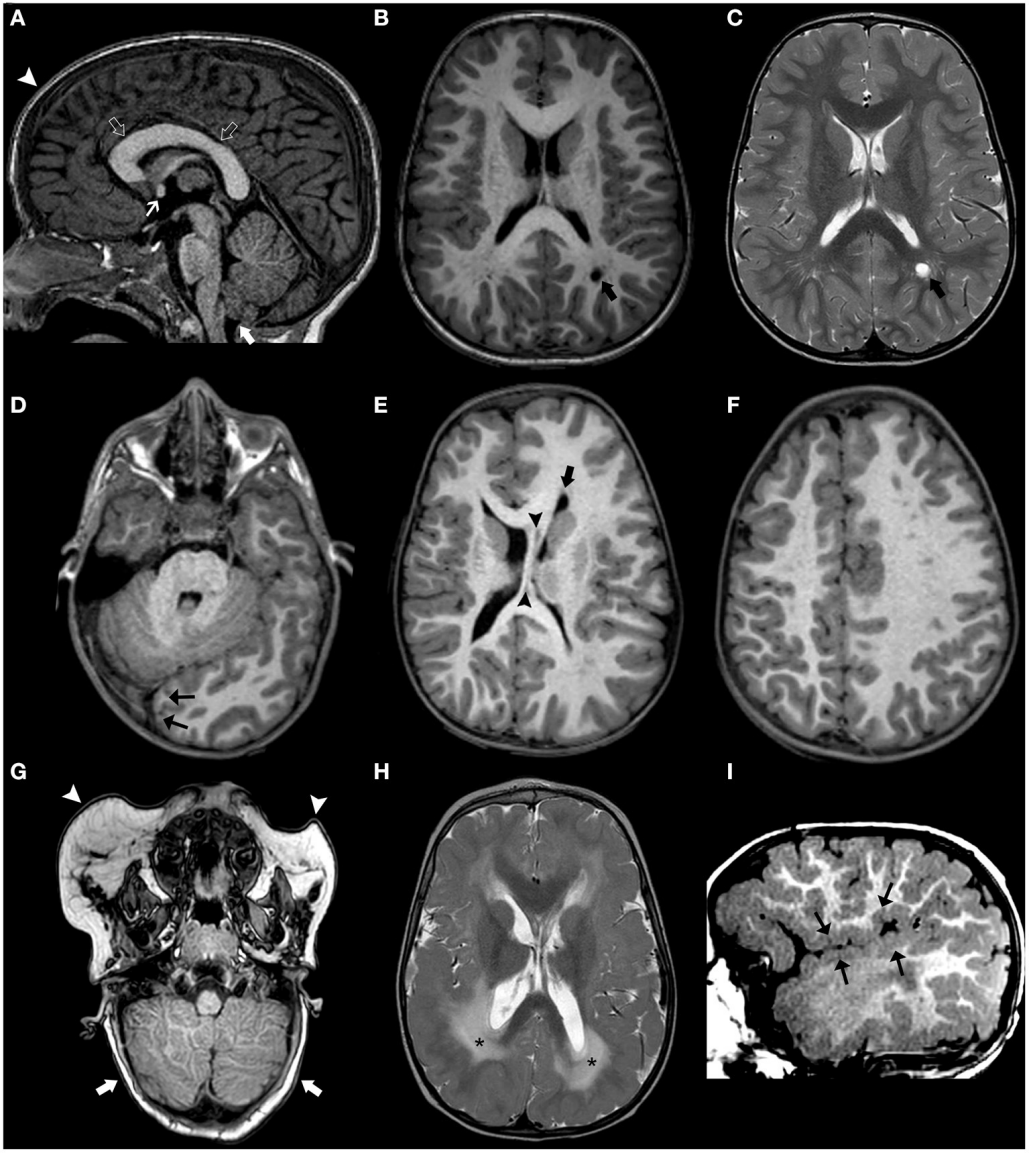
Imaging characteristics of brain overgrowth disorders. Sagittal **(A)** and axial **(B)** T1WI and axial T2WI **(C)** of a 3.5-years-old boy with macrocephaly due to a pathogenic PTEN mutation reveals signs of bilateral, symmetric megalencephaly with regular cortex and normal signal-intensity white matter. Note presence of frontal bossing (arrowhead), mega corpus callosum (open arrows), thickened anterior commissure (arrow), mild tonsillar herniation (thick white arrow), and peri-atrial dilated perivascular spaces, mainly on the left side (black thick arrow). Axial T1 WI **(D–F)** of a different patient with macrocephaly at 3.4 years of age show signs of unilateral megalencephaly/hemimegalencephaly with normal cortex and normal white matter. Note the “occipital sign” (black arrows) as well as anterior pointing of the left frontal horn (thick black arrow) and thickening of the midline structures (black arrowheads). Axial T1WI **(G)**, axial T2WI **(H)**, and sagittal T1WI **(I)** in a child with megalencephaly capillary syndrome performed at 1.6 years of age depict bilateral, asymmetric dysplastic megalencephaly with abnormal perisylvian polymicrogyric cortex (black arrows) and white matter signal changes (asterisks) as well as bilateral cerebellar dysplasia (thick white arrows). Also, note bilateral facial lipomatous lesions (white arrowheads).

Recently, a general genotype–phenotypic correlation has been outlined (179): mildly activating variants (that are typically germline) are associated with diffuse MEG with ID and/or ASD; moderately activating variants (typically high-level mosaic) are associated with MEG with pigmentary abnormalities of the skin; and strongly activating variants (usually very low-level mosaic) are associated with focal brain malformations including HME and focal cortical dysplasia (Figures 11–**13**).

##### PIK3CA-Related Overgrowth Syndromes

PIK3CA-related overgrowth syndromes (PROS) are an umbrella term that refers to a very large spectrum of conditions frequently associated with MEG/HMEG caused by a common genetic signature, namely gain of function mutations in *PIK3CA* gene leading to activation of the PI3K-AKT-mTOR pathway. Among the diverse PROS phenotypes there are the macrocephaly-capillary malformation-polymicrogyria syndrome (MCAP), hemihyperplasia-multiple lipomatosis, muscle hemihypertrophy, congenital facial infiltrating lipomatosis, epidermal nevi, isolated large lymphatic malformation, isolated macrodactyly, fibroadipose overgrowth, seborrheic keratosis, and benign lichenoid keratosis (12). More recently, it has been proposed to also include the Klippel-Trenaunay syndrome in this group (180, 181).

##### MCAP

Somatic mutations in *PIK3CA* account for about 90% of the Megalencephaly-capillary malformation syndrome (MCAP) cases reported so far (182), including subjects initially described as macrocephaly-cutis marmorata telangiectatica congenital syndrome.

MCAP is characterized by MEG frequently associated with other MCD, capillary malformations especially midline nevus flammeus of the upper lip or nose, syndactyly of toes (and less often fingers, 3–4 or 2–3–4), variable segmental body overgrowth and soft or doughy skin with connective tissue laxity. Cranio-facial features included macrocephaly with dolichocephaly, frontal bossing, deep-set eyes, and full cheeks (Figures 12A–E,I–L). The diagnosis of Megalencephaly-capillary MCAP can be assumed when MEG and either vascular malformations or syndactyly are present (36, 172, 183). So far, the risk of malignancy in this entity appears to be lower than in the majority of other overgrowth syndromes (36).

On brain MRI, besides symmetric or asymmetric MEG (corresponding to diffuse overgrowth of brain structures), PMG is also identified in a significant proportion of patients, usually with a perisylvian distribution, although it can be more extensive in some cases. Ventriculomegaly (communicating hydrocephalus) is additionally identified, and variable degrees of brain and ventricular asymmetry may be seen. The corpus callosum is either significantly (mega-corpus callosum) or moderately thick. Of note, these neuroimaging features overlap with the MPPH syndrome (see below) which however is due to mutations in a different gene (36). Regular follow-up MRIs are recommended as cerebellar tonsillar ectopia and hydrocephalus (obstructive hydrocephalus) can also develop (184).

##### CLOVES Syndrome

CLOVE(S) syndrome (acronym for Congenital Lipomatous asymmetric Overgrowth of the trunk, lymphatic, capillary, venous, and combined-type Vascular malformations, Epidermal nevi, Skeletal, and spinal anomalies) is one of the several now-distinct entities previously included in the heterogeneous designation of Proteus syndrome (PS) due to the phenotypic overlap between the two conditions (185–187). It is due to somatic mutation in *PIK3CA*, therefore falling in the PROS spectrum and resulting in dramatic overgrowth of both hands and feet, extensive capillary malformations of the skin and several epidermal nevi. MEG/HMEG may occur, as well as MCD and agenesis or dysgenesis of the corpus callosum, often associated with asymmetric enlargement of the face (188). As above mentioned, spinal abnormalities are also a key feature of CLOVES and include scoliosis, neural tube defects as well as high-flow spinal or para-spinal arterio-venous malformations, potentially causing ischemic myelopathy (189). Of note, these vascular malformations do not occur in PS (187).

##### Klippel-Trenaunay Syndrome

Similar to CLOVES, Klippel-Trenaunay syndrome (KTS) is due to somatic mutations in *PIK3CA* (190), becoming its most relevant differential diagnosis. Klippel-Trenaunay syndrome, also known as angio-osteohypertrophy syndrome, is characterized by a triad of capillary malformations with a port-wine stain appearance mostly involving lower limbs, asymmetrical soft tissue/osseous hypertrophy, and venous varicosities. Other slow-flow vascular lesions (venous, lymphatic, or mixed malformations) are frequently present, as well as intra-abdominal hemangiomas and digital abnormalities. Other clinical features occasionally reported include seizures and DD (180, 181). It is important not to misdiagnose Klippel–Trenaunay syndrome with Parkes Weber syndrome (also known as Klippel–Trenaunay–Weber syndrome) that in opposition with the former syndrome, is due to RASA1 mutations and is characterized by the presence of high-flow vascular malformations (191).

Neuroimaging features include unilateral megalencephaly/HMG (192, 193), single or multiple brain and/or spinal cavernomas as well as intracranial aneurysms (194–197). The incidence of ischemic stroke appears to be increased in this syndrome due to paradoxical emboli in the context of thromboembolism (198).

##### Megalencephaly-Polymicrogyria-Pigmentary Mosaicism Syndrome (MPPM)

The name of this syndrome has been recently proposed to describe patients with MEG or HMEG and pigmentary mosaicism of the skin due to mosaic, moderate-grade gain of function mutations in the *MTOR* gene (179). This entity probably represents most of the previous cases designated as hypomelanosis of Ito (179) (Figures 12F,G).

##### Proteus Syndrome

Proteus syndrome (OMIM #176920) is a rare overgrowth syndrome characterized by segmental overgrowth of multiple tissues resulting in vascular malformations, lipomas, hyperpigmentation, and various types of nevi. In particular, cerebriform connective tissue nevi (CCTN) are present in most individuals with PS and are nearly pathognomonic. Proteus syndrome has been related to only one somatic activating mutation (c.49G>A,p.Glu17Lys) in *AKT1* (199). Macrocephaly may be present as well as unilateral MEG/HMEG with cortical dysgenesis (200–204). However, no AKT1 mutations have been identified in affected brain tissues so far.

There is also a higher risk of intracranial meningiomas in this condition (204, 205). Dolichocephaly, hyperostosis of the skull and the external auditory meatus as well as unilateral condylar hypoplasia can also occur (204). Finally, spinal dysmorphism, characterized by abnormal vertebral bodies and scoliosis, as well as spinal lipomatosis is another common imaging feature of this disorder (198, 200).

#### Overgrowth Syndromes Without Vascular/Skin Features

This group of overgrowth syndromes has been widely reviewed elsewhere (206). We briefly discuss those that are clinically recognizable and may require a specific diagnostic work-up, e.g., BWS.

##### Beckwith-Wiedemann Syndrome

Beckwith-Wiedemann syndrome (OMIM# 130650) is a genomic imprinting disorder mapped to 11p15.5 region, characterized by generalized or lateralized overgrowth (hemihyperplasia), macroglossia, omphalocele, visceromegaly, kidney defects, neonatal hypoglycaemia, and predisposition to embryonal tumors. Typical craniofacial features include ear creases/pits and infraorbital creases. In addition, cleft palate, infraorbital creases, and midface retrusion may also occur (207). More commonly, linear growth slows around the age of 8 years, and the facial and physical features become less obvious with age. The clinical diagnosis of BWS has traditionally required the presence of at least 2 major and 1 minor criteria (208). Advances in molecular testing suggest a broader phenotype with possible subtle clinical presentations and an updated Consensus Statement for the clinical and molecular diagnosis and management of BWS has been recently outlined (209).

Different imprinting mechanisms responsible for BWS include (i) loss of methylation of the imprinting center 2 on the maternal chromosome, (ii) gain of methylation of imprinting center 1 on the maternal chromosome, and (iii) paternal uniparental disomy of 11p15.5. CDKN1C mutations (iv) are present in only 5% of sporadic BWS cases but in 40% of patients with a family history of BWS (208).

It is extremely important to recognize BSW because identification of the molecular defects within the imprinted 11p15.5 region can predict familial recurrence and the risk and type of embryonal tumor (209).

Brain malformations, including posterior fossa abnormalities and callosal dysgenesis have been occasionally described in this syndrome (210).

##### Sotos Syndrome

Sotos syndrome (OMIM# 117550) is an overgrowth syndrome characterized by a distinctive facial appearance that includes broad and prominent forehead with a dolichocephalic head shape, sparse frontotemporal hair, downslanting palpebral fissures, malar flushing, long and narrow face, and a pointed chin (that becomes prominent and squared over years) (211, 212). Intellectual disability is usually mild to moderate (213). Up to a third of patients have additional CNS anomalies (e.g., ventriculomegaly) and seizures, congenital heart defects, scoliosis, and renal anomalies. Cancer occurrence, in particular acute myelocytic leukemia, has been reported in about 3% of cases (214). Sotos syndrome is due to heterozygous mutations in *NSD1*, a transcription coregulator gene, encoding the nuclear receptor binding SET domain protein 1 with histone methyltransferase function. Although this syndrome can be transmitted in an autosomal dominant manner, in the vast majority of cases it is caused by *de novo* germline mutations. Brain MRI of patients with Sotos syndrome shows a large brain with normal cortical appearance. Ventriculomegaly, presence of a cavum of septum pellucidum/cavum vergae and enlargement of extra-cerebral spaces are also common, as well as thinning of the posterior portions of the corpus callosum (215, 216). Periventricular heterotopias have also been reported in this syndrome (215) (Supplementary Figure 3).

In the recent years, novel disorders displaying features reminiscent of Sotos syndrome have been identified. Among these, Malan syndrome (MIM# 614753, also called Sotos-2 syndrome), due to mutations in *NFIX*, is characterized by an overlap of the facial phenotype with NSD1-positive Sotos syndrome (now called Sotos-1), including prominent forehead, high anterior hairline, downslanting palpebral fissures and prominent chin (217, 218).

##### Weaver Syndrome

Weaver syndrome (OMIM# 277590) is a further overgrowth disorder that shares some overlap craniofacial and clinical features with Sotos syndrome. It is due to heterozygous mutations in *EZH2*, encoding a member of the Polycomb-group (PcG) family, which has transcriptional repressive function (219, 220). The typical facial gestalt of Weaver syndrome includes broad forehead and face, hypertelorism, almond-shaped and downslanting palpebral fissures, prominent wide philtrum, retrognathia, and a deep horizontal chin groove. The latter features often help to distinguish Weaver from Sotos syndrome (221). Other features can include advanced bone age, large hands and feet, camptodactyly, deep-set nails, low-pitched cry, umbilical hernia, and soft doughy skin. Intellectual disability is virtually present in all cases.

Neuroimaging findings have only been reported in a few patients with Weaver syndrome, but include most often ventriculomegaly, periventricular leukomalacia, cerebellar abnormalities, and scoliosis (219). Cortical abnormalities (PMG and pachygyria) have also been occasionally described (219, 222, 223).

Embryonal and hematologic tumors have been reported in up to 5% of the patients (224, 225), though no specific protocol for cancer surveillance has yet been validated (226).

### PI3K-AKT-MTOR- Related Megalencephaly

#### PTEN Hamartoma Tumor Syndrome

Germline heterozygous mutations in *PTEN*, a tumor suppressor gene that antagonizes the (PI3K)/AKT signaling, are linked to a wide range of MEG phenotypes, collectively referred as PTEN hamartoma tumor syndrome (PHTS). They include Cowden syndrome (CS; OMIM# 158350), Bannayan-Riley-Ruvalcaba syndrome (BRRS; OMIM# 153480), ASD with macrocephaly, adult Lhermitte-Duclos disease, and occasionally segmental overgrowth (due to somatic PTEN mutations) (227–229).

Although clinical manifestations of PHTS differ significantly, all four syndromes are characterized by aberrant tissue growth likely related to loss of tumor suppressor role of *PTEN*.

Subjects with CS typically present in early adulthood with macrocephaly, characteristic skin lesions, development of multiple benign hamartomas, and an increased risk of certain cancers, particularly of breast, uterus, and thyroid. Diagnostic criteria for CS have been developed and are available online at the National Comprehensive Cancer Network (NCCN).

On the other hand, BRRS is typically diagnosed in children with macrocephaly, hamartomas (including lipomas, haemangiomas, or intestinal polyps), penile freckling in males, DD/ID and ASD (230). Interestingly, ASD is thought to have a distinct neurobehavioral phenotype in PHTS (81). Macrocephaly is often present at birth and is the most common feature among the diverse clinical PHTS phenotypes. About two-third of children have OFC between +4 SD and 6 SD (227, 231), reaching an average at adult age of 60.0 cm in females and 62.8 cm in males (232). Conversely, height and weight measurements are always in the normal range (233). A recent retrospective study has revealed that the most common clinical features include dermatological findings (66%), gastrointestinal symptoms (34%), and abnormal thyroid imaging (26%) (227). Notably, the association of macrocephaly with penile flecking, should alert pediatricians regarding a possible underlying BRRS. Other relevant dermatological features include café-au-lait spots, skin tags, nevi, papillomatous papules, haemangiomas, trichilemmomas, hyperpigmented, and hypopigmented lesions. The presence of abdominal pain, rectal bleeding, and constipation should induce to request a colonoscopy to screen for possible polyps although they are rare during childhood. Thyroid cysts and nodules have been detected in almost a third of patients and thyroid cancer has been occasionally reported in PHTS subjects <18-year-old. Specific cancer surveillance indeed includes annual thyroid ultrasound even in children. More recently, clinical criteria taking into account the full phenotypic spectrum of PHTS disorders have been proposed in order to maintain their overview and overcome the limitations of individual classifications (228).

Neuroimaging abnormalities are present in at least half of the affected patients (221), consisting mainly of bilateral symmetric MEG with corpus callosum thickening and tonsillar ectopia (Figures 13A–C). Rarely, unilateral MEG or other malformations of cortical development have also been described, including PMG (234–237). Other common imaging features include multifocal periventricular white matter signal changes, dilated perivascular spaces (227, 238) as well as intracranial and spinal vascular malformations (including developmental venous anomalies and dural arteriovenous fistulas) (231). Patients with PHTS may demonstrate a lesion in the posterior fossa compatible with Lhermitte-Duclos disease (dysplastic gangliocytoma of the cerebellum) (236). This entity usually presents in the third or fourth decade and although it may occur sporadically in near half of adult cases, it is considered an important criterion of PHTS in that age group (228). Briefly, it is a slow-growing lesion currently classified as a WHO grade I tumor and characterized on MRI by a “striated folial pattern” involving both gray and white-matter, with variable contrast enhancement and some mass effect over the IV ventricle.

Of note, pediatric and adult clinical scoring systems (Cleveland Clinic PTEN Risk Calculation tools) for an accurate *a priori* selection of patients for *PTEN* mutation testing have been formulated (239). Using this score system, presence of macrocephaly associated with at least one of the additional four criteria has been proved to be highly sensitive criteria to guide PTEN mutation in this specific age group (239).

#### MCAP (Germline Variants)

About 10% of cases with MCAP harbor germline variants in *PIK3CA*, resulting in mild PI3K-AKT-mTOR activation. Accordingly, their phenotype includes MEG and ID, in absence of other typical features such as vascular anomalies and limb overgrowth, usually observed in MCAP cases due to somatic PIK3CA mutations (182).

#### Megalencephaly-Polymicrogyria-Polydactyly-Hydrocephalus

Megalencephaly-polymicrogyria-polydactyly-hydrocephalus (MPPH) syndrome is caused by mutations, in order of frequency, of either *PIK3R2, AKT3*, or *CCND2* genes with consequent activation of the PI3K-AKT-mTOR pathway (172). Differently from MCAP, in which 90% of mutations are somatic, mutations leading to MPPH are more frequently *de novo* germinal variants (179). Although MCAP and MPPH are caused by mutations involving different components of the mTOR pathway and MPPH is not included in the group of PROS disorders, their clinical features partially overlap. Indeed, MPHH is also characterized by MEG/HMEG, bilateral PMG, ventriculomegaly, mild to severe ID and epilepsy, and sometimes post-axial polydactyly. However, differently from MCAP, vascular malformations, syndactyly, and heterotopia are not usually present in MPPH (36).

The most severe MPPH phenotype is observed in children with CCND2 mutations. Conversely, PIK3R2 cases usually show only ventriculomegaly and milder cognitive impairment. Somatic mutations in *AKT3* also account for a minority of MPPH cases, usually presenting with MEG without PMG, whereas somatic PIK3R2 mutations have been associated with PMG and normal OFC (240).

#### Non-syndromic MEG

Non-syndromic MEG with ID, with possible autism and epilepsy has been reported in about 20 individuals harboring germline MTOR mutation (241). In addition, germline mutations in *AKT3* account for a minority of non-syndromic MEG (242) and just two MEG cases have been found to carry germline PIK3CA variants (182) (Figures 12M,N).

#### Hemimegalencephaly

In contrast to non-syndromic MEG, HMEG is exclusively due to somatic mutations in *PIK3CA, MTOR* and *AKT3* (172, 182, 241, 243) that result in a clinically severe phenotype, characterized by intractable epilepsy, severe ID, ipsilateral white matter anomalies, dilated/dysmorphic lateral ventricle, and cortical dysplasia. Notably, FCD and HMEG belong to the same phenotypic spectrum due to somatic mutations in the PI3K-AKTmTOR genes (Figures 11, 13D–F).

## Practical Approach to the Diagnosis of Macrocephaly

**Figure 14:**
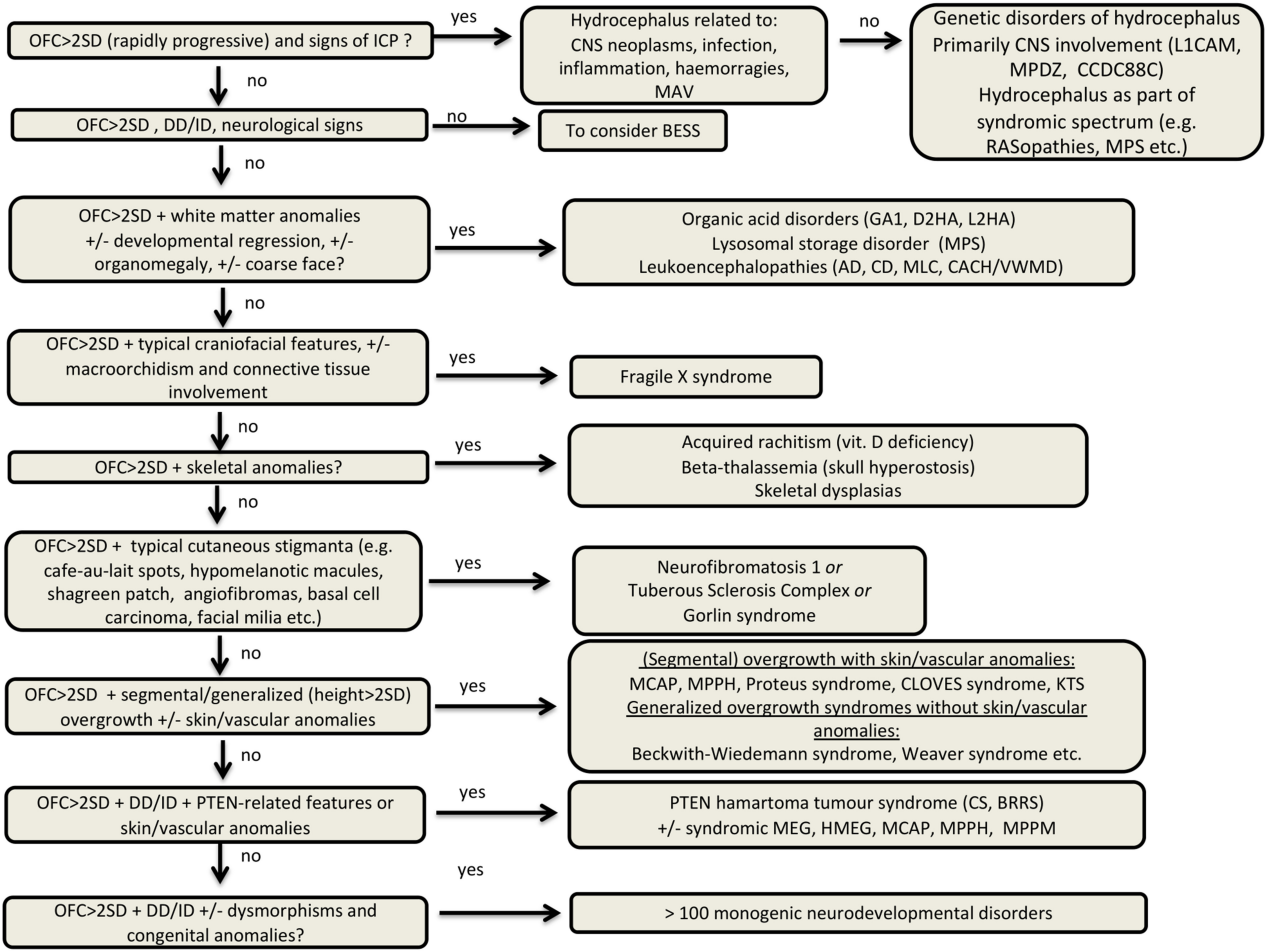
Diagnostic algorithm of macrocephaly. BESS, Benign enlargement of subarachnoid spaces; BRRS, Bannayan-Riley-Ruvalcaba syndrome; CS, Cowden syndrome; CNS, central nervous system; D2HA, D2-hydroxyglutaric aciduria; L2HA, L2-hydroxyglutaric aciduria; DD, developmental delay; KTS, Klippel-Trenaunay syndrome; ICP, increased intracranial pressure; GA1, glutaric aciduria type 1; MAV, arteriovenous malformations; MPS, mucopolysaccharidosis; AD, Alexander disease; CD, Canavan disease; MLC, megalencephalic leukoencephalopathy with subcortical cysts; MCAP, megalencephaly-capillary malformation syndrome; MPPH, megalencephaly-polymicrogyria-polydactyly-hydrocephalus syndrome; MPPM, megalencephaly-polymicrogyria and pigmentary mosaicism; CACH/VWMD, childhood ataxia with central hypomyelination/vanishing white matter disease.

An accurate diagnosis of patients with macrocephaly requires a multidisciplinary team approach. First, a careful review of medical history and physical examination, involving the pediatrician, neurologist, and clinical geneticist are the first steps to generate a thorough phenotypic characterization. Concurrently, brain MRI should be carefully reviewed by an expert neuroradiologist in order to differentiate between true megalencephaly (either developmental or metabolic) and secondary causes of macrocrania, as well as to detect any associated imaging features suggestive of a particular group of disorders or even specific entities. A proposed algorithm for macrocrania evaluation is presented in Figure 14. After initial clinical and neuroimaging assessment, the subsequent diagnostic work-up should be customized for each suspected macrocephaly subgroup.

If hydrocephalus is found on brain MRI and acquired causes are ruled out, a careful neuroradiological assessment may identify neuroradiological features, possibly suggesting a specific genetic etiology, such as aqueductal stenosis due to L1CAM mutations (Supplementary Table 1). In parallel, a clinical evaluation may unveil dysmorphisms and congenital features of specific disorders associated with hydrocephalus, like Pettigrew syndrome. If clinical and neuroradiological evaluations are not suggestive of a specific disorder but a genetic etiology is highly suspected, a hydrocephalus NGS panel or exome sequencing may be required.

In the presence of overgrowth, it is important to recognize whether it is generalized or segmental and identify possible associated vascular/cutaneous anomalies because genetic work-up is different for the two main overgrowth subgroups. Indeed, the presence of segmental overgrowth plus vascular/skin anomalies should orient clinicians toward a PI3K-AKT-mTOR pathway-related disorder usually due to somatic mutations (with the exception of a few MCAP cases related to germline AKT3 mutations). Accordingly, genetic testing in these cases should be pursued on DNA extracted from specimen of affected tissues (e.g., fibroblast, buccal swab, brain tissues if available). Among the overgrowth syndromes, it is also important to recognize clinical hallmarks of Beckwith-Wiedemann syndrome since it is an imprinting disorder, requiring specific genetic work-up (Table 1). Some craniofacial features might help to recognize specific overgrowth disorders such as Sotos and Weaver syndromes. However, other monogenic disorders presenting with a generalized overgrowth could be investigated by an overgrowth panel or exome sequencing.

**Table 1:** Genetic investigations in macrocephaly-related disorders.

**Macrocephaly subgroups**	**Investigations**
**Macrocephaly related to CSF expansion**	
Benign enlargement of subarachnoid spaces	None
Hydrocephalus due to acquired cause	To settle according to the underlying diagnosis
Hydrocephalus due to genetic etiology	Target gene sequencing or hydrocephalus panel
Hydrocephalus due vein of galen aneurysmal malformation	*RASA1* and *EPHB4* gene testing (+/—MLPA)
**Macrocephaly with: +/– white matter anomalies**, **+/– developmental regression**, **+/– organomegaly**, **+/– coarse face**	
Organic acid disorders (GA1, D2HA, L2HA)	Urine organic acid, plasma and urine amino acids, acylcarnitine profile; Target gene testing/Gene panel testing
Lysosomal storage disorder (MPS)	Lysosomal enzyme testing on leukocyte/gene panel Target gene testing/gene panel
Leukoencephalopathies (AD, CD, MLC, CACH/VWMD)	Target gene testing or leukodystrophy panel
**Fragile X syndrome**	FMR1 gene testing
**Macrocephaly and skeletal anomalies**	
(Acquired) rickets	Vitamin D3 dosage
Beta-thalassemia	Hemoglobin electrophoresis, HBB gene testing
Skeletal dysplasias	Target genetic testing (e.g., Achondroplasia) or skeletal gene panel/ES
**Neurocutaneous syndromes**	
Neurofibromatosis 1	NF1/SPRED1 gene testing
Tuberous sclerosis complex	TSC1, TSC2 gene testing
Gorlin syndrome	PTCH1, SUFU gene testing
**Macrocephaly and segmental overgrowth syndromes**	
***(Segmental) overgrowth with skin/vascular anomalies***	
MCAP	PIK3CA gene testing on affected specimen^*^ (somatic mutations 90%)
MPPH	AKT3 gene testing on blood (germline mutations in a small subset of children)
Proteus syndrome	AKT1 mutation (c.49G>A,p.Glu17Lys) testing on affected specimen^*^ (100% somatic mutation)
CLOVES syndrome	PIK3CA gene testing on affected specimen^*^ (100% somatic mutations)
KTS	PIK3CA gene testing on affected specimen^*^ (100% somatic mutations)
***Generalized overgrowth syndromes without skin/vascular anomalies***	
Beckwith-Wiedemann syndrome	Methylation analysis (loss of maternal methylation at IC2 (50%, IC1 (5), loss of maternal methylation at IC2, and gain of methylation at CI1 (paternal UDP) (20%) Heterozygous maternal CDKN1C pathogenic variants Microdeletion, microduplication, paternal UDP (9%) Cytogenetic duplication, inversion or translocation of 11p15.5 (<1%)
Other overgrowth syndromes (Weaver syndrome, Sotos syndromes, etc.)	Target gene testing or gene panel sequencing/ES
**Macrocephaly and clinical features of PTEN-/PI3K-AKTmTOR related disorders**	
PTEN hamartoma tumor syndrome (CS, BRRS)	PTEN gene testing, +/**–** MLPA on blood (germline mutations)
+/**–** syndromic MEG	MTOR (+), AKT3, (PIK3CA) gene testing on blood (germline mutations)
HMEG	MTOR, AKT3, PIK3CA gene testing on blood (somatic mutations)
MCAP	PIK3CA gene testing on blood (10% germline mutations)
MPPH	PIK3R2(++), CCND2(+), AKT3 gene testing on blood (germline mutations) MTOR and AKT3 gene testing on affected specimen^*^ (occasionally somatic mutations)
MPPM	MTOR mutations (p.Cys1483Tyr/Phe, p.Thr1977Ile) gene testing on affected specimen^*^
**Macrocephaly + DD/ID**, **+/- dysmorphisms and congenital anomalies (>100 monogenic disorders in OMIM database)**	Chromosomal microarray Macrocephaly panel/ES

*BESS, Benign enlargement of subarachnoid spaces; BRRS, Bannayan-Riley-Ruvalcaba syndrome; CS, Cowden syndrome; CNS, central nervous system; D2HA, D2-hydroxyglutaric aciduria; KTS, Klippel-Trenaunay syndrome, L2HA, L2-hydroxyglutaric aciduria; GA1, glutaric aciduria type 1; GM, Gangliosidosis type 2; ES, exome sequencing; MPS, mucopolysaccharidosis; AD, Alexander disease; CD, Canavan disease; MLC, megalencephalic leukoencephalopathy with subcortical cysts; MLPA, multiplex ligation-dependent probe amplification, MCAP, megalencephaly-capillary malformation syndrome; MPPH, megalencephaly-polymicrogyria-polydactyly-hydrocephalus syndrome; MPPM, megalencephaly-polymicrogyria and pigmentary mosaicism; CACH/VWMD, childhood ataxia with central hypomyelination/vanishing white matter disease.*

* *Buccal swab, saliva, fibroblasts, resected brain tissue*.

The presence of developmental regression, coarse face, and specific white matter anomalies on brain MRI are all features suggestive of a possible metabolic disorder or leukoencephalopathy. The presence of organomegaly should be assessed by abdominal ultrasound when these disorders are suspected. Brain MRI pattern recognition, complemented by proton spectroscopy in some cases, can suggest the diagnosis or, at least, reduce the differential diagnosis. Metabolic testing for these disorders include urine organic acids, plasma and urine amino acids, and acylcarnitine profile for organic acid disorders and lysosomal enzyme dosage on leukocytes if a lysosomal disorder is suspected. The diagnosis of metabolic disorders and leukoencephalophathies can be eventually confirmed by molecular testing.

Other conditions presenting with macrocephaly that should be clinically recognized are the Neurocutaneous syndromes, namely NF1, TSC, and GS. In presence of the typical hallmarks of these disorders a targeted molecular testing should be requested. Similarly, FXS could be clinically recognized and requires a specific molecular testing.

The presence of macrocephaly and skeletal anomalies often requires the involvement of radiologists and clinicians expert in skeletal dysplasia. However, at least a few conditions should be recognized by all pediatricians and properly investigated. The presence of bowed legs, bell-shaped chest, thickened wrist, and ankles should rise the suspicious of acquired rickets and vitamin D3 level should be assessed. The association of microcytic anemia and macrocephaly should suggest an underlying diagnosis of untreated Beta-thalassemia. Hence, hemoglobin electrophoresis and molecular genetic testing should be performed. Among various skeletal dysplasias presenting with macrocephaly, Achondroplasia is by far the most common and easily recognized given the association of macrocephaly, short stature, rhizomelic shortening of limbs, and typical craniofacial features. Hypochodroplasia, the least severe form, may be clinically recognized as well. When these disorders are suspected, molecular testing of *FGFR3* should be pursued. In absence of hints of other skeletal dysplasia (e.g., osteopetrosis and others) a gene panel for skeletal dysplasias or exome sequencing could be requested.

The association of macrocephaly with DD/ID includes a clinically and genetically heterogenous group of disorders with more than 150 syndromes listed in the OMIM database. Among these, it is extremely important to recognize the PI3K-AKT-mTOR pathway-related disorders, namely the MCAP, and HMEG that are associated with somatic mutations. Overall, the molecular diagnosis of these disorders could be achieved either through a target gene testing or more commonly by a gene panel (PI3K-AKT-mTOR panel), but it is mandatory to keep in mind the suspected clinical diagnosis given the possibility of underlying somatic mutations that would be missed by testing DNA extracted from blood.

In the absence of clinical cues for the above-mentioned disorders or other specific genetic syndromes, the association of macrocephaly, DD/ID, and possible dysmorphisms should be investigated by a chromosomal microarray and if it yields negative or unremarkable results a macrocephaly panel or exome sequencing should be pursued. Although the exact diagnostic rate of copy number variants in macrocephaly remains elusive, it is well known that many causative genes, such as transcriptional repressor and tumor suppressor genes, act through a loss-of-function mechanism and therefore their haploinsufficiency leads to a clinical phenotype including MEG. If available, exome sequencing may be preferred to macrocephaly panels, first looking at genes known to be associated with macrocephaly through a bioinformatic customized panel, and then in case of negative results, expanding the analysis to include a broader set of genes not yet associated with a specific phenotype and potential new candidate genes. Genetic investigations for each macrocephaly group are summarized in Table 1.

## Management, Treatment, and Future Directives

Management of macrocephaly greatly varies according to different etiologies. An extensive overview of management of all disorders is behind the scope of this review.

We mostly would like to stress the importance of cancer surveillance in some disorders related to macrocephaly including PHTS, GS, NF1, TSC, L-2-HGA, and many overgrowth syndromes such as BWS and Sotos syndrome among others. Specific protocols exist for most of these disorders and should be carefully followed once a diagnosis has been molecularly confirmed (226, 231).

Supportive management (e.g., occupational therapy, physiotherapy, and speech therapy) is the only available treatment for the majority of neurodevelopmental disorders presenting with macrocephaly.

However, metabolic disorders are a group of disorder for which enzyme replacement therapy and in some cases gene therapies have been successfully adopted. For instance, enzyme replacement therapy is currently available for MPSI, MPS II, MPS VI, and MPS IVA (244, 245). In support of conventional therapies, new therapeutic methods have been developed for MPS, such as new recombinant enzymes that can penetrate the blood-brain barrier, hematopoietic stem cell transplantation, gene therapy using a viral vector system or gene editing (245–247). The ISRIB, an activator of eIF2B, is a promising molecule in the treatment of VWMD since it can stabilize the mutant eIF2B and restores the residual catalytic activity to wild-type levels (248, 249). Moreover, there has been a growing interest in mTOR inhibitors as promising antiepileptogenic therapies, such as rapamycin and everolimus in TSC (250, 251). Given the important role of mTOR in the development of several different forms of MEG, it might be possible that mTOR inhibitors will be used in the next future to treat a broader range of MTOR-related disorders associated with epilepsy and MEG.

Another promising therapeutic approach includes antisense oligonucleotides (ASOs) that are short, synthetic, single-stranded oligodeoxynucleotides able to alter RNA, modifying protein expression through several distinct mechanisms. They have been successfully used in several neurological disorders (e.g., Spinal muscular atrophy and Huntington disease) and there are encouraging results in the mouse model of AD (252). It is reasonable to expect in the coming years novel research trials with ASOs in several neurodevelopmental disorders presenting with macrocephaly, such as MTOR-related disorders and FSX.

## Conclusion

Macrocephaly encompasses an extremely heterogeneous group of disorders with a wide range of etiologies, radiologic characteristics, clinical features, and neurodevelopmental outcomes. Recent advances in genetic methods such as exome and genome sequencing have allowed the continuous identification of novel disorders associated with macrocephaly, providing insight into the complexity of brain development. Combining imaging, neurological and dysmorphological assessments are crucial to promptly recognize a specific macrocephaly class and in turn pursue targeted diagnostic testing. Accurate classification of macrocephaly is a key for diagnosis, workup, and prognosis. However, we still do not have a standard recognized classification system that integrates neuroimaging, clinical, molecular, genetic, and developmental biological criteria. Future studies are needed to gather and integrate all this data into practical and resolutive diagnostic approaches for patients with macrocephaly.

## Author Contributions

All authors listed have made a substantial, direct, and intellectual contribution to the work and approved it for publication.

## Conflict of Interest

The authors declare that the research was conducted in the absence of any commercial or financial relationships that could be construed as a potential conflict of interest.

## Publisher's Note

All claims expressed in this article are solely those of the authors and do not necessarily represent those of their affiliated organizations, or those of the publisher, the editors and the reviewers. Any product that may be evaluated in this article, or claim that may be made by its manufacturer, is not guaranteed or endorsed by the publisher.

## Supplementary Material

Supplementary Figure 1 Imaging approach to secondary causes of macrocrania. BEES, Benign enlargement of subarachnoid spaces in infancy; CSF, cerebrospinal fluid; SAS, subarachnoid spaces.

Supplementary Figure 2 Imaging approach to true megalencephaly. A, anterior; CC, corpus callosum; GM, gray matter; H-MRS, proton magnetic resonance spectroscopy; HMEG, hemimegalencephaly; MEG, megalencephaly; NAA, N-acetylaspartate; P, posterior; WM, white matter.

Supplementary Figure 3 Imaging characteristics of Sotos Syndrome. Axial T2WI **(A–C)** as well as sagittal **(D)** and axial **(E,F)** T1WI of an 8-year-old child with Sotos syndrome reveal mild ventriculomegaly (asterisks) and enlargement of the subarachnoid spaces mainly in the temporopolar region (black arrowheads). Also note thinning of the corpus callosum (open arrows) and presence of a cavum septum pellucidum/vergae (circles) as well as dilated perivascular spaces (thick white arrows) mainly in the anterior cerebral white matter.

Supplementary Table 1 Acquired and congenital causes of hydrocephalus with proposed underlying mechanism.

## References

[1] Medina LS Frawley K Zurakowski D Buttros D DeGrauw AJC Crone KR. Children with macrocrania: clinical and imaging predictors of disorders requiring surgery. Am J Neuroradiol. (2001) 22:564–70. 11237985 PMC7976845

[2] Tan AP Mankad K Gonçalves FG Talenti G Alexia E. Macrocephaly: solving the diagnostic dilemma. Top Magn Reson Imaging. (2018) 27:197–217. 10.1097/RMR.0000000000000170 30086108

[3] Nellhaus G. Head circumference from birth to eighteen years. Pediatrics. (1968) 41:106–14. 5635472

[4] DeMyer W. Megalencephaly: types, clinical syndromes, and management. Pediatr Neurol. (1986) 2:321–8. 10.1016/0887-8994(86)90072-X 3334205

[5] Severino M Geraldo AF Utz N Tortora D Pogledic I Klonowski W. Definitions and classification of malformations of cortical development: practical guidelines. Brain. (2020) 143:2874–94. 10.1093/brain/awaa174 32779696 PMC7586092

[6] Yilmazbaş P Gökçay G Eren T Karapinar E Kural B. Macrocephaly diagnosed during well child visits. Pediatr Int. (2018) 60:474–7. 10.1111/ped.13543 29498760

[7] Pirozzi F Nelson B Mirzaa G. From microcephaly to megalencephaly: determinants of brain size. Dialogues Clin Neurosci. (2018) 20:267–82. 10.31887/dcns.2018.20.4/gmirzaa 30936767 PMC6436952

[8] Williams CA Dagli A Battaglia A. Genetic disorders associated with macrocephaly. Am J Med Genet A. (2008) 146:2023–37. 10.1002/ajmg.a.32434 18629877

[9] Gooskens RH Willemse J Bijlsma JB. W Hanlo P. Megalencephaly: definition and classification. Brain Dev. (1988) 10:1–7. 10.1016/S0387-7604(88)80037-8 3285723

[10] Mirzaa GM Poduri A. Megalencephaly and hemimegalencephaly: breakthroughs in molecular etiology. Am J Med Genet C Semin Med Genet. (2014) 166C:156–72. 10.1002/ajmg.c.31401 24888963

[11] Winden KD Yuskaitis CJ Poduri A. Megalencephaly and macrocephaly. Semin Neurol. (2015) 35:277–87. 10.1055/s-0035-1552622 26060907

[12] Keppler-Noreuil KM Rios JJ Parker VER Semple RK Lindhurst MJ Sapp JC. PIK3CA-related overgrowth spectrum (PROS): diagnostic and testing eligibility criteria, differential diagnosis, and evaluation. Am J Med Genet A. (2015) 167A:287–95. 10.1002/ajmg.a.36836 25557259 PMC4480633

[13] Mirzaa G Roy A Dobyns WB Millen K Hevner RF. Hemimegalencephaly and dysplastic megalencephaly. In: Adle-Biassette H Harding BN Golden JA Gray F Keohane K, editors. Developmental Neuropathology, International Society of Neuropathology Series. John Wiley and Sons, Ltd., 55–61.

[14] Scala M Torella A Severino M Morana G Castello R Accogli A. Three *de novo* DDX3X variants associated with distinctive brain developmental abnormalities and brain tumor in intellectually disabled females. Eur J Hum Genet. (2019) 27:1254–9. 10.1038/s41431-019-0392-7 30936465 PMC6777618

[15] Stiles J Jernigan TL. The basics of brain development. Neuropsychol Rev. (2010) 20:327–48. 10.1007/s11065-010-9148-4 21042938 PMC2989000

[16] Gardner-Medwin D. Fetal and neonatal neurology and neurosurgery. J Neurol Neurosurg Psychiatry. (1989) 52:1323–1323. 10.1136/jnnp.52.11.132316457521

[17] Rivers E. Child development, stages of growth. In: Encyclopedia of Forensic and Legal Medicine. 2nd ed. (2016). p. 539–57. Available online at: https://www.encyclopedia.com/education/encyclopedias-almanacs-transcripts-and-maps/child-development-stages-growth (accessed May 28, 2021).

[18] Klein S Sharifi-Hannauer P Martinez-Agosto JA. Macrocephaly as a clinical indicator of genetic subtypes in autism. Autism Res. (2013) 6:51–6. 10.1002/aur.1266 23361946 PMC3581311

[19] Amaral DG Li D Libero L Solomon M Van de Water J Mastergeorge A Naigles L. In pursuit of neurophenotypes: the consequences of having autism and a big brain. Autism Res. (2017) 10:711–22. 10.1002/aur.1755 28239961 PMC5520638

[20] Gilbert J Man HY. Fundamental elements in autism: from neurogenesis and neurite growth to synaptic plasticity. Front Cell Neurosci. (2017) 11:359. 10.3389/fncel.2017.00359 29209173 PMC5701944

[21] Courchesne E Mouton PR Calhoun ME Semendeferi K Ahrens-Barbeau C Hallet MJ. Neuron number and size in prefrontal cortex of children with autism. J Am Med Assoc. (2011) 306:2001–10. 10.1001/jama.2011.1638 22068992

[22] Hutsler JJ Love T Zhang H. Histological and magnetic resonance imaging assessment of cortical layering and thickness in autism spectrum disorders. Biol Psychiatry. (2007) 61:449–57. 10.1016/j.biopsych.2006.01.015 16580643

[23] Jan YN Jan LY. Branching out: mechanisms of dendritic arborization. Nat Rev Neurosci. (2010) 11:316–28. 10.1038/NRN2836 20404840 PMC3079328

[24] Sniderman A. Abnormal head growth. Pediatr Rev. (2010) 31:382–4. 10.1542/pir.31-9-382 20810704

[25] Perera PJ Fernando MP Samaranayake R. Head circumference during infancy in a birth cohort of Sri Lankan children: are we using the correct chart? Ceylon Med J. (2014) 59:136–8. 10.4038/cmj.v59i4.7867 25556411

[26] Elmali F Altunay C Mazicioglu MM Kondolot M Ozturk A Kurtoglu S. Head circumference growth reference charts for Turkish children aged 0–84 months. Pediatr Neurol. (2012) 46:307–11. 10.1016/j.pediatrneurol.2012.02.016 22520352

[27] Bertino E Di Nicola P Varalda A Occhi L Giuliani F Coscia A. Neonatal growth charts. J Matern Fetal Neonatal Med. (2012). 25:67–9. 10.3109/14767058.2012.664889 22348405

[28] Van Den Broeck J Willie D Younger N. The World Health Organization child growth standards: expected implications for clinical and epidemiological research. Eur J Pediatr. (2009) 168:247–51. 10.1007/s00431-008-0796-9 18670787

[29] James HE Perszyk AA MacGregor TL Aldana PR. The value of head circumference measurements after 36 months of age: a clinical report and review of practice patterns. J Neurosurg Pediatr. (2015) 16:186–94. 10.3171/2014.12.PEDS14251 25932781

[30] Moore BD Slopis JM Jackson EF De Winter AE Leeds NE. Brain volume in children with neurofibromatosis type 1: relation to neuropsychological status. Neurology. (2000) 54:914–20. 10.1212/WNL.54.4.914 10690986

[31] Sampson MA Berg AD Huber JN Olgun G. Necessity of intracranial imaging in infants and children with macrocephaly. Pediatr Neurol. (2019) 93:21–6. 10.1016/j.pediatrneurol.2018.10.018 30704866

[32] Iyer A Prabowo A Anink J Spliet WGM Van Rijen PC Aronica E. Cell injury and premature neurodegeneration in focal malformations of cortical development. Brain Pathol. (2014) 24:1–17. 10.1111/bpa.12060 23586324 PMC8029301

[33] Flores-Sarnat L. Hemimegalencephaly: part 1. Genetic, clinical, and imaging aspects. J Child Neurol. (2002) 17:373–84. 10.1177/088307380201700512 12150586

[34] D'Agostino MD Bastos A Piras C Bernasconi A Grisar T Tsur VG. Posterior quadrantic dysplasia or hemi-hemimegalencephaly: a characteristic brain malformation. Neurology. (2004) 62:2214–20. 10.1212/01.WNL.0000130459.91445.91 15210885

[35] Sener RN. MR demonstration of cerebral hemimegalencephaly associated with cerebellar involvement (total hemimegalencephaly). Comput Med Imaging Graph. (1997) 21:201–4. 10.1016/S0895-6111(97)00009-8 9258598

[36] Mirzaa GM Conway RL Gripp KW Lerman-Sagie T Siegel DH De Vries LS. Megalencephaly-capillary malformation (MCAP) and (MPPH) syndromes: two closely related disorders of brain overgrowth and abnormal brain and body morphogenesis. Am J Med Genet A. (2012) 158A:269–91. 10.1002/ajmg.a.34402 22228622

[37] Guerrini R Dobyns WB. Malformations of cortical development: clinical features and genetic causes. Lancet Neurol. (2014) 13:710–26. 10.1016/S1474-4422(14)70040-7 24932993 PMC5548104

[38] D'Gama AM Woodworth MB Hossain AA Bizzotto S Hatem NE LaCoursiere CM. Somatic mutations activating the mTOR pathway in dorsal telencephalic progenitors cause a continuum of cortical dysplasias. Cell Rep. (2017) 21:3754–66. 10.1016/j.celrep.2017.11.106 29281825 PMC5752134

[39] Sandow BA Dory CE Aguiar MA Abuhamad AZ. Best cases from the AFIP. RadioGraphics. (2004) 24:1165–70. 10.1148/rg.244035164 15256635

[40] Ono Y Saito Y Maegaki Y Tohyama J Montassir H Fujii S. Three cases of right frontal megalencephaly: clinical characteristics and long-term outcome. Brain Dev. (2016) 38:302–9. 10.1016/j.braindev.2015.09.005 26415548

[41] Sato N Ota M Yagishita A Miki Y Takahashi T Adachi Y. Aberrant midsagittal fiber tracts in patients with hemimegalencephaly. Am J Neuroradiol. (2008) 29:823–7. 10.3174/ajnr.A0919 18238845 PMC7978186

[42] Takahashi T Sato N Ota M Nakata Y Yamashita F Adachi Y. Asymmetrical interhemispheric fiber tracts in patients with hemimegalencephaly on diffusion tensor magnetic resonance imaging. J Neuroradiol. (2009) 36:249–54. 10.1016/j.neurad.2009.07.005 19783304

[43] Kamiya K Sato N Saito Y Nakata Y Ito K Shigemoto Y. Accelerated myelination along fiber tracts in patients with hemimegalencephaly. J Neuroradiol. (2014) 41:202–10. 10.1016/j.neurad.2013.08.005 24091102

[44] Raybaud C Widjaja E. Development and dysgenesis of the cerebral cortex: malformations of cortical development. Neuroimaging Clin N Am. (2011) 21:483–543. 10.1016/j.nic.2011.05.014 21807310

[45] Barkovich AJ Chuang SH. Unilateral megalencephaly: correlation of MR imaging and pathologic characteristics. Am J Neuroradiol. (1990) 11:523–31. 1693466 PMC8367455

[46] Santos AC Escorsi-Rosset S Simao GN Terra VC Velasco T Neder L. Hemispheric dysplasia and hemimegalencephaly: imaging definitions. Child's Nerv Syst. (2014) 30:1813–21. 10.1007/s00381-014-2476-6 25296542

[47] Re TJ Scarciolla L Takahashi E Specchio N Bernardi B Longo D. Magnetic resonance fiber tracking in a neonate with hemimegalencephaly. J Neuroimaging. (2015) 25:844–7. 10.1111/jon.12206 25655045 PMC4677057

[48] Wiig US Zahl SM Egge A Helseth E Wester K. Epidemiology of benign external hydrocephalus in Norway—a population-based study. Pediatr Neurol. (2017) 73:36–41. 10.1016/j.pediatrneurol.2017.04.018 28666559

[49] Zahl SM Egge A Helseth E Wester K. Clinical, radiological, and demographic details of benign external hydrocephalus: a population-based study. Pediatr Neurol. (2019) 96:53–7. 10.1016/j.pediatrneurol.2019.01.015 30808532

[50] Barlow CF CSF. dynamics in hydrocephalus—with special attention to external hydrocephalus. Brain Dev. (1984) 6:119–27. 10.1016/S0387-7604(84)80060-1 6465466

[51] Zahl SM Egge A Helseth E Wester K. Benign external hydrocephalus: a review, with emphasis on management. Neurosurg Rev. (2011) 34:417–32. 10.1007/s10143-011-0327-4 21647596 PMC3171652

[52] Hellbusch LC. Benign extracerebral fluid collections in infancy: clinical presentation and long-term follow-up. J Neurosurg. (2007) 107:119–25. 10.3171/PED-07/08/119 18459883

[53] Tully HM Dobyns WB. Infantile hydrocephalus: a review of epidemiology, classification and causes. Eur J Med Genet. (2014) 57:359–68. 10.1016/j.ejmg.2014.06.002 24932902 PMC4334358

[54] Kahle KT Kulkarni AV Limbrick DD Warf BC. Hydrocephalus in children. Lancet. (2016) 387:788–99. 10.1016/S0140-6736(15)60694-8 26256071

[55] Lam S Lin Y Cherian J Qadri U Harris DA Melkonian S. Choroid plexus tumors in children: a population-based study. Pediatr Neurosurg. (2013) 49:331–8. 10.1159/000367974 25500637

[56] Kousi M Katsanis N. The genetic basis of hydrocephalus. Annu Rev Neurosci. (2016) 39:409–35. 10.1146/annurev-neuro-070815-014023 27145913

[57] Shaheen R Sebai MA Patel N Ewida N Kurdi W Altweijri I. The genetic landscape of familial congenital hydrocephalus. Ann Neurol. (2017) 81:890–7. 10.1002/ana.24964 28556411

[58] Accogli A Goergen S Izzo G Mankad K Krajden Haratz K Parazzini C. L1CAM variants cause two distinct imaging phenotypes on fetal MRI. Ann Clin Transl Neurol. (2021) 8:2004–12. 10.1002/acn3.51448 34510796 PMC8528460

[59] Kang M Lee YS. The impact of RASopathy-associated mutations on CNS development in mice and humans. Mol Brain. (2019) 12:96. 10.1186/s13041-019-0517-5 31752929 PMC6873535

[60] Renaud DL. Leukoencephalopathies associated with macrocephaly. Semin Neurol. (2012) 32:34–41. 10.1055/s-0032-1306384 22422204

[61] Larson A Goodman S. Glutaric Acidemia Type 1. Seattle, WA: University of Washington(1993). Available online at: http://www.ncbi.nlm.nih.gov/pubmed/31536184 (accessed May 28, 2021). 31536184

[62] Jafari P Braissant O Bonafé L Ballhausen D. The unsolved puzzle of neuropathogenesis in glutaric aciduria type I. Mol Genet Metab. (2011) 104:425–37. 10.1016/j.ymgme.2011.08.027 21944461

[63] Strauss KA Puffenberger EG Robinson DL Morton DH. Type I glutaric aciduria, part 1: natural history of 77 patients. Am J Med Genet Semin Med Genet. (2003) 121 C:38–52. 10.1002/ajmg.c.20007 12888985

[64] Mohammad SA Abdelkhalek HS Ahmed KA Zaki OK. Glutaric aciduria type 1: neuroimaging features with clinical correlation. Pediatr Radiol. (2015) 45:1696–705. 10.1007/s00247-015-3395-8 26111870

[65] Gelener P Severino M Diker S Terali K Tuncel G Tuzlali H. Adult-onset glutaric aciduria type I: rare presentation of a treatable disorder. Neurogenetics. (2020) 21:179–86. 10.1007/s10048-020-00610-9 32306145

[66] Nunes J Loureiro S Carvalho S Pais RP Alfaiate C Faria A. Brain MRI findings as an important diagnostic clue in glutaric aciduria type 1. Neuroradiol J. (2013) 26:155–61. 10.1177/197140091302600204 23859237 PMC5228723

[67] Vester MEM Bilo RAC Karst WA Daams JG Duijst WLJM van Rijn RR. Subdural hematomas: glutaric aciduria type 1 or abusive head trauma? A systematic review. Forensic Sci Med Pathol. (2015) 11:405–15. 10.1007/s12024-015-9698-0 26219480 PMC4529472

[68] Boy N Mühlhausen C Maier EM Heringer J Assmann B Burgard P. Proposed recommendations for diagnosing and managing individuals with glutaric aciduria type I: second revision. J Inherit Metab Dis. (2017) 40:75–101. 10.1007/s10545-016-9999-9 27853989

[69] Kranendijk M Struys EA Salomons GS Van Der Knaap MS Jakobs C. Progress in understanding 2-hydroxyglutaric acidurias. J Inherit Metab Dis. (2012) 35:571–87. 10.1007/s10545-012-9462-5 22391998 PMC3388262

[70] Mühlhausen C Salomons GS Lukacs Z Struys EA van der Knaap MS Ullrich K. Combined D2-/L2-hydroxyglutaric aciduria (SLC25A1 deficiency): clinical course and effects of citrate treatment. J Inherit Metab Dis. (2014) 37:775–81. 10.1007/s10545-014-9702-y 24687295

[71] Steenweg ME Jakobs C Errami A van Dooren SJM Adeva Bartolomé MT Aerssens P. An overview of L-2-hydroxyglutarate dehydrogenase gene (L2HGDH) variants: a genotype-phenotype study. Hum Mutat. (2010) 31:380–90. 10.1002/humu.21197 20052767

[72] D'Incerti L Farina L Moroni I Uziel G Savoiardo M. L-2-Hydroxyglutaric aciduria: MRI in seven cases. Neuroradiology. (1998) 40:727–33. 10.1007/s002340050673 9860123

[73] Moroni I D'Incerti L Farina L Rimoldi M Uziel G. Clinical, biochemical and neuroradiological findings in L-2-hydroxyglutaric aciduria. Neurol Sci. (2000) 21:103–8. 10.1007/s100720070104 10938189

[74] Van Der Knaap MS Jakobs C Hoffmann GF Duran M Muntau AC Schweitzer S. D-2-hydroxyglutaric aciduria: further clinical delineation. J Inherit Metab Dis. (1999) 22:404–13. 10.1023/A:1005548005393 10407777

[75] Galimberti C Madeo A Di Rocco M Fiumara A. Mucopolysaccharidoses: early diagnostic signs in infants and children. Ital J Pediatr. (2018) 44:133. 10.1186/s13052-018-0550-5 30442162 PMC6238260

[76] Nicolas-Jilwan M AlSayed M. Mucopolysaccharidoses: overview of neuroimaging manifestations. Pediatr Radiol. (2018) 48:1503–20. 10.1007/s00247-018-4139-3 29752520

[77] Khan SA Mason RW Giugliani R Orii K Fukao T Suzuki Y. Glycosaminoglycans analysis in blood and urine of patients with mucopolysaccharidosis. Mol Genet Metab. (2018) 125:44–52. 10.1016/j.ymgme.2018.04.011 29779903 PMC6175648

[78] Stapleton M Hoshina H Sawamoto K Kubaski F Mason RW Mackenzie WG. Critical review of current MPS guidelines and management. Mol Genet Metab. (2019) 126:238–45. 10.1016/j.ymgme.2018.07.001 30143438

[79] Sosunov A Olabarria M Goldman JE. Alexander disease: an astrocytopathy that produces a leukodystrophy. Brain Pathol. (2018) 28:388–98. 10.1111/bpa.12601 29740945 PMC8028392

[80] Messing A. Alexander disease. Handb Clin Neurol. (2018) 148:693–700. 10.1016/B978-0-444-64076-5.00044-2 29478608

[81] Busch RM Srivastava S Hogue O Frazier TW Klaas P Hardan A. Neurobehavioral phenotype of autism spectrum disorder associated with germline heterozygous mutations in PTEN. Transl Psychiatry. (2019) 9:253. 10.1038/s41398-019-0588-1 31594918 PMC6783427

[82] Balbi P Salvini S Fundarò C Frazzitta G Maestri R Mosah D. The clinical spectrum of late-onset Alexander disease: a systematic literature review. J Neurol. (2010) 257:1955–62. 10.1007/s00415-010-5706-1 20721574

[83] Van Der Voorn JP Pouwels PJW Salomons GS Barkhof F Van Der Knaap MS. Unraveling pathology in juvenile Alexander disease: serial quantitative MR imaging and spectroscopy of white matter. Neuroradiology. (2009) 51:669–75. 10.1007/s00234-009-0540-9 19484233 PMC2744817

[84] Farina L Pareyson D Minati L Ceccherini I Chiapparini L Romano S. Can MR imaging diagnose adult-onset Alexander disease? Am J Neuroradiol. (2008) 29:1190–6. 10.3174/ajnr.A1060 18388212 PMC8118843

[85] Francis JS Wojtas I Markov V Gray SJ McCown TJ Samulski RJ. N-acetylaspartate supports the energetic demands of developmental myelination via oligodendroglial aspartoacylase. Neurobiol Dis. (2016) 96:323–34. 10.1016/j.nbd.2016.10.001 27717881 PMC5102763

[86] Matalon R Delgado L Michals-Matalon K. Canavan disease. GeneReviews®. (2018). Available online at: https://www.ncbi.nlm.nih.gov/books/NBK1234/ (accessed December 4, 2021).

[87] Brismar J Brismar G Gascon G Ozand P. Canavan disease: CT and MR imaging of the brain. Am J Neuroradiol. (1990) 11:805–10. 2114773 PMC8331616

[88] Israni A V Mandal A. Canavan disease with typical brain MRI and MRS findings. Neurol India. (2017) 65:1191–2. 10.4103/neuroindia.NI_92_17 28879937

[89] Batla A Pandey S Nehru R. Megalencephalic leukoencephalopathy with subcortical cysts: a report of four cases. J Pediatr Neurosci. (2011) 6:74–7. 10.4103/1817-1745.84416 21977097 PMC3173924

[90] Hamilton EMC Tekturk P Cialdella F Van Rappard DiF Wolf NI Yalcinkaya C. Megalencephalic leukoencephalopathy with subcortical cysts: characterization of disease variants. Neurology. (2018) 90:E1395–403. 10.1212/WNL.0000000000005334 29661901 PMC5902784

[91] Hamilton EMC van der Lei HDW Vermeulen G Gerver JAM Lourenço CM Naidu S. Natural history of vanishing white matter. Ann Neurol. (2018) 84:274–88. 10.1002/ana.25287 30014503 PMC6175238

[92] Dooves S Bugiani M Postma NL Polder E Land N Horan ST. Astrocytes are central in the pathomechanisms of vanishing white matter. J Clin Invest. (2016) 126:1512–24. 10.1172/JCI83908 26974157 PMC4811153

[93] Abbink TEM Wisse LE Jaku E Thiecke MJ Voltolini-González D Fritsen H. Vanishing white matter: deregulated integrated stress response as therapy target. Ann Clin Transl Neurol. (2019) 6:1407–22. 10.1002/acn3.50826 31402619 PMC6689685

[94] Van Der Knaap MS Bugiani M Mendes MI Riley LG Smith DEC Rudinger-Thirion J. Biallelic variants in LARS2 and KARS cause deafness and (ovario)leukodystrophy. Neurology. (2019) 92:E1225–37. 10.1212/WNL.0000000000007098 30737337 PMC9281382

[95] Bizzi A Castelli G Bugiani M Barker PB Herskovits EH Danesi U. Classification of childhood white matter disorders using proton MR spectroscopic imaging. Am J Neuroradiol. (2008) 29:1270–5. 10.3174/ajnr.A1106 18483189 PMC2944924

[96] Bugiani M Vuong C Breur M van der Knaap MS. Vanishing white matter: a leukodystrophy due to astrocytic dysfunction. Brain Pathol. (2018) 28:408–21. 10.1111/bpa.12606 29740943 PMC8028328

[97] Crawford DC Acuña JM Sherman SL. FMR1 and the fragile X syndrome: human genome epidemiology review. Genet Med. (2001) 3:359–71. 10.1097/00125817-200109000-00006 11545690 PMC4493892

[98] Hunter JE Berry-Kravis E Hipp H Todd PK. FMR1 Disorders. (1993). Available online at: http://www.ncbi.nlm.nih.gov/pubmed/20301558 (accessed May 28, 2021).

[99] Hagerman PJ Hagerman R. Fragile X syndrome. Curr Biol. (2021) 31:R273–5. 10.1016/j.cub.2021.01.043 33756134

[100] Greenblatt EJ Spradling AC. Fragile X mental retardation 1 gene enhances the translation of large autism-related proteins. Science. (2018) 361:709–12. 10.1126/science.aas9963 30115809 PMC6905618

[101] Yang T Zhao H Lu C Li X Xie Y Fu H. Synaptic plasticity, a prominent contributor to the anxiety in Fragile X Syndrome. Neural Plast. (2016) 2016:9353929. 10.1155/2016/9353929 27239350 PMC4864533

[102] Bagni C Zukin RS. A synaptic perspective of fragile X syndrome and autism spectrum disorders. Neuron. (2019) 101:1070–88. 10.1016/j.neuron.2019.02.041 30897358 PMC9628679

[103] Ciaccio C Fontana L Milani D Tabano S Miozzo M Esposito S. Fragile X syndrome: a review of clinical and molecular diagnoses. Ital J Pediatr. (2017) 43:39. 10.1186/s13052-017-0355-y 28420439 PMC5395755

[104] Borch LA Parboosingh J Thomas MA Veale P. Re-evaluating the first-tier status of fragile X testing in neurodevelopmental disorders. Genet Med. (2020) 22:1036–9. 10.1038/s41436-020-0773-x 32152462

[105] Moeschler JB Shevell M Saul RA Chen E Freedenberg DL Hamid R. Comprehensive evaluation of the child with intellectual disability or global developmental delays. Pediatrics. (2014) 134:e903–18. 10.1542/peds.2014-1839 25157020 PMC9923626

[106] Weinstein V Tanpaiboon P Chapman KA Mew NA Hofherr S. Do the data really support ordering fragile X testing as a first-tier test without clinical features? Genet Med. (2017) 19:1317–22. 10.1038/gim.2017.64 28541279 PMC5702277

[107] Hartley T Potter R Badalato L Smith AC Jarinova O Boycott KM. Fragile X testing as a second-tier test. Genet Med. (2017) 19:1380. 10.1038/gim.2017.147 28914265

[108] Chauhan K Shahrokhi M Huecker MR Vitamin D (2021). Available online at: http://www.ncbi.nlm.nih.gov/pubmed/28722941 (accessed May 28, 2021).

[109] Shore RM Chesney RW. Rickets: part I. Pediatr Radiol. (2013) 43:140–51. 10.1007/s00247-012-2532-x 23208530

[110] Shore RM Chesney RW. Rickets: part II. Pediatr Radiol. (2013) 43:152–72. 10.1007/s00247-012-2536-6 23179485

[111] Aziz Bedair EM Helmy ANE Yakout K Soliman AT. Review of radiologic skeletal changes in Thalassemia. Pediatr Endocrinol Rev. (2008) 6:123–126. 19337165

[112] Pauli RM. Achondroplasia: a comprehensive clinical review. Orphanet J Rare Dis. (2019) 14:1–49. 10.1186/s13023-018-0972-6 30606190 PMC6318916

[113] Manikkam SA Chetcuti K Howell KB Savarirayan R Fink AM Mandelstam SA. Temporal lobe malformations in achondroplasia: expanding the brain imaging phenotype associated with FGFR3-related skeletal dysplasias. Am J Neuroradiol. (2018) 39:380–4. 10.3174/ajnr.A5468 29170271 PMC7410599

[114] Grepl J. Hypochondroplasia. Ces Radiol. (1980) 34:398–406. 7249136

[115] Palagano E Menale C Sobacchi C Villa A. Genetics of osteopetrosis. Curr Osteoporos Rep. (2018) 16:13–25. 10.1007/s11914-018-0415-2 29335834

[116] Penna S Capo V Palagano E Sobacchi C Villa A. One disease, many genes: implications for the treatment of osteopetroses. Front Endocrinol. (2019) 10:85. 10.3389/fendo.2019.00085 30837952 PMC6389615

[117] Singh S Qin C Medarametla S Hegde S V. Craniometaphyseal dysplasia in a 14-month old: a case report and review of imaging differential diagnosis. Radiol Case Reports. (2016) 11:260–5. 10.1016/j.radcr.2016.04.006 27594963 PMC4996902

[118] Nürnberg P Thiele H Chandler D Höhne W Cunningham ML Ritter H. Heterozygous mutations in ANKH, the human ortholog of the mouse progressive ankylosis gene, result in craniometaphyseal dysplasia. Nat Genet. (2001) 28:37–41. 10.1038/ng0501-37 11326272

[119] Hu Y Chen IP de Almeida S Tiziani V Do Amaral CMR Gowrishankar K. A novel autosomal recessive GJA1 missense mutation linked to craniometaphyseal dysplasia. PLoS ONE. (2013) 8:e73576. 10.1371/journal.pone.0073576 23951358 PMC3741164

[120] Jenkins ZA Van Kogelenberg M Morgan T Jeffs A Fukuzawa R Pearl E. Germline mutations in WTX cause a sclerosing skeletal dysplasia but do not predispose to tumorigenesis. Nat Genet. (2009) 41:95–100. 10.1038/ng.270 19079258

[121] Kim SJ Bieganski T Sohn YB Kozlowski K Semënov M Okamoto N. Identification of signal peptide domain SOST mutations in autosomal dominant craniodiaphyseal dysplasia. Hum Genet. (2011) 129:497–502. 10.1007/s00439-011-0947-3 21221996

[122] Pettersson M Vaz R Hammarsjö A Eisfeldt J Carvalho CMB Hofmeister W. Alu-Alu mediated intragenic duplications in IFT81 and MATN3 are associated with skeletal dysplasias. Hum Mutat. (2018) 39:1456–67. 10.1002/humu.23605 30080953

[123] McInerney-Leo AM Schmidts M Cortés CR Leo PJ Gener B Courtney AD. Short-Rib polydactyly and jeune syndromes are caused by mutations in WDR60. Am J Hum Genet. (2013) 93:515–23. 10.1016/j.ajhg.2013.06.022 23910462 PMC3769922

[124] Hellemans J Simon M Dheedene A Alanay Y Mihci E Rifai L. Homozygous inactivating mutations in the NKX3-2 Gene result in spondylo-megaepiphyseal-metaphyseal dysplasia. Am J Hum Genet. (2009) 85:916–22. 10.1016/j.ajhg.2009.11.005 20004766 PMC2790567

[125] Zhang H Yue H Wang C Gu J He J Fu W. Novel mutations in the SEC24D gene in Chinese families with autosomal recessive osteogenesis imperfecta. Osteoporos Int. (2017) 28:1473–80. 10.1007/s00198-016-3866-2 27942778

[126] Kaissi AA Kenis V Shboul M Grill F Ganger R Kircher SG. Tomographic study of the malformation complex in correlation with the genotype in patients with Robinow syndrome: review article. J Investig Med High Impact Case Rep. (2020) 8:2324709620911771. 10.1177/2324709620911771 32172608 PMC7074505

[127] Bacino CA. ROR2-Related Robinow Syndrome. Seattle, WA: University of Washington (1993). Available online at: http://www.ncbi.nlm.nih.gov/pubmed/20301418 (accessed May 28, 2021). 20301418

[128] Roifman M Brunner H Lohr J Mazzeu J Chitayat D. Autosomal dominant robinow syndrome. In: Adam MP Ardinger HH Pagon RA Wallace SE Bean LJH Gripp KW Mirzaa GM Amemiya A editors. Definitions. Seattle, WA: University of Washington. 10.32388/7nstn5 25577943

[129] Miller DT Freedenberg D Schorry E Ullrich NJ Viskochil D Korf BR. Health supervision for children with neurofibromatosis type 1. Pediatrics. (2019) 143:e20190660. 10.1542/peds.2019-0660 31010905

[130] Friedman J. Neurofibromatosis 1. Pagon RA, Adam MP, Ardinger HH. GeneReviews® [Internet]. Seattle, WA: University of Washington (1998). Available online at: https://www.ncbi.nlm.nih.gov/books/NBK1109/ (accessed May 28, 2021).

[131] Evans DGR Salvador H Chang VY Erez A Voss SD Schneider KW. Cancer and central nervous system tumor surveillance in pediatric neurofibromatosis 1. Clin Cancer Res. (2017) 23:e46–53. 10.1158/1078-0432.CCR-17-0589 28620004

[132] Monroe CL Dahiya S Gutmann DH. Dissecting clinical heterogeneity in neurofibromatosis type 1. Annu Rev Pathol Mech Dis. (2017) 12:53–74. 10.1146/annurev-pathol-052016-100228 28135565

[133] Tadini G Milani D Menni F Pezzani L Sabatini C Esposito S. Is it time to change the neurofibromatosis 1 diagnostic criteria? Eur J Intern Med. (2014) 25:506–10. 10.1016/j.ejim.2014.04.004 24784952

[134] Vogel AC Gutmann DH Morris SM. Neurodevelopmental disorders in children with neurofibromatosis type 1. Dev Med Child Neurol. (2017) 59:1112–6. 10.1111/dmcn.13526 28845518

[135] Gutmann DH Ferner RE Listernick RH Korf BR Wolters PL Johnson KJ. Neurofibromatosis type 1. Nat Rev Dis Prim 2017 31. (2017) 3:1–17. 10.1038/nrdp.2017.4 28230061

[136] Cusmai R Curatolo P Mangano S Cheminal R Echenne B. Hemimegalencephaly and neurofibromatosis. Neuropediatrics. (1990) 21:179–82. 10.1055/s-2008-1071490 2127080

[137] Balestri P Vivarelli R Grosso S Santori L Farnetani MA Galluzzi P. Malformations of cortical development in neurofibromatosis type 1. Neurology. (2003) 61:1799–801. 10.1212/01.WNL.0000099080.90726.BA 14694053

[138] Ruggieri M Mastrangelo M Spalice A Mariani R Torrente I Polizzi A. Bilateral (opercular and paracentral lobular) polymicrogyria and neurofibromatosis type 1. Am J Med Genet A. (2011) 155:582–5. 10.1002/ajmg.a.33318 21344624

[139] Brems H Pasmant E Van Minkelen R Wimmer K Upadhyaya M Legius E. Review and update of SPRED1 mutations causing legius syndrome. Hum Mutat. (2012) 33:1538–46. 10.1002/humu.22152 22753041

[140] Henske EP Józwiak S Kingswood JC Sampson JR Thiele EA. Tuberous sclerosis complex. Nat Rev Dis Prim. (2016) 2:16035. 10.1038/nrdp.2016.35 27226234

[141] Krueger DA Northrup H Krueger DA Roberds S Smith K Sampson J. Tuberous sclerosis complex surveillance and management: recommendations of the 2012 international tuberous sclerosis complex consensus conference. Pediatr Neurol. (2013) 49:255–65. 10.1016/j.pediatrneurol.2013.08.002 24053983 PMC4058297

[142] Islam MP Roach ES. Tuberous sclerosis complex. Handb Clin Neurol. 132:97–109. 10.1016/B978-0-444-62702-5.00006-8 26564073

[143] Northrup H Krueger DA Roberds S Smith K Sampson J Korf B. Tuberous sclerosis complex diagnostic criteria update: recommendations of the 2012 international tuberous sclerosis complex consensus conference. Pediatr Neurol. (2013) 49:243–54. 10.1016/j.pediatrneurol.2013.08.001 24053982 PMC4080684

[144] Lu DS Karas PJ Krueger DA Weiner HL. Central nervous system manifestations of tuberous sclerosis complex. Am J Med Genet C Semin Med Genet. (2018) 178:291–8. 10.1002/ajmg.c.31647 30230171

[145] Galluzzi PP Cerase A Strambi M Buoni S Fois A Venturi C. Hemimegalencephaly in tuberous sclerosis complex. J Child Neurol. (2002) 17:677–80. 10.1177/088307380201700905 12503644

[146] Smalley SL Tanguay PE Smith M Gutierrez G. Autism and tuberous sclerosis. J Autism Dev Disord. (1992) 22:339–55. 10.1007/BF01048239 1400103

[147] Sakuma H Iwata O Sasaki M. Longitudinal MR findings in a patient with hemimegalencephaly associated with tuberous sclerosis. Brain Dev. (2005) 27:458–61. 10.1016/j.braindev.2004.11.004 16122638

[148] Guerra MP Cavalleri F Migone N Lugli L Delalande O Cavazzuti GB. Intractable epilepsy in hemimegalencephaly and tuberous sclerosis complex. J Child Neurol. (2007) 22:80–4. 10.1177/0883073807299960 17608312

[149] Cuddapah VA Thompson M Blount J Li R Guleria S Goyal M. Hemispherectomy for hemimegalencephaly due to tuberous sclerosis and a review of the literature. Pediatr Neurol. (2015) 53:452–5. 10.1016/j.pediatrneurol.2015.06.020 26231267

[150] Baron Y Barkovich AJ. MR imaging of tuberous sclerosis in neonates and young infants. Am J Neuroradiol. (1999) 20:907–16. 10369365 PMC7056154

[151] Chu-Shore CJ Frosch MP Grant PE Thiele EA. Progressive multifocal cystlike cortical tubers in tuberous sclerosis complex: clinical and neuropathologic findings. Epilepsia. (2009) 50:2648–51. 10.1111/j.1528-1167.2009.02193.x 19624715

[152] Martí-Bonmatí L Menor F Dosdá R. Tuberous sclerosis: differences between cerebral and cerebellar cortical tubers in a pediatric population. Am J Neuroradiol. (2000) 21:557–60. 10730651 PMC8175001

[153] Daghistani R Rutka J Widjaja E MRI. characteristics of cerebellar tubers and their longitudinal changes in children with tuberous sclerosis complex. Child's Nerv Syst. (2015) 31:109–13. 10.1007/s00381-014-2542-0 25200047

[154] Boronat S Thiele EA Caruso P. Cerebellar lesions are associated with TSC2 mutations in tuberous sclerosis complex: a retrospective record review study. Dev Med Child Neurol. (2017) 59:1071–6. 10.1111/dmcn.13499 28786492

[155] Manara R Bugin S Pelizza MF Sartori S Nosadini M Labriola F. Genetic and imaging features of cerebellar abnormalities in tuberous sclerosis complex: more insights into their pathogenesis. Dev Med Child Neurol. (2018) 60:724–5. 10.1111/dmcn.13769 29882962

[156] Akbari M Chen H Guo G Legan Z Ghali G. Basal cell nevus syndrome (Gorlin syndrome): genetic insights, diagnostic challenges, and unmet milestones. Pathophysiology. (2018) 25:77–82. 10.1016/j.pathophys.2017.12.004 29454489

[157] Altaraihi M Wadt K Ek J Gerdes AM Ostergaard E. A healthy individual with a homozygous PTCH2 frameshift variant: are variants of PTCH2 associated with nevoid basal cell carcinoma syndrome? Hum Genome Var. (2019) 6:10. 10.1038/s41439-019-0041-2 30820324 PMC6384928

[158] Fan Z Li J Du J Zhang H Shen Y Wang CY. A missense mutation in PTCH2 underlies dominantly inherited NBCCS in a Chinese family. J Med Genet. (2008) 45:303–8. 10.1136/jmg.2007.055343 18285427

[159] Thalakoti S Geller T. Basal cell nevus syndrome or Gorlin syndrome. Handb Clin Neurol. 132:119–28. 10.1016/B978-0-444-62702-5.00008-1 26564075

[160] Shiohama T Fujii K Miyashita T Mizuochi H Uchikawa H Shimojo N. Brain morphology in children with nevoid basal cell carcinoma syndrome. Am J Med Genet A. (2017) 173:946–52. 10.1002/ajmg.a.38115 28328116

[161] Northcott PA Korshunov A Witt H Hielscher T Eberhart CG Mack S. Medulloblastoma comprises four distinct molecular variants. J Clin Oncol. (2011) 29:1408–14. 10.1200/JCO.2009.27.4324 20823417 PMC4874239

[162] Evans DG Oudit D Smith MJ Rutkowski D Allan E Newman WG. First evidence of genotype-phenotype correlations in Gorlin syndrome. J Med Genet. (2017) 54:530–6. 10.1136/jmedgenet-2017-104669 28596197

[163] Guerrini-Rousseau L Dufour C Varlet P Masliah-Planchon J Bourdeaut F Guillaud-Bataille M. Germline SUFU mutation carriers and medulloblastoma: clinical characteristics, cancer risk, and prognosis. Neuro Oncol. (2018) 20:1122–32. 10.1093/neuonc/nox228 29186568 PMC6280147

[164] Kimonis VE Mehta SG DiGiovanna JJ Bale SJ Pastakia B. Radiological features in 82 patients with nevoid basal cell carcinoma (NBCC or Gorlin) syndrome. Genet Med. (2004) 6:495–502. 10.1097/01.GIM.0000145045.17711.1C 15545745

[165] Jones EA Sajid MI Shenton A Evans DG. Basal cell carcinomas in Gorlin syndrome: a review of 202 patients. J Skin Cancer. (2011) 2011:217378. 10.1155/2011/217378 21152126 PMC2998699

[166] Kimonis VE Singh KE Zhong R Pastakia B Digiovanna JJ Bale SJ. Clinical and radiological features in young individuals with nevoid basal cell carcinoma syndrome. Genet Med. (2013) 15:79–83. 10.1038/gim.2012.96 22918513

[167] Neylon OM Werther GA Sabin MA. Overgrowth syndromes. Curr Opin Pediatr. (2012) 24:505–11. 10.1097/MOP.0b013e3283558995 22705997

[168] Tatton-Brown K Weksberg R. Molecular mechanisms of childhood overgrowth. Am J Med Genet Part C Semin Med Genet. (2013) 163:71–5. 10.1002/ajmg.c.31362 23606607

[169] Verge CF Mowat D. Overgrowth. Arch Dis Child. (2010) 95:458–63. 10.1136/adc.2009.157693 20371592

[170] Brioude F Toutain A Giabicani E Cottereau E Cormier-Daire V Netchine I. Overgrowth syndromes — clinical and molecular aspects and tumour risk. Nat Rev Endocrinol. (2019) 15:299–311. 10.1038/s41574-019-0180-z 30842651

[171] Saxton RA Sabatini DM. mTOR Signaling in growth, metabolism, and disease. Cell. (2017) 168:960–76. 10.1016/j.cell.2017.02.004 28283069 PMC5394987

[172] Rivière JB Mirzaa GM O'Roak BJ Beddaoui M Alcantara D Conway RL. *De novo* germline and postzygotic mutations in AKT3, PIK3R2 and PIK3CA cause a spectrum of related megalencephaly syndromes. Nat Genet. (2012) 44:934–40. 10.1038/ng.2331 22729224 PMC3408813

[173] Jansen LA Mirzaa GM Ishak GE O'Roak BJ Hiatt JB Roden WH. PI3K/AKT pathway mutations cause a spectrum of brain malformations from megalencephaly to focal cortical dysplasia. Brain. (2015) 138:1613–28. 10.1093/brain/awv045 25722288 PMC4614119

[174] Huang J Manning BD. The TSC1-TSC2 complex: a molecular switchboard controlling cell growth. Biochem J. (2008) 412:179–90. 10.1042/BJ20080281 18466115 PMC2735030

[175] Iffland PH Crino PB. Focal cortical dysplasia: gene mutations, cell signaling, and therapeutic implications. Annu Rev Pathol Mech Dis. (2017) 12:547–71. 10.1146/annurev-pathol-052016-100138 28135561

[176] Mirzaa GM Rivière JB Dobyns WB. Megalencephaly syndromes and activating mutations in the PI3K-AKT pathway: MPPH and MCAP. Am J Med Genet C Semin Med Genet. (2013) 163:122–30. 10.1002/ajmg.c.31361 23592320

[177] Loconte DC Grossi V Bozzao C Forte G Bagnulo R Stella A. Molecular and functional characterization of three different postzygotic mutations in PIK3CA-related overgrowth spectrum (PROS) patients: effects on PI3K/AKT/mTOR signaling and sensitivity to PIK3 inhibitors. PLoS ONE. (2015) 10:e0123092. 10.1371/journal.pone.0123092 25915946 PMC4411002

[178] Keppler-Noreuil KM Sapp JC Lindhurst MJ Parker VER Blumhorst C Darling T. Clinical delineation and natural history of the PIK3CA-related overgrowth spectrum. Am J Med Genet A. (2014) 164:1713–33. 10.1002/ajmg.a.36552 24782230 PMC4320693

[179] Dobyns WB Mirzaa GM. Megalencephaly syndromes associated with mutations of core components of the PI3K-AKT–MTOR pathway: PIK3CA, PIK3R2, AKT3, and MTOR. Am J Med Genet C Semin Med Genet. (2019) 181:582–90. 10.1002/ajmg.c.31736 31441589

[180] Vahidnezhad H Youssefian L Uitto J. Klippel-Trenaunay syndrome belongs to the PIK3CA-related overgrowth spectrum (PROS). Exp Dermatol. (2016) 25:17–9. 10.1111/exd.12826 26268729

[181] Yeung KS Ip JJK Chow CP Kuong EYL Tam PKH Chan GCF. Somatic PIK3CA mutations in seven patients with PIK3CA-related overgrowth spectrum. Am J Med Genet A. (2017) 173:978–84. 10.1002/ajmg.a.38105 28328134

[182] Mirzaa G Timms AE Conti V Boyle EA Girisha KM Martin B. PIK3CA-associated developmental disorders exhibit distinct classes of mutations with variable expression and tissue distribution. JCI Insight. (2016) 1:e87623. 10.1172/JCI.INSIGHT.87623 27631024 PMC5019182

[183] Clayton-Smith J Kerr B Brunner H Tranebjaerg L Magee A Hennekam RCM. Macrocephaly with cutis marmorata, haemangioma and syndactyly - a distinctive overgrowth syndrome. Clin Dysmorphol. (1997) 6:291–302. 10.1097/00019605-199710000-00001 9354837

[184] Conway RL Pressman BD Dobyns WB Danielpour M Lee J Sanchez-Lara PA. Neuroimaging findings in macrocephaly-capillary malformation: a longitudinal study of 17 patients. Amer J Med Genet A 143A:2981–3008. 10.1002/ajmg.a.32040 18000912 PMC6816457

[185] Sapp JC Turner JT Van De Kamp JM Van Dijk FS Lowry RB Biesecker LG. Newly delineated syndrome of congenital lipomatous overgrowth, vascular malformations, and epidermal nevi (CLOVE syndrome) in seven patients. Amer J Med Genet A 143A:2944–58. 10.1002/ajmg.a.32023 17963221

[186] Alomari AI. Characterization of a distinct syndrome that associates complex truncal overgrowth, vascular, and acral anomalies: a descriptive study of 18 cases of CLOVES syndrome. Clin Dysmorphol. (2009) 18:1–7. 10.1097/MCD.0b013e328317a716 19011570

[187] Martinez-Lopez A Blasco-Morente G Perez-Lopez I Herrera-Garcia JD Luque-Valenzuela M Sanchez-Cano D. CLOVES syndrome: review of a PIK3CA-related overgrowth spectrum (PROS). Clin Genet. 91:14–21 (2017). 10.1111/cge.12832 27426476

[188] Gucev ZS Tasic V Jancevska A Konstantinova MK Pop-Jordanova N Trajkovski Z. Congenital lipomatous overgrowth, vascular malformations, and epidermal nevi (CLOVE) syndrome: CNS malformations and seizures may be a component of this disorder. Am J Med Genet A. (2008) 146:2688–90. 10.1002/ajmg.a.32515 18816642 PMC2819374

[189] Alomari AI Chaudry G Rodesch G Burrows PE Mulliken JB Smith ER. Complex spinal-paraspinal fast-flow lesions in CLOVES syndrome: analysis of clinical and imaging findings in 6 patients. Am J Neuroradiol. (2011) 32:1812–7. 10.3174/ajnr.A2349 21310861 PMC7966001

[190] Luks VL Kamitaki N Vivero MP Uller W Rab R Bovee JVMG. Lymphatic and other vascular malformative/overgrowth disorders are caused by somatic mutations in PIK3CA. J Pediatr. (2015) 166:1048.e5–54.e5. 10.1016/j.jpeds.2014.12.069 25681199 PMC4498659

[191] Parkes WF. Haemangiectatic hypertrophies of the foot and lower extremity. Med Press. (1908) 136:261.

[192] Torregrosa A Martí-Bonmatí L Higueras V Poyatos C Sanchís A. Klippel-Trenaunay syndrome: frequency of cerebral and cerebellar hemihypertrophy on MRI. Neuroradiology. (2000) 42:420–3. 10.1007/s002340000310 10929301

[193] Vurucu S Battal B Kocaoglu M Akin R. Klippel-Trenaunay syndrome with hemimegalencephaly, retroperitoneal lymphangioma and double inferior vena cava. Br J Radiol. (2009) 82:e102–4. 10.1259/bjr/36297676 19386952

[194] Pichierri A Piccirilli M Passacantilli E Frati A Santoro A. Klippel-Trenaunay-Weber syndrome and intramedullary cervical cavernoma: a very rare association. Case report Surg Neurol. (2006) 66:203–6. 10.1016/j.surneu.2005.11.062 16876633

[195] Boutarbouch M Salem D. Ben, Giré L, Giroud M, Béjot Y, Ricolfi F. Multiple cerebral and spinal cord cavernomas in Klippel-Trenaunay-Weber syndrome. J Clin Neurosci. (2010) 17:1073–5. 10.1016/j.jocn.2009.11.013 20493710

[196] Kim YW Kim N Hwang JM Choung HK Khwarg SI. Teaching neuroimages: multiple giant intracranial aneurysms in Klippel-Trenaunay syndrome. Neurology. (2013) 81:e17–8. 10.1212/WNL.0b013e31829bfd4c 23858416

[197] Fukaya R Yanagisawa K Fukuchi M Fujii K. Posterior cerebral artery giant aneurysm associated with bilateral internal carotid artery occlusion in a Klippel-Trenaunay syndrome patient: a case report. Br J Neurosurg. (2019) 33:591–3. 10.1080/02688697.2017.1394446 29069941

[198] Sakai K Sibazaki K Kimura K Kobayashi K Matsumoto N Iguchi Y. Paradoxical brain embolism with Klippel-Trenaunay Syndrome. Intern Med. (2011) 50:141–3. 10.2169/internalmedicine.50.3870 21245639

[199] Sethi D Kingwill A Myakova N. Proteus syndrome. Anasthesiol Intensivmed. (2018) 59:S85–92. 10.19224/ai2018.S85

[200] Dietrich RB Glidden DE Roth GM Martin RA Demo DS. The proteus syndrome: CNS manifestations. Am J Neuroradiol. (1998) 19:987–90. 9613526 PMC8337570

[201] Delone DR Brown WD Gentry LR. Proteus syndrome: craniofacial and cerebral MRI. Neuroradiology. (1999) 41:840–3. 10.1007/s002340050853 10602859

[202] Cohen MM. Proteus syndrome: an update. Am J Med Genet Semin Med Genet. (2005) 137C:38–52. 10.1002/ajmg.c.30063 16010681

[203] Anik Y Anik I Gonullu E Inan N Demirci A. Proteus syndrome with syringohydromyelia and arachnoid cyst. Child's Nerv Syst. (2007) 23:1199–202. 10.1007/s00381-007-0364-z 17593376

[204] Cohen MM. Proteus syndrome review: molecular, clinical, and pathologic features. Clin Genet. (2014) 85:111–9. 10.1111/cge.12266 23992099

[205] Gilbert-Barness E Cohen MM Opitz JM. Multiple meningiomas, craniofacial hyperostosis and retinal abnormalities in Proteus syndrome. Am J Med Genet. (2000) 93:234–40. 10.1002/1096-8628(20000731)93:3<234::AID-AJMG15>3.0.CO;2-9 10925389

[206] Edmondson A Kalish J. Overgrowth syndromes. J Pediatr Genet. (2015) 04:136–43. 10.1055/s-0035-1564440 27617124 PMC4918719

[207] Wang KH Kupa J Duffy KA Kalish JM. Diagnosis and management of Beckwith-Wiedemann Syndrome. Front Pediatr. (2020) 7:562. 10.3389/fped.2019.00562 32039119 PMC6990127

[208] Weksberg R Shuman C Beckwith JB. Beckwith-Wiedemann syndrome. Eur J Hum Genet. (2010) 18:8–14. 10.1038/ejhg.2009.106 19550435 PMC2987155

[209] Brioude F Kalish JM Mussa A Foster AC Bliek J Ferrero GB. Clinical and molecular diagnosis, screening and management of Beckwith-Wiedemann syndrome: an international consensus statement. Nat Rev Endocrinol. (2018) 14:229–49. 10.1038/nrendo.2017.166 29377879 PMC6022848

[210] Gardiner K Chitayat D Choufani S Shuman C Blaser S Terespolsky D. Brain abnormalities in patients with Beckwith-Wiedemann syndrome. Am J Med Genet A. (2012) 158A:1388–94. 10.1002/ajmg.a.35358 22585446

[211] Tatton-Brown K Rahman N. Sotos syndrome. Eur J Hum Genet. (2007) 15:264–71. 10.1038/sj.ejhg.5201686 16969376

[212] Foster A Zachariou A Loveday C Ashraf T Blair E Clayton-Smith J. The phenotype of Sotos syndrome in adulthood: a review of 44 individuals. Am J Med Genet C Semin Med Genet. (2019) 181:502–8. 10.1002/ajmg.c.31738 31479583

[213] Lane C Milne E Freeth M. Cognition and behaviour in Sotos syndrome: a systematic review. PLoS ONE. (2016) 11:e149189. 10.1371/journal.pone.0149189 26872390 PMC4752321

[214] Al-Mulla N Belgaumi AF Teebi A. Cancer in Sotos syndrome: report of a patient with acute myelocytic leukemia and review of the literature. J Pediatr Hematol Oncol. (2004) 26:204–8. 10.1097/00043426-200403000-00013 15125616

[215] Schaefer GB Bodensteiner JB Buehler BA Lin A Cole TRP. The neuroimaging findings in Sotos syndrome. Am J Med Genet. (1997) 68:462–5. 9021022 10.1002/(sici)1096-8628(19970211)68:4<462::aid-ajmg18>3.0.co;2-q

[216] Horikoshi H Kato Z. Neuroradiologic findings in Sotos syndrome. J Child Neurol. (2006) 21:614–8. 10.1177/08830738060210071001 16970856

[217] Klaassens M Morrogh D Rosser EM Jaffer F Vreeburg M Bok LA. Malan syndrome: Sotos-like overgrowth with *de novo* NFIX sequence variants and deletions in six new patients and a review of the literature. Eur J Hum Genet. (2015) 23:610–5. 10.1038/ejhg.2014.162 25118028 PMC4402637

[218] Priolo M Schanze D Tatton-Brown K Mulder PA Tenorio J Kooblall K. Further delineation of Malan syndrome. Hum Mutat. (2018) 39:1226–37. 10.1002/humu.23563 29897170 PMC6175110

[219] Tatton-Brown K Murray A Hanks S Douglas J Armstrong R Banka S. Weaver syndrome and EZH2 mutations: clarifying the clinical phenotype. Am J Med Genet A. (2013) 161:2972–80. 10.1002/ajmg.a.36229 24214728

[220] Tatton-Brown K Rahman N. EZH2-Related Overgrowth. (1993). Available online at: http://www.ncbi.nlm.nih.gov/pubmed/23865096 (accessed May 28, 2021).

[221] Tatton-Brown K Rahman N. The NSD1 and EZH2 overgrowth genes, similarities and differences. Am J Med Genet C Semin Med Genet. (2013) 163:86–91. 10.1002/ajmg.c.31359 23592277 PMC4845886

[222] Hamosh A Freeman BM Hoon J Breiter SN. Pachygyria in weaver syndrome [1]. Am J Med Genet. (1999) 86:395–7. 10.1002/(SICI)1096-8628(19991008)86:4<395::AID-AJMG16>3.0.CO;2-L 10494098

[223] Al-Salem A Alshammari MJ Hassan H Alazami AM Alkuraya FS. Weaver syndrome and defective cortical development: a rare association. Am J Med Genet Part A. (2013) 161:225–7. 10.1002/ajmg.a.35660 23239504

[224] Basel-Vanagaite L. Acute lymphoblastic leukemia in weaver syndrome. Am J Med Genet A. (2010) 152:383–6. 10.1002/ajmg.a.33244 20101679

[225] Coulter D Powell CM Gold S. Weaver syndrome and neuroblastoma. J Pediatr Hematol Oncol. (2008) 30:758–60. 10.1097/MPH.0b013e3181758974 19011474

[226] Villani A Greer MLC Kalish JM Nakagawara A Nathanson KL Pajtler KW. Recommendations for cancer surveillance in individuals with RASopathies and other rare genetic conditions with increased cancer risk. Clin Cancer Res. (2017) 23:e83–90. 10.1158/1078-0432.CCR-17-0631 28620009

[227] Hansen-Kiss E Beinkampen S Adler B Frazier T Prior T Erdman S. retrospective chart review of the features of PTEN hamartoma tumour syndrome in children. J Med Genet. (2017) 54:471–8. 10.1136/jmedgenet-2016-104484 28526761

[228] Tan MH Eng C RE. Cowden syndrome and PTEN hamartoma tumor syndrome: systematic review and revised diagnostic criteria. J Natl Cancer Inst. (2014) 106:1607–16. 10.1093/jnci/dju130 24899679 PMC6937005

[229] Yehia L Keel E Eng C. The Clinical Spectrum of PTEN Mutations. Annu Rev Med. (2020) 71:103–16. 10.1146/annurev-med-052218-125823 31433956

[230] Macken WL Tischkowitz M Lachlan KL PTEN PTEN Hamartoma tumor syndrome in childhood: a review of the clinical literature. Am J Med Genet C Semin Med Genet. (2019) 181:591–610. 10.1002/ajmg.c.31743 31609537

[231] Ciaccio C Saletti V D'Arrigo S Esposito S Alfei E Moroni I. Clinical spectrum of PTEN mutation in pediatric patients. A bicenter experience. Eur J Med Genet. (2019) 62:10596. 10.1016/j.ejmg.2018.12.001 30528446

[232] Mester JL Tilot AK Rybicki LA Frazier TW Eng C. Analysis of prevalence and degree of macrocephaly in patients with germline PTEN mutations and of brain weight in Pten knock-in murine model. Eur J Hum Genet. (2011) 19:763–8. 10.1038/ejhg.2011.20 21343951 PMC3137495

[233] Plamper M Gohlke B Schreiner F Woelfle J. Phenotype-driven diagnostic of PTEN hamartoma tumor syndrome: macrocephaly, but neither height nor weight development, is the important trait in children. Cancers (Basel). (2019) 11:975. 10.3390/cancers11070975 31336731 PMC6679020

[234] Elia M Amato C Bottitta M Grillo L Calabrese G Esposito M. An atypical patient with Cowden syndrome and PTEN gene mutation presenting with cortical malformation and focal epilepsy. Brain Dev. (2012) 34:873–6. 10.1016/j.braindev.2012.03.005 22469695

[235] O'Rourke DJ Twomey E Lynch SA King MD. Cortical dysplasia associated with the PTEN mutation in Bannayan Riley Ruvalcaba syndrome: a rare finding. Clin Dysmorphol. (2012) 21:91–2. 10.1097/MCD.0b013e328351639d 22327138

[236] Dhamija R Weindling SM Porter AB Hu LS Wood CP Hoxworth JM. Neuroimaging abnormalities in patients with Cowden syndrome: retrospective single-center study. Neurol Clin Pract. (2018) 8:207–13. 10.1212/CPJ.0000000000000463 30105160 PMC6075984

[237] Ghusayni R Sachdev M Gallentine W Mikati MA McDonald MT. Hemimegalencephaly with Bannayan-Riley-Ruvalcaba syndrome. Epileptic Disord. (2018) 20:30–4. 10.1684/epd.2018.0954 29444762

[238] Vanderver A Tonduti D Kahn I Schmidt J Medne L Vento J. Characteristic brain magnetic resonance imaging pattern in patients with macrocephaly and PTEN mutations. Am J Med Genet A. (2014) 164:627–33. 10.1002/ajmg.a.36309 24375884 PMC5234041

[239] Tan MH Mester J Peterson C Yang Y Chen JL Rybicki LA. A clinical scoring system for selection of patients for pten mutation testing is proposed on the basis of a prospective study of 3042 probands. Am J Hum Genet. (2011) 88:42–56. 10.1016/j.ajhg.2010.11.013 21194675 PMC3014373

[240] Mirzaa GM Conti V Timms AE Smyser CD Ahmed S Carter M. Characterisation of mutations of the phosphoinositide-3-kinase regulatory subunit, PIK3R2, in perisylvian polymicrogyria: a next-generation sequencing study. Lancet Neurol. (2015) 14:1182–95. 10.1016/S1474-4422(15)00278-1 26520804 PMC4672724

[241] Mirzaa GM Campbell CD Solovieff N Goold CP Jansen LA Menon S. Association of MTOR mutations with developmental brain disorders, including megalencephaly, focal cortical dysplasia, and pigmentary mosaicism. JAMA Neurol. (2016) 73:836–45. 10.1001/jamaneurol.2016.0363 27159400 PMC4979321

[242] Alcantara D Timms AE Gripp K Baker L Park K Collins S. Mutations of AKT3 are associated with a wide spectrum of developmental disorders including extreme megalencephaly. Brain. (2017) 140:2610–22. 10.1093/brain/awx203 28969385 PMC6080423

[243] Lee JH Huynh M Silhavy JL Kim S Dixon-Salazar T Heiberg A. *De novo* somatic mutations in components of the PI3K-AKT3-mTOR pathway cause hemimegalencephaly. Nat Genet. (2012) 44:941–5. 10.1038/ng.2329 22729223 PMC4417942

[244] Concolino D Deodato F Parini R. Enzyme replacement therapy: efficacy and limitations. Ital J Pediatr. (2018) 44:120. 10.1186/s13052-018-0562-1 30442189 PMC6238252

[245] Chen HH Sawamoto K Mason RW Kobayashi H Yamaguchi S Suzuki Y. Enzyme replacement therapy for mucopolysaccharidoses; past, present, and future. J Hum Genet. (2019) 64:1153–71. 10.1038/s10038-019-0662-9 31455839

[246] Squeri G Passerini L Ferro F Laudisa C Tomasoni D Deodato F. Targeting a pre-existing anti-transgene t cell response for effective gene therapy of MPS-I in the mouse model of the disease. Mol Ther. (2019) 27:1215–27. 10.1016/j.ymthe.2019.04.014 31060789 PMC6612662

[247] Wood H. Neurodevelopmental disorders: gene therapy for mucopolysaccharidosis shows promise. Nat Rev Neurol. (2017) 13:512–3. 10.1038/nrneurol.2017.110 28752858

[248] Mithal DS Rubin JP. A promising small molecule for vanishing white matter disease. Pediatr Neurol Briefs. (2018) 32:5. 10.15844/pedneurbriefs-32-5 30174381 PMC6115330

[249] Wong YL Lebon L Edalji R Lim H Ben Sun C Sidrauski C. The small molecule ISRIB rescues the stability and activity of vanishing white matter disease eIF2B mutant complexes. Elife. (2018) 7:e32733. 10.7554/eLife.32733 29489452 PMC5829914

[250] Curatolo P Franz DN Lawson JA Yapici Z Ikeda H Polster T. Adjunctive everolimus for children and adolescents with treatment-refractory seizures associated with tuberous sclerosis complex: *post-hoc* analysis of the phase 3 EXIST-3 trial. Lancet Child Adolesc Heal. (2018) 2:495–504. 10.1016/S2352-4642(18)30099-3 30169322

[251] Zeng LH Xu L Gutmann DH Wong M. Rapamycin prevents epilepsy in a mouse model of tuberous sclerosis complex. Ann Neurol. (2008) 63:444–53. 10.1002/ana.21331 18389497 PMC3937593

[252] Hagemann TL Powers B Mazur C Kim A Wheeler S Hung G. Antisense suppression of glial fibrillary acidic protein as a treatment for Alexander disease. Ann Neurol. (2018) 83:27–39. 10.1002/ana.25118 29226998 PMC5876100

