# Supplementary figures and images for: Diagnostic Approach to Macrocephaly in Children
Source: Front Pediatr. 2022 Jan 14;9:794069. doi: 10.3389/fped.2021.794069 (PMC8795981; doi:10.3389/fped.2021.794069)

# Exclusion of Secondary Causes of Macrocrania

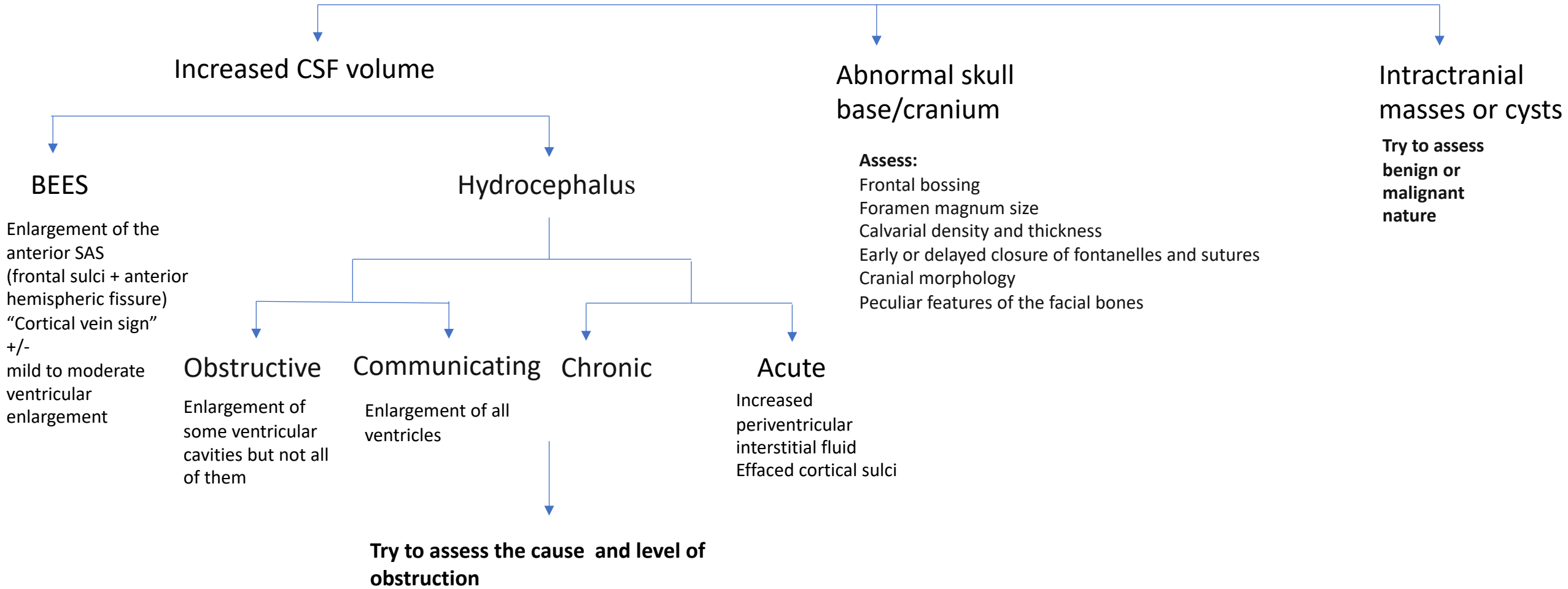

Supplement: Supplementary Figure 1 — Imaging approach to secondary causes of macrocrania. BEES, Benign enlargement of subarachnoid spaces in infancy; CSF, cerebrospinal fluid; SAS, subarachnoid spaces. [file Image_1.pdf]

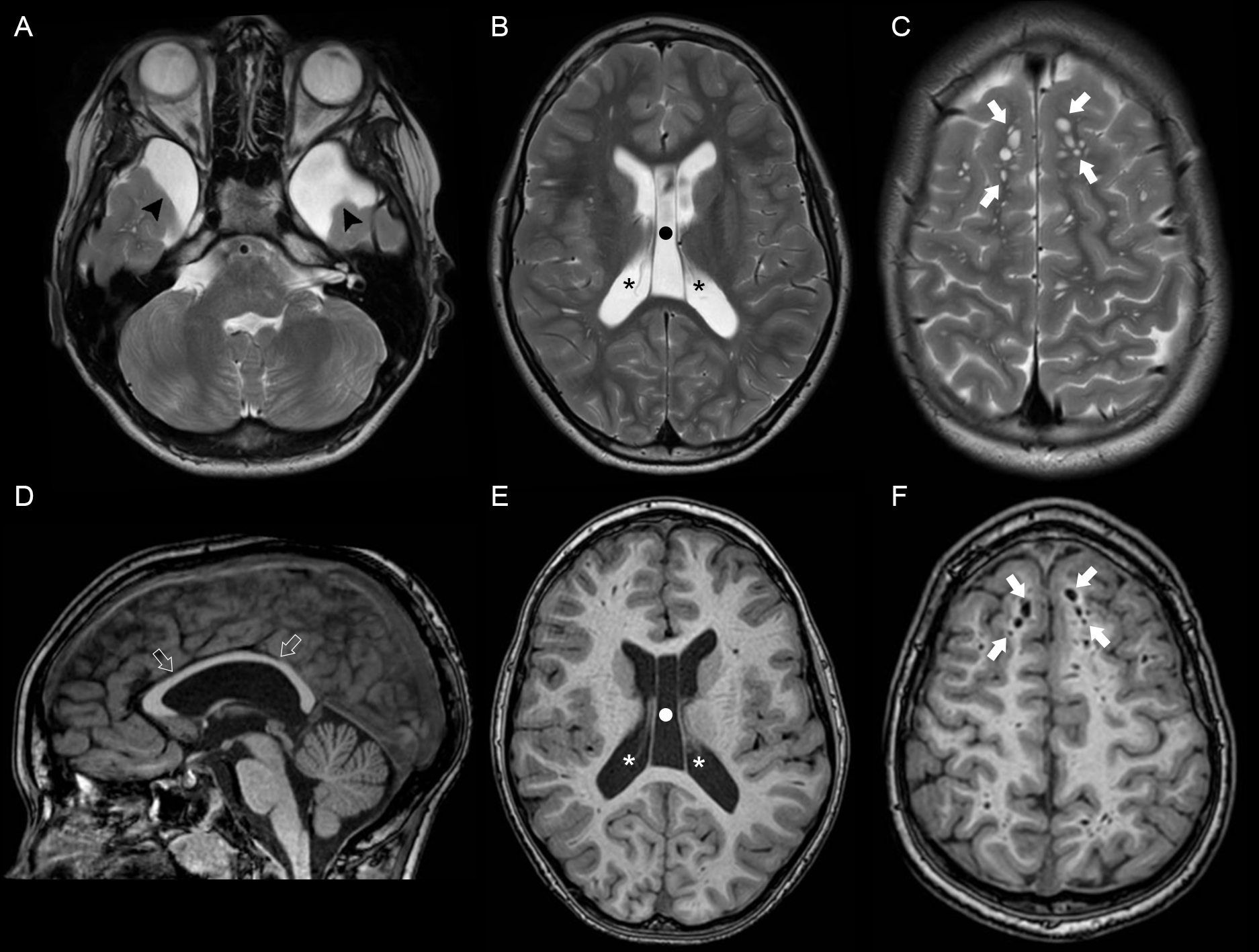

Supplement: Supplementary Figure 3 — Imaging characteristics of Sotos Syndrome. Axial T2WI (A–C) as well as sagittal (D) and axial (E,F) T1WI of an 8-year-old child with Sotos syndrome reveal mild ventriculomegaly (asterisks) and enlargement of the subarachnoid spaces mainly in the temporopolar region (black arrowheads). Also note thinning of the corpus callosum (open arrows) and presence of a cavum septum pellucidum/vergae (circles) as well as dilated perivascular spaces (thick white arrows) mainly in the anterior cerebral white matter. [file Image_3.jpg]
