# Supplementary material for: Diagnostic Approach to Macrocephaly in Children
Source: Front Pediatr. 2022 Jan 14;9:794069. doi: 10.3389/fped.2021.794069 (PMC8795981; doi:10.3389/fped.2021.794069)
Supplement: Supplementary Figure 2 — Imaging approach to true megalencephaly. A, anterior; CC, corpus callosum; GM, gray matter; H-MRS, proton magnetic resonance spectroscopy; HMEG, hemimegalencephaly; MEG, megalencephaly; NAA, N-acetylaspartate; P, posterior; WM, white matter. [file Image_2.pdf]

MACROCRANIA

→ Exclusion of Secondary Causes of Macrocrania

True Megalencephaly

Anatomic/developmental Megalencephaly

Symmetric or asymmetric
Usually stable imaging findings
Other cortical and midline malformations are common

Assess extent and location of the parenchymal enlargement

Involvement of the 2 cerebral hemispheres +/- posterior fossa structures	Bilateral MEG
Enlargement 1 whole cerebral hemisphere	Unilateral MEG/ HMEG
Enlargement 1 whole cerebral hemisphere + ipsilateral brainstem and cerebellum	"Total" HMEG
Enlargement of part of 1 cerebral hemisphere A/P gradients	"Quadrantic dysplasia"/"Lobar HMEG"/ "Hemi-HMEG"

Assess presence of any of the following:

Cortical malformations
White-matter abnormalities
CC and other midline anomalies
Acquired tonsillar ectopia

Metabolic Megalencephaly

Usually symmetric
Frequent imaging changes over time
Other intracranial malformations usually absent
Supporting imaging features: bilateral signal changes in GM/WM, parenchymal cysts, areas of restricted diffusion or contrast enhancement
For specific entities: pattern recognition + clinical features!
H-MRS: may add important info (eg, increased NAA peak in Canavan disease)
