# Supplementary material for: Diagnostic Approach to Macrocephaly in Children
Source: Front Pediatr. 2022 Jan 14;9:794069. doi: 10.3389/fped.2021.794069 (PMC8795981; doi:10.3389/fped.2021.794069)
Supplement: Supplementary Table 1 — Acquired and congenital causes of hydrocephalus with proposed underlying mechanism. [file Table_1.docx]

**Supplemental Table 1**. Acquired and congenital causes of hydrocephalus with proposed underlying mechanism

| **Acquired hydrocephalus** | **Cause** | **Related pathomechanism** |
| --- | --- | --- |
| Subarachnoid haemorrhage or infection | Arachnoid scar | Dysfunctional subarachnoid space |
| Intraventricular haemorrhage or infection | Ependymal scar | Ventricular obstruction |
| Neoplasms | Parenchymal brain tumor | Ventricular obstruction |
|  | Spinal cord tumor | Dysfunction subarachnoid space |
|  | Choroid plexus tumor | CSF overproduction / ventricular obstruction |
| Disordered cerebral venous function | Extrinsic venous obstruction – e.g. skeletal dysplasia; intrinsic venous obstruction – e.g. venous sinus thrombosis, idiopathic venous dysfunction. | Decreased venous compliance or decreased CSF absorption |
| **Congenital or developmental hydrocephalus** |  |  |
| Neural tube defects | Multifactorial, including mutations in the genes of the PCP pathway | Third or fourth ventricle outlet obstruction, altered venous compliance |
| Vascular malformations | Arteriovenous malformations (somatic mutations in genes of RAS-ERK signaling pathway) | Ventricular obstruction, decreased venous compliance or CSF absorption |
|  | Capillary malformation-arteriovenous malformation 1 - RASA1 (AD) |  |
|  | Capillary malformation-arteriovenous malformation 2- EPHB4 (AD) |  |
| Posterior fossa malformations (e.g. Dandy-Walker complex; Chiari I malformation) | Multifactorial, including genetic factors | Obstructive hydrocephalus |
| Congenital foramen of Monro atresia | Lateral ventricle outlet obstruction | Obstructive hydrocephalus |
| L1 syndrome | *L1CAM* (X-linked) | Obstructive hydrocephalus due to aqueductal stenosis |
| Hydrocephalus, congenital, 2, with or without brain or eye anomalies | *MPDZ (AR)* | Communicating hydrocephalus |
| Hydrocephalus, congenital, 1 | *CCDC88C (AR)* | Obstructive hydrocephalus |
| Pettigrew syndrome | AP1S2 (X-linked) | Obstructive due to aqueductal stenosis |
| VACTERL-H | ZIC3 (X-linked) | Obstructive due to aqueductal stenosis |
| RASopathies | *HRAS*, *KRAS*, and *NRAS*,  RAS-ERK signaling pathway (AD) | Mostly obstructive hydrocephalus |

**Legend:** CSF Cerebrospinal fluid, VACTERL-H (V vertebral anomalies, A imperforate anus, C cardiac anomalies, TE tracheoesophageal fistula, K kidney anomalies, L limb anomalies, H hydrocephalus).
